# Growth characteristics of natural microbial populations are skewed toward bacteria with low specific growth rates

**DOI:** 10.1128/spectrum.02193-26

**Published:** 2026-08-04

**Authors:** Ashley N. Bulseco, Wenzhuo Yang, Julie A. Huber, Joseph J. Vallino

**Affiliations:** 1 The Ecosystems Center, Marine Biological Laboratory 541653, Woods Hole, Massachusetts, USA; 2 Marine Chemistry and Geochemistry, Woods Hole Oceanographic Institution 830239, Woods Hole, Massachusetts, USA; 3 Department of Biological Sciences, University of New Hampshire 169218, Durham, New Hampshire, USA; 4 Environmental Science and Sustainability Department, Allegheny College 384056, Meadville, Pennsylvania, USA; Connecticut Agricultural Experiment Station, New Haven, Connecticut, USA

**Keywords:** maximum specific growth rate, growth limits, chemostat, trait-based model, oligotroph, copiotroph, gleaner, opportunist, dilution rate, amplicon sequencing

## Abstract

Microbes are often functionally characterized by traits that specify their optimum environmental conditions for growth, such as temperature or pH, as well as upper and lower bounds where growth is possible. While any given microbe will have a narrow environmental window where growth can occur, a diverse community can span a much larger range of conditions where growth is possible by at least some members of the community. One important trait of microbes is maximum specific growth rate, as this trait determines if a microbe can persist in environments with short residence times. In this study, we conducted a chemostat experiment with natural microbial communities and manipulated dilution rate to test how it would act as a selective force controlling community dynamics and microbial diversity. Using both the experimental chemostats, as well as trait-based modeling, we examined how the composition of a microbial community collected from a coastal meromictic pond changed as dilution rate increased from 0.1 to 10 day^−1^. We compared experimental results from 16S rRNA gene amplicon sequences to the output from two different simulations of the trait-based model, one in which maximum specific growth rate was initially evenly distributed across the community, and another where maximum specific growth rate traits were pulled from a beta probability distribution that was skewed toward low specific growth rates. Experimental results matched the simulation where the initial natural population was dominated by slow-growing microbes, highlighting the importance of initial trait distributions in modeling community response to environmental changes.

**IMPORTANCE:**

All living organisms are constrained by environmental boundaries that govern where growth is possible, such as minimum and maximum temperatures or pH. An organism’s maximum specific growth rate places a lower bound on the residence or turnover time where an organism can persist without being removed from the system. While there are laboratory constraints on the maximum specific growth rate of culturable microorganisms, such as *Escherichia* or *Vibrio* species, information is lacking on how the maximum specific growth rate trait is distributed in natural microbial communities. This information is critical for understanding how a community will respond to changes in residence time or other environmental perturbations. Using chemostats and trait-based modeling, this study assessed how maximum specific growth rate is distributed in a natural community collected from a coastal, salinity-stratified pond.

## INTRODUCTION

Microbes are among the most abundant, diverse, and ubiquitous organisms on Earth. Their interactions mediate important biogeochemical cycles, influencing ecosystem processes, such as nutrient fluxes, productivity, and carbon cycling (1). A major goal of microbial ecology is to understand and predict what ecological mechanisms drive biodiversity, structure, and dynamics in microbial communities, as these characteristics are linked to ecosystem function and biogeochemistry. Research on multicellular organisms has demonstrated how changes in biodiversity are driven by environmental gradients, such as temperature, depth, or elevation (2). Similarly, in microbiology, habitats select for microorganisms with specific functional traits (3), and as habitats approach extremes in temperature, salinity, pH, or other boundaries that define where life can exist, the diversity of microbial communities decreases and becomes dominated by only a few taxa best adapted to the given extreme condition (4). Where boundaries are crossed, life is excluded. For instance, Sharp et al. (5) found a strong unimodal decrease in microbial diversity (Shannon index) at temperature extremes, and a similar, but weaker, relationship with pH extremes from terrestrial springs. In arable soil, Rousk et al. (6) found a strong, positive relationship between bacterial diversity and pH, with effectively half the number of operational taxonomic units existing at pH 4 compared to a pH of 8.3. Similar patterns of low diversity have been described in other extreme temperature environments, such as hot springs in Yellowstone (7, 8) and hydrothermal vent systems (9), as well as in acidic mine-drainage habitats (10–12).

Resource supply rate and associated transport processes are another type of extreme that planktonic organisms must contend with. In systems subject to advective and diffusive transport (13), such as streams, lakes, and meso-scale eddies, turnover rate (day^−1^) is defined by the volumetric rate of water entering and leaving the system (e.g., m^3^ day^−1^) divided by the volume of the system (m^3^). Another often-used metric, residence time (days), is the inverse of turnover rate. For microbes, the specific growth rate (day^−1^) is defined by growth rate (cells L^−1^ day^−1^) divided by their abundance (cells L^−1^). Consequently, an organism’s specific growth rate must match or exceed the water turnover rate; otherwise, it will be transported out of the system regardless of whether or not it is a good competitor. Since specific growth rate depends on resource concentration (14), an organism can persist in a system with high water turnover rates provided resource concentration is high and water turnover rate does not exceed the organism’s maximum specific growth rate. Maximum specific growth rate and resource affinity are important in structuring microbial communities, as competitive abilities between organisms depend on both processes and have been widely studied in ecology. For instance, the trade-off between maximum growth rate and resource affinity, the r-K strategy dichotomy (15, 16), or the similar R* rule (17), describes a set of metabolic strategies that favor either high resource affinity or high maximum growth rates, but follow a continuum from “gleaners” to “opportunists,” respectively (18–20). Transport processes play an important role in competition as well, and Locey and Lennon (21) provide a review of theories governing residence time and biodiversity.

Predictive models can be used to study changes in biodiversity as a function of environmental drivers (22), but simulating the full capabilities of natural systems can be challenging given the high diversity in microbial systems (23). Furthermore, a large fraction of microbes remain uncultured (24), and the growth kinetics of most bacteria are unknown, which makes explicit model representation of all microbial species present in a system impossible. Trait-based modeling approaches aim to identify and improve our understanding of mechanisms underlying ecological patterns that structure microbial communities through the exploration of functional traits, instead of explicit representation of individuals (20, 25–27). These traits, which can be morphological, physiological, or genomic, incorporate physiological trade-offs and thus constrain the performance of an organism under a set of given environmental conditions (28, 29). Organisms with particular functional traits that are better suited for a given environment are favored, and this environmental selection ultimately determines the structure of the microbial community and the dominant ecosystem processes (30, 31), as well as how these linkages may change in the future, provided trade-offs between traits are properly understood (20).

Trait-based models can be used to study competitive interactions between microbes for resources that are subject to transport processes, but how traits are initialized and distributed across the community is important to consider as the communities approach conditions near growth extremes that are a function of maximum specific growth rate and substrate affinity. For instance, for a natural microbial community, we can define a trait variable that governs the trade-off between high specific growth rate and high substrate affinity that represents a continuum from oligotrophs using ATP-binding cassette transporters at one end of the spectrum to copiotrophs using phosphotransferase systems at the other end (32). How is this trait distributed in natural communities with high richness? Are copiotrophs just as likely as oligotrophs, or is the community skewed toward one side or the other? The distribution of this trait should impact community composition in high turnover systems as the growth rate boundary is approached and selects for microbes with high specific growth rates.

To test how specific growth rate and substrate affinity traits might act as a selective force controlling microbial community dynamics and diversity, we conducted a chemostat experiment in which we varied the dilution rate using a natural microbial population collected from a coastal pond. The use of a chemostat experiment is advantageous because the convergence of cellular growth and dilution rate at steady state allows for precise characterization of microbial dynamics under a given environmental condition (33), and the net specific growth rate of microbes (accounting for predation) must be able to match or exceed the chemostat’s dilution rate; otherwise, they will be washed out. Here, we hypothesized that as dilution rate increases, the diversity of the community will decrease because natural communities are skewed toward oligotrophic strategies. In contrast, if the microbial community has a uniform distribution in specific growth rate versus substrate affinity trait, then the number of bacteria capable of growth at low dilution rates will equal those that can grow at high dilution rates. For finite-time experiments, the diversity should not change significantly as dilution rate increases, although community composition will change. To test our hypothesis, we developed a simple trait-based model that predicts changes in biodiversity assuming either (i) a uniform probability distribution of bacterial growth kinetic traits (null hypothesis) or (ii) a beta probability distribution of traits favoring lower specific growth rates, mimicking conditions we hypothesized to be present in nature. By comparing empirical observations with the trait-based model simulation, we found that diversity diminished as the growth rate boundary was approached in both experimental and model results using the skewed distribution, supporting the hypothesis that the microbial community was weighted toward oligotrophs (aka, gleaners). Our results also demonstrate the importance of trait initialization on the performance of trait-based models.

## MATERIALS AND METHODS

### Experimental setup

We collected a 10 L water sample at 2.5 m below the surface from a coastal, meromictic pond (Sider’s Pond in Falmouth, MA, USA; 41°32′52.08″ N, −70°37′26.82″ W), which is permanently stratified and characterized as both eutrophic (34) and phosphorus limited (35). *In situ* water conditions were 12.8°C with salinity and pH of 5.48 and 7.09, respectively. After passing water through a 300 μm Nytex mesh to remove large organisms and particles, we inoculated two replicate (MC1 and MC2) 3 L bioreactors (Bellco glass: 1964-03600, outfitted with PTFE low shear impellers 1964-03404) with 3 L of Sider’s Pond water. We chose to start with 100% pond water, rather than inoculating a defined medium to maximize inclusion of the rare biosphere (23) and functional diversity (36).

Environmental operating conditions were maintained at 25°C in the dark by an environmental chamber (PGR15 Conviron, Winnipeg, Canada), and chemostats were sparged with synthetic air from a gas cylinder (21.06% O_2_, 0.04% CO_2_, balance N_2_) at a flow rate of 10 sccm (10.92 mL min^−1^ at 25°C and 101.3 kPa) regulated with mass flow controllers (MKS M100B, Andover, MA, USA). To prevent growth in the medium feeding the chemostats, it was divided into two parts consisting of a nutrient-only and carbon-only media. The nutrient medium (NM) consisted of 15 μM KNO_3_, 2 μM HK_2_PO_4_, 100 μM MgSO_4_ 7H_2_O, 100 μM CaCl_2_ 2H_2_O, 1 mL of a concentrated trace element (TE) solution per liter of NM (37), and adjusted to a salinity of 3 PSU using Instant Ocean Sea Salt. The TE solution contained 18.5 mM FeCl_3_ 6H_2_O, 485 μM H_3_BO_3_, 126 μM CoCl_2_ 6H_2_O, 100 μM CuSO_4_ 5H_2_O, 348 μM ZnSO_4_ H_2_O, 163 μM MnSO_4_ H_2_O, 124 μM Na_2_MoO_4_ 2H_2_O, 84 μM NiCl_2_ 6H_2_O, and 10 mL L^−1^ 37% HCl. The carbon medium (CM) consisted of 5.0 mM glucose, 5.969 mM xylose, 10.83 mM ethanol, 16.75 mM acetate, and 20.61 mM methanol, where carbon substrate concentrations were chosen such that each substrate contributed 14.7 kJ L^−1^ Gibb’s free energy at 293 K and pH 6.8 to the total (38). Dedicated pumps (Masterflex 07523-90 drive, Easy-Load 3 pump head, and L/S Norprene tubing) were used for the NM and CM, and the CM pump was also fitted with two additional pump heads (four in total) with larger L/S tubing, which were used to withdraw media from the chemostats to maintain constant bioreactor volume. To manipulate resource availability, we supplied the nutrient plus carbon media at three different dilution rates and two different media ratios (CM:NM) as follows: 0.1 day^−1^ (0.3 L day^−1^) with a 1:98.3 CM:NM ratio; 1.11 day^−1^ (3.33 L day^−1^) with a 1:8.24 CM:NM ratio; 10 day^−1^ (30 L day^−1^) with a 1:10 CM:NM ratio. The experiment started at the 0.1 day^−1^ dilution rate and stepped up to 1.0 and 10 day^−1^ sequentially, which were run for 15.5, 5.3, and 3 days, respectively (Table 1). Note, the change in the CM:NM ratio for the different dilution rates was unintended and was caused by a software bug in the pump controlling the CM. Because the media at 1:100 was designed to be N limited, the increase in CM did not change this limitation, so it has minimum impact on the experiment.

**TABLE 1:** Experimental chemostat conditions^*a*^

Dilution rate (day^−1^)	CM flow (L day^−1^)	NM flow (L day^−1^)	CM:NM	Days run (days)	Day of collection
0.1	0.0030	0.30	1:98.3	15.5	0 (pond), 0.8, 5.8*, 9.5*, 10.4, 15.4
1.1	0.36	2.97	1:8.24	5.3	16.5, 17.6, 18.8, 19.8
10	2.73	27.3	1:10.0	3	21.5, 21.8, 22.4, 22.8*, 23.5

^*a*^ Samples with asterisks were sequenced successfully in only one of the two chemostat replicates. CM, carbon medium; NM, nutrient medium; CM:NM, ratio between the carbon and nutrient medium.

### Sample collection and biogeochemical analyses

Every hour, the chemostat monitoring system sampled for gas concentrations of carbon dioxide (CO_2_) and oxygen (O_2_) in the headspace via a closed gas-sampling loop (250 mL min^−1^ total volume) containing a Nafion gas dryer (Perma Pure MD-070-24P, Lakewood, NJ, USA) and an Oxigraf laser diode absorption spectrometer (O2Cap, Sunnyvale, CA, USA). Chemostats were sequentially gas sampled using a computer-controlled Valco (Houston, TX, USA) STF selector valve, which also sampled the feed gas cylinder, thereby allowing a single-point calibration of the O2Cap gas analyzer to account for instrument drift. We monitored dissolved oxygen (DO) and pH with a Hamilton (Reno, NV) VisiFerm DO Arc 335 and EasyFerm Plus Arc 325 probes, respectively, and controlled and calibrated the probes via Hamilton Device Manager software equipped with a USB-ModBus RS 485 converter.

At each sample time point (Table 1), we withdrew no more than 500 mL total volume from each chemostat. Using a 60 mL syringe, we filtered the sample through a Whatman GF/F filter for nitrate (NO_3_^−^), ammonium (NH_4_^+^), and phosphate (PO_4_^3−^), as well as dissolved organic carbon (DOC), which we preserved with 100 µL of 50% HCl, and stored at −20°C until analysis. For bacterial enumeration, we collected 10 mL of whole water and stored samples at 4°C preserved with 3.7% formaldehyde (final concentration). For 16S rRNA gene amplicon sequencing, we filtered between 60 and 250 mL of water onto a 0.2 µm polyethersulfone (PES) Sterivex filter (until the filter clogged), which we stored at −80°C until extraction.

We analyzed NO_3_^−^ and PO_4_^3−^ concentrations on a Lachat (QuikChem 8500) and NH_4_^+^ concentrations on a Shimadzu 1601 spectrophotometer using a modified phenol-hypochlorite method (39). DOC was measured in duplicate on a total organic carbon analyzer (Aurora 1030W, OI Analytical) after sample acidification.

### Bacterial cell counts, library preparation, and sequence analysis

We performed direct bacterial cell counts on water samples. Following methods outlined in Porter and Feig (40), we filtered 1 mL of sample volume onto a 25 mm, 0.2 µm polycarbonate filter backed by a 0.45 µm GFF filter and fluorescently stained bacterial cells with DAPI (28 µM final concentration, Sigma-Aldrich) for 5 min in the dark. We visualized bacterial cells using an epifluorescence Zeiss Axio Imager 2 and performed counts using the 100× objective on five fields of view. We extracted genomic DNA from PES filters using the DNeasy PowerSoil Pro kit (Qiagen) following manufacturer’s methods and submitted high-quality extracts to the Bay Paul Center at the Marine Biological Laboratory for library preparation and sequencing. Amplicon libraries were prepared using bacterial 16S V4V5 515F (CCAGCAGCYGCGGTAAN) and degenerate 926R primers (CCGTCAATTCNTTTRAGT or CCGTCAATTTCTTTGAGT or CCGTCTATTCCTTTGANT) using Platinum HiFi II DNA Polymerase (Thermo Fisher) and purified using AMPure Beads (Beckman Coulter) at a 0.75× bead-to-template ratio. After quantifying the final pool using a Kapa Biosystems qPCR (Millipore Sigma), sequencing commenced on an Illumina MiSeq, generating 250 nt paired-end reads. These reads can be found in the NCBI Sequence Read Archive under BioProject ID PRJNA1103251.

We used the DADA2 workflow (v1.14.1; [41]) in R (v3.6.3; [42]) to analyze our sequence data. To perform quality filtering, we applied the following parameters in the “filterAndTrim” command: MaxN = 0, maxEE = 2 and 2, truncQ = 2, truncLen = 250 and 225 (based on quality profiles), and trimLeft = 19 and 20, for forward and reverse reads, respectively. After inferring amplicon sequence variants (ASVs) from de-replicated reads, we removed chimeric sequences using the “consensus” method. We assigned taxonomy against the SILVA database (v138; [43]) and filtered out any sequences matching Mitochondria or Chloroplasts due to known primer biases, resulting in 1,440 unique ASVs (Table S1). We conducted all downstream sequence analyses on generated ASV tables using the Phyloseq package (v1.30.0 [44]).

### Trait-based model description

To model the population dynamics of a community of bacteria, we used a previously developed adaptive Monod equation that captures prokaryote growth kinetics across oligotrophic to copiotrophic conditions (45) as the basis for our simulations. Under oligotrophic conditions, bacteria have low specific growth rates (<1 day^−1^), low growth efficiencies (<10%), but high substrate affinities (nM substrate concentrations). While under copiotrophic conditions, bacterial specific growth rates can exceed 50 day^−1^, growth efficiencies can exceed 60%, but substrate affinity is low (mM substrate concentrations). In the adaptive Monod kinetics equation, specific substrate uptake rate, φ, is governed by a growth efficiency parameter, ε, that varies between 0 and 1 to represent growth kinetics across the full range of trophic environments a community may experience, which is given by equation 1,


(1) $$\varphi \left(s;\varepsilon \right)=\nu^{*}\varepsilon^{2}\left(\frac{s}{s+\kappa^{*}\varepsilon^{4}}\right)\left(1-\varepsilon^{2}\right),$$

where s is substrate concentration (μM), and the community-independent constants $\nu^{*}$ and $\kappa^{*}$ are fixed at values of 350 day^−1^ and 5,000 μM, respectively. These two constants have been used successfully for modeling prokaryote growth in both laboratory and field environments (46–48). To reduce the complexity of the model, the last term in equation 1, $\left(1-\varepsilon^{2}\right)$, is an approximate thermodynamic constraint that replaces the formal thermodynamic driver used previously (49). The specific growth rate, μ, is simply the product of ε and φ(s;ε), where bacterial growth is governed by the following stoichiometric equation 2,


(2) $$CH_{2}O+\left(1-\varepsilon_{i}\right)O_{2}\to \;\varepsilon_{i}x_{i}+\left(1-\varepsilon_{i}\right)\left(CO_{2}+H_{2}O\right),$$

where $x_{i}$ is bacterial biomass concentration in μM C for instance i, which represents a single ASV. For simplicity, bacterial growth is assumed to be solely limited by glucose, $CH_{2}O$, normalized to unit carbon here. We use trait-based modeling (20, 25) to simulate the population of bacteria in the experimental chemostats where the growth efficiency, $\varepsilon_{i}$, is a bacterial trait variable that can be assigned a value randomly between 0 and 1. This value represents a continuum of ecological strategies varying from gleaner $\left(\varepsilon_{i}\approx 0\right)$ to opportunist $\left(\varepsilon_{i}\approx 1\right)$. Letting $x_{i}(t)$ be the concentration of a single prokaryote species (or ASV) with trait $\varepsilon_{i}$ at time t, the equations governing a population of n prokaryotes each with randomly assigned growth efficiency traits in a chemostat are given by the following four equations,


(3) $$\frac{dx_{i}\left(t\right)}{dt}=D\left(t\right)\left(x_{i}^{f}-x_{i}\left(t\right)\right)+\varepsilon_{i}\varphi \left(s\left(t\right);\varepsilon_{i}\right)x_{i}\left(t\right)-m\frac{x_{i}\left(t\right)}{x_{T}\left(t\right)}x_{i}^{2}\left(t\right),$$

(4) $$\frac{ds\left(t\right)}{dt}=D\left(t\right)\left(s^{f}-s\left(t\right)\right)-\sum_{i=1}^{n}\varphi \left(s\left(t\right);\varepsilon_{i}\right)x_{i}\left(t\right),$$

(5) $$\frac{dO_{2}\left(t\right)}{dt}=D\left(t\right)\left(O_{2}^{f}-O_{2}\left(t\right)\right)+\frac{k_{L}a}{v_{L}}\left(p_{O2}\left(t\right)h_{O2}-O_{2}\left(t\right)\right)-\sum_{i=1}^{n}\left(1-\varepsilon_{i}\right)\varphi \left(s\left(t\right);\varepsilon_{i}\right)x_{i}\left(t\right),$$

(6) $$\frac{dp_{O2}\left(t\right)}{dt}=\frac{f_{G}}{v_{G}}\left(p_{O2}^{f}-p_{O2}\left(t\right)\right)-\frac{k_{L}a}{v_{G}}RT\left(p_{O2}\left(t\right)h_{O2}-O_{2}\left(t\right)\right),$$

where, m is a mortality constant, $x_{T}\left(t\right)$ is the total bacterial biomass $\left(x_{T}\left(t\right)=\sum_{i=1}^{n}x_{i}\left(t\right)\right)$, $O_{2}$ is DO concentration (μM), $p_{O2}$ is the partial pressure of oxygen in the chemostat headspace (atm), $k_{L}$ is the liquid-side gas transfer velocity (m day^−1^), a is the area of the gas-liquid interface (m^2^), $v_{L}$ and $v_{G}$ are the volumes of the liquid and gas spaces (m^3^), respectively, $h_{O2}$ is the Henry’s law constant for O_2_ in water (μM atm^−1^) (50), $f_{G}$ is the gas flow rate (m^3^ day^−1^), superscript f denotes the state variable concentration or pressure in the input feed, n is the number of ASVs in the simulated population, where the values of $\varepsilon_{i}$ are randomly drawn from an appropriate probability distribution, and D(t) is the dilution rate (day^−1^), set to match the experimental dilution rate over time. We include DO and O_2_ partial pressure in the model to compare with chemostat observations. This allows us to assess the performance of the model relative to the experiment and helps ensure that the model reasonably captures observed process rates. The liquid-side gas transfer velocity, $k_{L}$, was determined by sparging an operationally sterile reactor with N_2_ until DO was below 25 μM, then rapidly equilibrated the headspace with air and monitored the increase in DO over time while sparging the chemostat with air at the operational flow rate. This process was modeled using equation 5, with $p_{O2}\left(t\right)$ set to atmospheric concentration and $\varphi \left(s\left(t\right);\varepsilon_{i}\right)$ and D(t) set to 0. The value of $k_{L}$ was determined by minimizing the error between predicted and measured DO over time.

Since we did not measure protist and phage abundances, we did not include explicit state variables representing bacterial predators. Instead, we investigated several types of bacterial mortality closure terms (51, 52) and chose a quadratic form (equation 3) weighted by the relative abundance of an ASV $\left(x_{i}(t)/x_{T}(t)\right)$. Quadratic mortality is a kill-the-winner scheme (53) that better represents phage mortality that is likely the dominant source of bacterial predation in the chemostats (54). Furthermore, nonlinear closure terms prevent competitive exclusion from occurring (55), so coexistence between bacteria is observed when simulations are run for 100s of days to steady state. The mortality constant, m, was tuned using the chemostat DO and O_2_ gas data and set to 0.1 m^3^ mmol^−1^ day^−1^.

**Fig 1:**
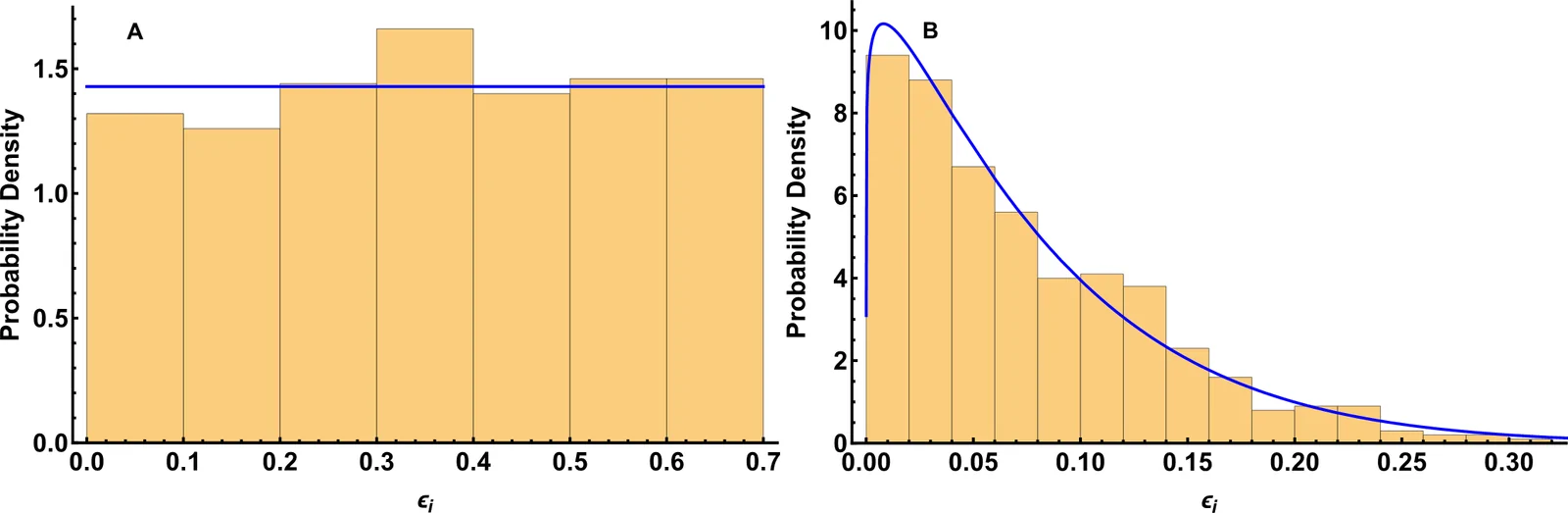
Probability density function (blue lines) and histograms (yellow bars) of 500 samplings of $\varepsilon_{i}$ (growth efficiency of ASV $x_{i}$, equation 1) drawn from (**A**) a uniform distribution over 0 to 0.7 and (**B**) a beta distribution with α = 1.1, β = 13.3 (**B**). Note, for the uniform distribution $\varepsilon_{i}$ was limited to 0.7 because the specific uptake rate for the adaptive Monod equation, equation 1, begins to decrease for larger values of $\varepsilon_{i}$ due to a decrease in the thermodynamic driver. Values of $\varepsilon_{i}$ > 0.7 are theoretically possible, but not evolutionarily competitive. Values of $\varepsilon_{i}$ represent a continuum of ecological strategies varying from gleaner $\left(\varepsilon_{i}\approx 0\right)$ to opportunist $\left(\varepsilon_{i}\approx 1\right)$. These plots are from sample 1 distributions.

The two model scenarios we investigated were (i) $\varepsilon_{i}$ randomly drawn from a uniform probability distribution, ranging from 0 to 0.7 (Fig. 1A), and (ii) $\varepsilon_{i}$ drawn from a beta probability distribution with α = 1.1, β = 13.3 between 0 and 1, which is heavily skewed toward the oligotrophic end of the distribution (Fig. 1B). The uniform distribution was limited to $\varepsilon_{i}\leq 0.7$, because specific growth rate decreases for larger values of $\varepsilon_{i}$ due to the thermodynamic driver [$\left(1-\varepsilon^{2}\right)$ term in equation 1], but $\varepsilon_{i}$ in the beta distribution do not approach that limit (Fig. 1B). The first scenario represents a null test of the hypothesis, in that it assumes there is no decrease in the diversity of specific growth rates as the upper limit for prokaryote growth is approached. In contrast, the second scenario assumes there are disproportionately fewer members of the microbial population that have specific growth rates near the maximum possible value of 65 day^−1^ based on equation 1. That is, a community skewed toward gleaners.

Simulations were run for each scenario consisting of 500 ASVs (n = 500), where the substrate feed concentration, $s^{f}$, was set to 600 μM in an otherwise sterile medium ($x_{i}^{f}=0$), and the initial concentrations of s(0) and $x_{i}(0)$ were set to 0 and 10^−5^ μM, respectively. The dilution rate was stepped up from 0.1 to 1.0, and then 1.0 to 10 day^−1^ based on experimental event times (Table 1). We compare simulated bacterial ASVs, $x_{i}(t)$, partial pressure of oxygen in the gas headspace, $p_{O2}(t)$ (%), and dissolved oxygen, $O_{2}\left(t\right)$ (μM), to experimental results to determine if the specific growth rate distribution of prokaryotes in the chemostat is better described by uniform versus beta probability distributions. To facilitate statistical analysis of model output, two simulations (samples 1 and 2) were run for each scenario based on two random samplings from a uniform or beta probability distribution to set $\varepsilon_{i}$, producing four runs in total. Simulations were conducted with Mathematica, and the notebook and supporting materials are posted in a Github repository (https://github.com/maxEntropyProd/Trait-Based-Chemostat) and archived in Zenodo (https://zenodo.org/records/20075821).

### Statistical analyses

All statistical analyses were conducted in R (v3.6.3; 42). We compared chemostat parameters (e.g., %CO_2_, %O_2_, DO, and pH) and dissolved nutrients (e.g., NO_3_^−^, NH_4_^+^, PO_4_^3−^, and DOC concentrations) across dilution rates using general linear mixed models (GLMMs) with the “lme4” package (56), which accounts for repeated measures. We constructed candidate models with dilution rate and a “day within phase” term, defined by resetting elapsed time to zero at the start of each dilution regime, as fixed effects, and included “chemostat ID” as a random effect. This temporal structure allowed us to distinguish differences among dilution rates from transient, within-regime dynamics following treatment changes. The four candidate models we examined included: an additive model (dilution rate + day within phase), an interaction model (dilution rate × day within phase), and two simpler models (dilution rate only and a null model). We identified the most parsimonious model of best fit by calculating a second-order Akaike Information Criterion (AIC) corrected for small sample sizes using the “AICc” and “model.sel” commands in the “MuMIn” package (57). Model selection considered AICc, the difference in AICc relative to the model with the lowest value (ΔAIC), and the Akaike weights (*w*_*i*_), which represent the likelihood of the model normalized by the sum of all models with values closest to 1, indicating the best fit (58). Following model selection, we examined multiple comparisons of main effects and slopes, when the interaction model was supported, among dilution rates with the “emmeans” package (59).

To visualize microbial community structure at each sample point, we calculated the relative abundance (%) of ASVs. We only included those ASVs with abundances greater than 0.1% in stacked bar plots because we were most interested in large-scale shifts in community structure. We calculated Bray-Curtis dissimilarity on our full data set with the pond sample removed to quantify diversity between MC samples and visualized the results using a principal coordinates analysis. To estimate dispersion among samples by dilution rate, we calculated the non-Euclidean distance between each sample and group centroids using the “betadisper” function in the vegan package (60) and compared dispersion among dilution rates using a GLMM as described above. To better understand microbial community stability between samples, we calculated Bray-Curtis pairwise distance between sequential time points (sampling point 1 compared to 2, 2 to 3, etc.) within dilution rates. This resulted in a dissimilarity metric, which we then converted to similarity by subtracting 1 from each value such that increasing values indicate greater community stability between samples. That is, a community stability index close to 1 indicates that the community composition does not change significantly between two sequential time points. We then calculated alpha diversity on both rarified and non-rarified data sets using the Shannon index in Phyloseq and compared community stability and Shannon index across dilution rates using GLMMs. Similar analyses also were conducted on simulated ASVs from our trait-based model, except we treated the model replicate (sample 1 vs 2) as the random intercept rather than chemostat ID (MC1 vs MC2).

## RESULTS

### Chemostat biogeochemistry

**Fig 2:**
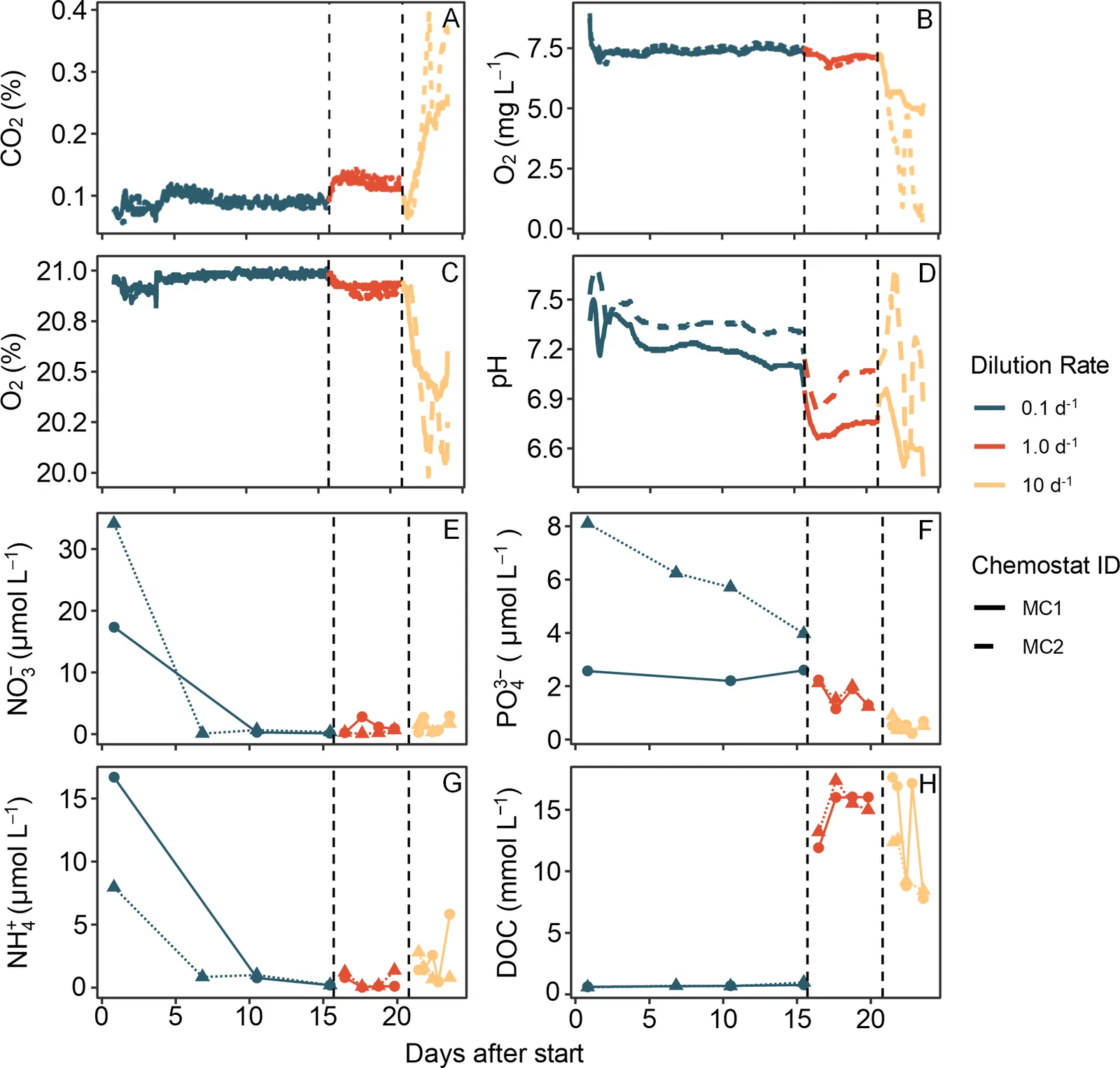
Measurements of (**A**) carbon dioxide (%), (**B**) dissolved oxygen (mg L^−1^), (**C**) oxygen (%), (**D**) pH, (**E**) nitrate (µmol L^−1^), (**F**) phosphate (µmol L^−1^), (**G**) ammonium (µmol L^−1^), and (**H**) DOC (mmol L^−1^) for MC1 (solid line) and MC2 (dashed line), where colors and dotted vertical lines denote dilution rate changes. Samples for microbial community analyses were collected at the same time as sampling time points for nutrient data.

Chemostats were continuously monitored for carbon dioxide and oxygen in the headspace, and dissolved oxygen and pH in the media. Percent CO_2_ averaged 0.09%, 0.12%, and 0.20% (Fig. 2A), percent O_2_ averaged 21.0%, 20.9%, and 20.5% (Fig. 2C), and dissolved O_2_ averaged 7.43, 7.10, and 5.18 mg L^−1^ (Fig. 2B) for 0.1, 1, and 10 day^−1^ dilution rate periods, respectively. Percent CO_2_ was different between the two highest dilution rates (*P* < 0.001) but not between 0.1 and 1.0 day^−1^, while percent O_2_ and dissolved O_2_ were significantly different across all dilution rates (Table S2; *P* < 0.001). Based on input and output O_2_ gas concentrations to each chemostat and steady-state conditions, the rates of oxygen metabolism per liter of media were 0.171, 0.291, and 1.31 mmol O_2_ day^−1^ L^−1^ in MC1 and 0.175, 0.372, and 1.69 mmol O_2_ day^−1^ L^−1^ in MC2 for the 0.1, 1.0, and 10 day^−1^ dilution rate periods, respectively. Model results indicated that CO_2_ and O_2_ temporal dynamics also differed significantly among dilution rates, with time-dependent trajectories showing that the 10 day^−1^ dilution rate exhibited steeper increases in CO_2_ with simultaneous declines in O_2_ compared to lower dilution rates (*P* < 0.001). Dissolved oxygen similarly exhibited strong dilution-dependent temporal dynamics, with steady-state values differing among all three dilution rates. Dissolved oxygen was lowest at the highest dilution rate and closest to saturation at the lowest dilution rate. pH differed significantly among dilution rates with averages at 7.29 for 0.1 day^−1^, 6.86 for 1 day^−1^, and 6.43 for 10 day^−1^ (Fig. 2D). Similar to dissolved oxygen, slopes differed among all three dilution rates, with the greatest rate of change in the highest dilution rate (*P* < 0.001). While community metabolism, based on DO, pH, O_2_, and CO_2_, was stable for most of the experiment, substantial instability occurred between days 21 and 24 in MC2 at a dilution rate of 10 day^−1^ as evident in the online measurements (Fig. 2A through D, dashed lines). We also note that the chemostats at the lowest dilution rate only experienced 1.5 reactor volume turnovers, so may not have reached steady state compared to the higher dilution rates of 1 and 10 day^−1^ that had 5.8 and 27 turnovers, respectively. At the functional level based on online measurements (Fig. 2), the bioreactors appear to be at steady state at the 0.1 day^−1^ dilution rate.

Nitrate, ammonium, and phosphate started at initially high concentrations, reflecting the conditions in Siders Pond (Fig. 2E through G). Additive models for each constituent supported this, with highest concentrations at 0.1 day^−1^ (*P* < 0.001) relative to 1 and 10 day^−1^, which were not significantly different from each other (Table S2). By the end of the low dilution rate period at 15.5 days, concentrations of all three inorganic nutrients had been reduced due to microbial growth and turnover of the initial pond water with feed media and thus remained at low concentrations for the duration of the experiment, as the medium was designed to be N limited. DOC concentration started low and remained so for the 0.1 day^−1^ dilution period, but then increased significantly as dilution rate increased and the media CM:NM ratio (ratio between the carbon, CM, and nutrient, NM, medium) decreased from 100:1 to 10:1 (Fig. 2H; *P* < 0.001). There was no difference in DOC concentrations between the 1 and 10 day^−1^ dilution rates.

### Chemostat microbial community

**Fig 3:**
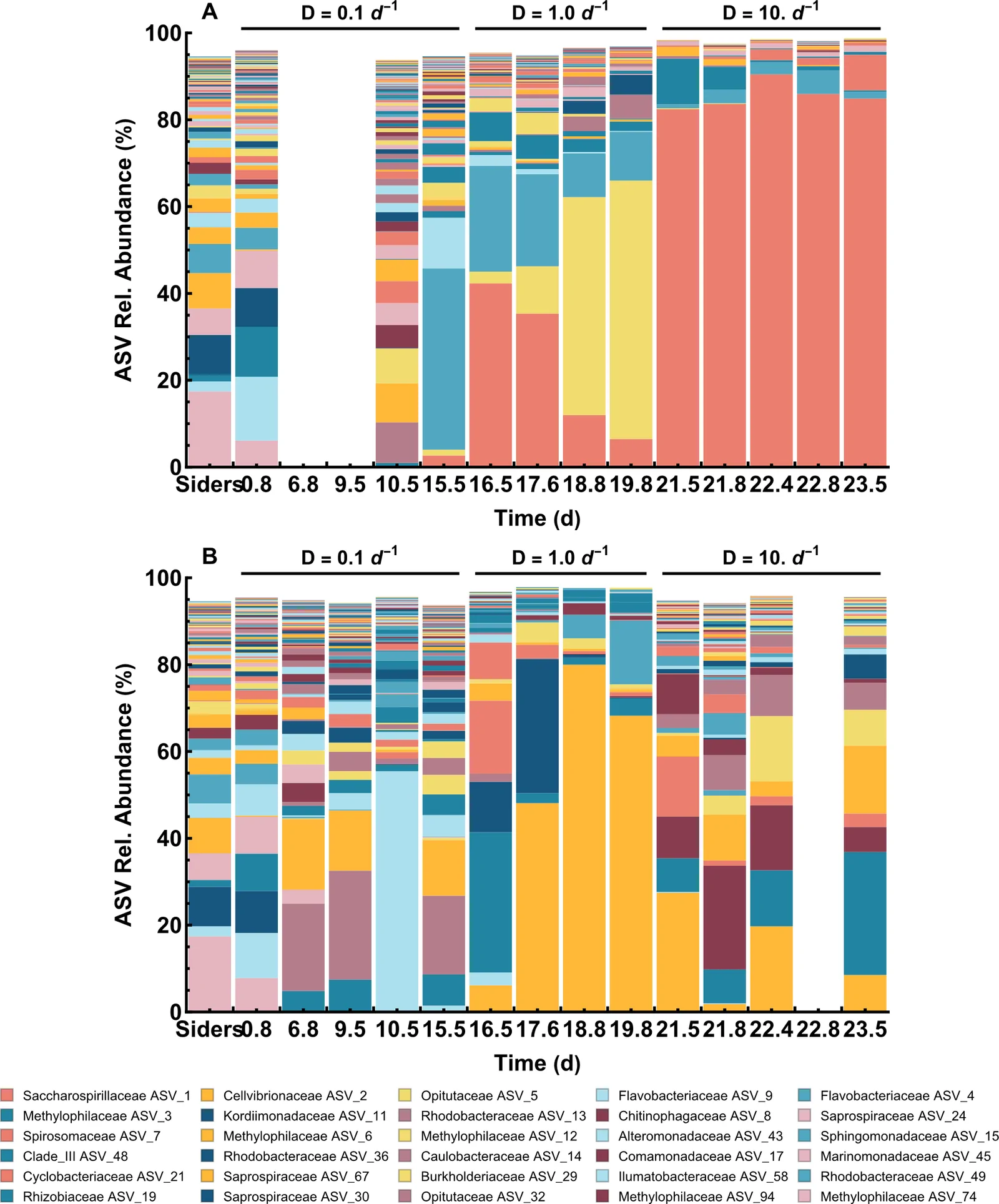
Relative abundance (%) of ASV families from (**A**) MC1 and (**B**) MC2 versus time, along with ASVs from Siders Pond (“Siders” at time 0). Only ASVs that have an abundance greater than or equal to 0.1% are shown. The legend lists the top 30 ASV families across all samples, and blank columns are for samples that did not sequence. The dilution rate and time of sample collection for each chemostat are indicated along the top and bottom, respectively.

Across the two MCs, we sequenced 26 samples for the 16S rRNA gene representing 16 time points, including the initial pond sample. After removing chimeras, 3,550,777 sequences remained, resulting in 1,702 unique ASVs. After removing taxa matching Mitochondria or Chloroplasts, we were left with 1,440 ASVs, 31 of which had a relative abundance (RA) greater than 5% in at least one sample (Fig. 3, includes ASVs at or above 0.1% RA; Table S1, includes all ASVs). The sample collected from Sider’s Pond consisted of taxa belonging to families Saprospiraceae (~20%), Rhodobacteraceae (~16%), and Marinomonadaceae (~6%), and was similar to the first sample of the 0.1 day^−1^ dilution treatment for both MC1 and MC2 at 0.8 days, except for Alteromonadaceae and Clade III (SAR11), both of which increased in relative abundance, and Saprospiraceae, which decreased in relative abundance, compared to the Siders Pond sample. There were two samples from MC1 that did not successfully sequence (at 6.8 and 9.5 days) for the 0.1 day^−1^ dilution rate, but they did sequence in MC2 and were comprised primarily of Rhodobacteraceae (~20%–25%) and Methylophilaceae (genera *Methyloternera* ~13%–16% and *Methylophilus* ~4%–7%). Time point 10.5 days in MC1 looked like MC2 sample at 9.5 days, except for an increase in Burkholderiaceae (~8%). While MC1 at 15.5 days was similar to MC2 at 10.5 days, which was dominated by Flavobacteriaceae (~51%–55%), the two chemostats diverged in community composition from that point onward, even though external conditions were the same for both microcosms (Fig. 3).

**Fig 4:**
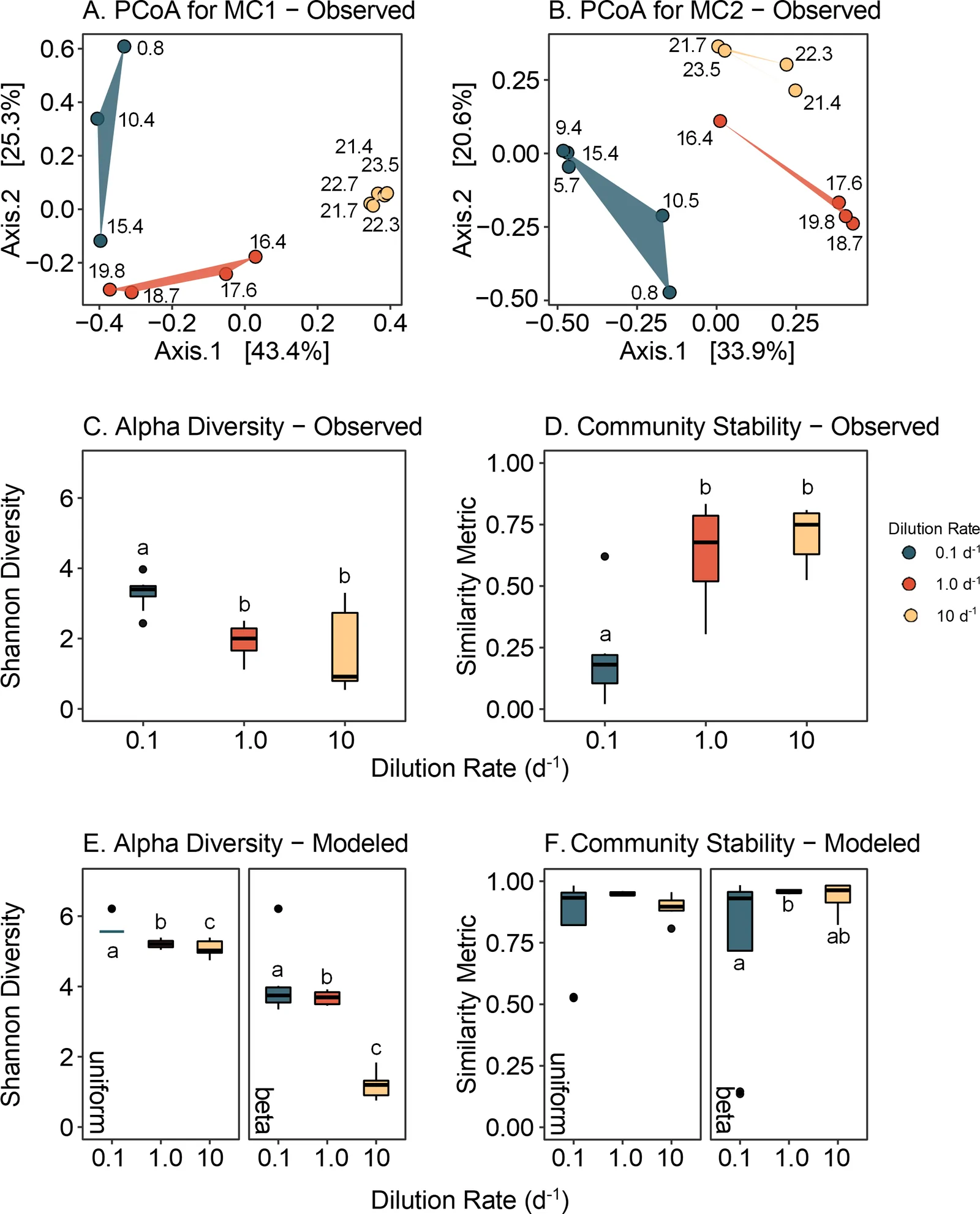
Ordination of principal coordinate analysis based on Bray-Curtis similarity among samples for (**A**) MC1 and (**B**) MC2, where numbers represent time (in days) after the start of the experiment. Boxplots represent (**C**) alpha diversity based on Shannon Index and (**D**) community stability based on Bray-Curtis similarity between consecutive time points separated by MC, as well as (**E**) alpha diversity and (**F**) community stability for modeled data from the uniform (left) and beta (right) distribution simulation. Groups that do not share the same letter are significantly different according to a Tukey’s HSD multiple comparison test. Color indicates dilution rate (blue, 0.1 day^−1^; red, 1.0 day^−1^; yellow, 10 day^−1^).

There were clear successional patterns in MC1 throughout the 1 day^−1^ dilution rate. For the first two time points, Saccharospirillaceae (ASV 1; genus *Oceanobacter*), which made up ~42% of the population on 16.5 days and ~35% on 16.6 days, and Flavobacteriaceae, which made up ~24% on 16.5 days and ~21% on 17.6 days, were the most dominant families. Opitutaceae increased from ~3% on 16.5 days up to ~60% on 19.8 days, with Saccharospirillaceae and Flavobacteraceae decreasing to ~6% and 11%, respectively. While both Rhizobiaceae and Paracaedicateraceae were present in 16.5 and 17.6 days, they were effectively absent (<1%) by 18.6 days (Fig. 3A). We observed a similar successional pattern comprised of different ASVs in MC2 throughout the 1 day^−1^ dilution rate, where Methylophilaceae was the most dominant family (~32%) on 16.5 days but was quickly displaced by Cellvibionaceae (~48%) and Kordiimonadaceae (~31%); Cellvibrionaceae then increased throughout the remainder of the 1 day^−1^ dilution rate sampling time points, making up ~80% and 68% on 18.8 and 19.8 days, respectively (Fig. 3B; Table S1). Overall, there was a decrease in alpha diversity as the dilution rate increased from 0.1 to 1 day^−1^ (Fig. 4C).

Microbial community composition exhibited very different patterns once dilution rate increased to 10 day^−1^, and these patterns differed between chemostats. In MC1, the same ASV belonging to the Saccharospirillaceae family (ASV 1; genus *Oceanobacter*) increased from ~82.5% on 21.5 days to 90.5% on 22.8 days, where it then decreased to 85% on 23.5 days due to an increase in a member of the Spirosomaceae family (ASV 7; ~8%). The only other relatively dominant ASV belonged to the Methylophilaceae family (ASV 3), which also appeared for a short time in the lowest dilution rate (10.5 and 15.5 days) and re-appeared near the end of the 1 day^−1^ dilution rate (Fig. 3A; 18.8 and 19.8 days). Cellvibrionaceae (ASV 2; ~27.5%) continued to be the most dominant ASV in MC2 at the start of the 10 day^−1^ dilution rate (21.5 days), in addition to Spirosomaceae (ASV 7; ~14%), Chitinophagaceae (ASV 8; ~10%), Comamonadaceae (ASV 17; ~9%), Methylophilaceae (ASVs 3 and 6; ~8% and 5%), and Caulobacteraceae (ASV 14; ~3%). Throughout the final three time points, Chitinophagaceae and Cellvibrionaceae demonstrated both increasing and decreasing patterns, while Methylophilaceae, composed of ASVs 3 (*Methylophilus*), ASV 6 (*Methylotenera*), and ASV 12 (*Methylophilus*), consistently increased to become the most dominant family (~52%; Fig. 3B; Table S1). It appears that MC2 experienced some kind of metabolic instability, as large oscillations in pO_2_, DO, CO_2_, and pH are evident during this phase (Fig. 2A through D, *t* > 21 days, dashed lines), which may explain the higher diversity in MC2 at the 10 day^−1^ dilution rate because the chemostat was not near steady-state operation. While there is a decrease in the alpha diversity median as dilution rate increased from 1 to 10 day^−1^, it is not statistically significant (Fig. 4C).

Mean cell abundance was 5.45 × 10^6^ ± 5.21 × 10^6^, 4.09 ± 10^6^ ± 1.88 × 10^6^, and 1.97 × 10^6^ ± 3.44 × 10^6^ cells L^−1^ for 0.1, 1, and 10 day^−1^ dilution rates, respectively (Fig. S1). According to a model that included dilution rate as the only fixed effect, cell counts decreased from 1 to 10 day^−1^ (*P* = 0.02), with no significant difference between 0.1 and 1.0 day^−1^ dilution rates. Model selection for beta dispersion supported the null model, indicating no evidence for statistical differences among dilution rates; however, average distance to group centroids showed decreasing trends ranging from 0.53, 0.31, 0.14 (MC1) and 0.45, 0.34, 0.26 (MC2) for dilution rates 0.1, 1, and 10 day^−1^, respectively (Fig. 4A and B). Alpha diversity, represented here by Shannon indices, significantly decreased as dilution rate increased from 0.1 to 1.0 day^−1^ (Table S3; *P* = 0.005) and then remained the same from 1.0 to 10 day^−1^ (Fig. 4C). In contrast, community stability (i.e., community similarity between consecutive time points) increased significantly between the 0.1 and 1.0 day^−1^ dilution rate (Table S3; *P* = 0.003), but not between 1.0 and 10 day^−1^ (Fig. 4D).

### Simulations

In the simulation run where the growth efficiency traits ($\varepsilon_{i}$) were drawn from a uniform probability distribution (Fig. 1A), ASV diversity was high at all time points that corresponded to MC sample times, and alpha diversity averaged over each dilution rate (Fig. 4E, uniform) was higher than observed for all dilution rates (Fig. 4C). Statistical model selection supported a “dilution × ‘day within phase’” interaction, indicating dilution-specific temporal trends in alpha diversity. There was a small but significant decrease in diversity as dilution rate increased (*P* < 0.001), while the rate of change through time at the 10 day^−1^ dilution rate was significantly steeper compared to both 0.1 and 1.0 day^−1^ (*P* < 0.001), which did not differ from each other. In the second scenario, where $\varepsilon_{i}$ was drawn from a beta probability distribution (Fig. 1B), diversity was greatly reduced for all three dilution rates (Fig. 4E, beta) and comparable in value to those observed (Fig. 4C). There was a significant loss of diversity at the highest dilution rate (*P* < 0.001), which is also evident in comparison with the two timeseries plots (Fig. S2), although diversity also decreased significantly between the 0.1 and 1.0 day^−1^ dilution rates (*P* < 0.001). Both simulations show that populations were more stable at each dilution rate as compared to the observed data, especially at *D* = 0.1 day^−1^ (Fig. 4F vs D). For the uniform probability distribution, AIC statistical analysis supported a null model, indicating no evidence for differences among dilution rates. In contrast, an additive model indicated that stability increased between *D* = 0.1 and 1.0 day^−1^ (*P* = 0.042), with no statistically significant differences between the higher dilution rates (Fig. 4F). The total bacterial biomass was higher for the uniform distribution compared to the beta distribution (Fig. S3), but both simulations show increasing biomass as dilution rate increases, while observed cell counts remained flat or decreased slightly with dilution rate (Fig. S1). However, we did not measure cell volume, so modeled biomass cannot be compared to cell numbers, because cellular carbon quota can change by a factor of 5 or more depending on growth conditions (61). In general, there is close agreement between modeled and observed oxygen concentrations in both the headspace (pO_2_) and medium (DO) for both simulations, but the beta distribution shows higher pO_2_ and DO in the *D* = 10 day^−1^ phase (Fig. S4). However, the significant fluctuations in headspace pO_2_ and media DO in MC2 (Fig. S4, gray lines) at the highest dilution rate were not captured by either simulation, which indicates instability in community metabolism in MC2 at *D* = 10 day^−1^. This instability is also evident in the CO_2_ observations (Fig. 2A, dashed line).

## DISCUSSION

In this study, we examined how the specific growth rate trait, $\mu (\varepsilon_{i})$, is distributed in a natural microbial community by using chemostats run at monotonically increasing dilution rates, D, that remove microbes whose specific growth rates are less than D. The experimental results are compared to a trait-based model where maximum specific growth rates of the population, set by $\varepsilon_{i}$, are randomly drawn from either a uniform distribution or one skewed toward slow-growing oligotrophs.

**Fig 5:**
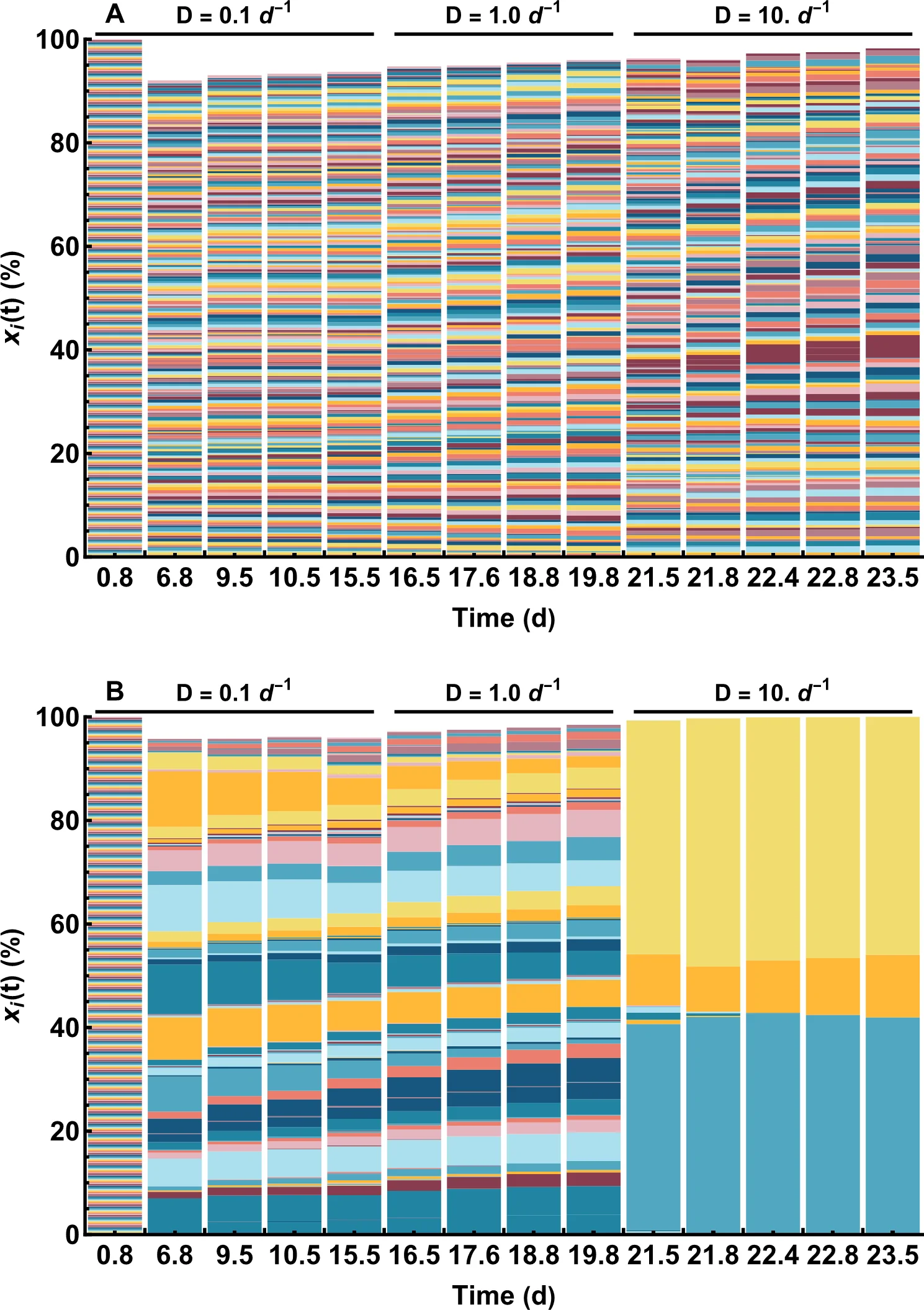
Relative abundance (%) of simulated ASVs at selected times for $\varepsilon_{i}$ drawn from (**A**) uniform and (**B**) beta probability distributions for (see Fig. 1). Only ASVs whose relative abundance equals or exceeds 0.1% are shown. Dilution rate at the time of model query is shown at the top. These plots are from the sample 1 runs.

Experimentally, we found alpha diversity was highest at the 0.1 day^−1^ dilution rate and decreased with increasing dilution rate (Fig. 4C). Simulation results at the lowest dilution rate for the uniform and beta distributions of $\varepsilon_{i}$ also show the highest alpha diversity (Fig. 4E and 5, times 0.8 to 15.5 days), but alpha diversity using the beta distribution model for $\varepsilon_{i}$ is closer in value to that observed for the chemostats at all dilution rates (Fig. 4C vs E). Alpha diversity for the model based on the uniform distribution is too high at all dilution rates, especially at the highest dilution rate.

We argue that this general decrease in diversity is rooted in the “boundary of life” concept (62), where species diversity decreases as the boundaries where life can exist are approached—in this case, maximum specific growth rate. When we draw $\varepsilon_{i}$ from a uniform distribution, community diversity remained high even at the 10 day^−1^ dilution rate, which is inconsistent with our observations. To persist in the chemostat, a bacterium’s net specific growth rate (i.e., accounting for predation) must equal or exceed the dilution rate to avoid being washed out of the system. As dilution rate increases, only those bacteria with high enough net specific growth rates will persist. Results show that only a small proportion of the natural pond community could grow fast enough to persist under the highest dilution rate imposed, which is consistent with Weisssm et al. (63), who found a bias for extremely slow-growing organisms based on codon usage patterns. In a resource competition context, the functional traits most relevant are maximum specific growth rate and substrate affinity, as these two strategies are among the most important mechanistic drivers of biodiversity (64). However, there is a trade-off between substrate affinity (or low minimum resource requirement) and maximum specific growth rate (25, 65). This trade-off between gleaner and opportunist bacteria (32) is not binary but is a continuum between the two extremes, which we represented with the trait variable $\varepsilon_{i}$ in our adaptive Monod kinetics model (equation 1) that varies between 0 (gleaner/oligotroph) and 1 (opportunist/copiotroph). This skewed continuum of growth strategies likely shaped the changes in observed microbial diversity as a function of dilution rate, where resource competition changed from a high-diversity gleaner-type community at the 0.1 day^−1^ dilution rate to a low-diversity opportunist-like community at the 10 day^−1^ dilution rate, since resource concentrations increase with increasing dilution rate (33).

As noted by Locey and Lennon (21), there have been relatively few studies relating residence time (τ; the inverse of dilution rate, 1/D) to biodiversity and related metrics of community assembly. Ratzke et al. (66) examined how resource concentration alters microbial community diversity and stability, but under batch operating conditions, so not comparable to our study. Locey and Lennon (21) used a trait-based model study that drew from a uniform distribution and found that species richness decreased with increasing dilution rate as we did, but they also found at dilution rates lower than ~10^−3^ day^−1^, richness began to decrease, thereby creating a unimodal distribution in richness as a function of D. Mansfeldt et al. (67) investigated the impact of microbial residence time on community composition (16S rRNA-based) and function (RNAseq-based) in laboratory batch reactors treating wastewater over residence times varying from 1 to 15 days (or dilution rates from 0.066 to 1 day^−1^). Similar to our study, both the experimental results and the model they developed showed a decrease in Shannon diversity and richness as dilution rate increased. Both the Locey and Lennon (21) and Mansfeldt et al. (67) studies differ from ours in objectives and design, making them difficult to compare, but all suggest that traits, such as the maximum specific growth rate, are not uniformly distributed in a community but are instead skewed toward oligotrophs.

The other metric examined in our study was community stability, which reflects how quickly community composition changes over time. Experimentally, we found community stability increased (Fig. 4D) and distance-to-group centroid decreased between sampling time points with increasing dilution rate (Fig. 4A and B). One explanation for this increase in community stability is the loss of diversity at higher dilution rates. The high diversity at low dilution rate might lead to considerable functional redundancy. There are likely alternate subcommunity assemblies that produce similar metabolic functions, and these subcommunities can replace one another over time, leading to a continuous succession of ASVs (68). At high dilution rates, community diversity is greatly diminished; consequently, there is simply a lack of ASVs that can contribute to community succession. Our model, however, does not accurately capture this observed change in community stability with dilution rate, but instead shows high stability across all dilution rates (Fig. 4F), regardless of the distribution $\varepsilon_{i}$ is chosen from (uniform or beta). This indicates our model is missing factors that cause rapid turnover of the community at low dilution rates, which might be explained by fluctuations between alternate stable states (69), cross-feeding (37, 70–72), or more targeted kill-the-winner type predation or displacement (68, 73, 74) that our simple model did not include. Although model complexity could be increased to explore causes of community instability, measurements of protist and virus concentrations in combination with metabolomics would be needed for model verification, which were beyond the scope of our study. The mismatch in community temporal stability between the experiment and model is common; we suggest this is a useful research area to explore in future studies.

### Conclusion

We conducted a chemostat experiment using a natural pond microbial community in conjunction with an adaptive Monod trait-based model to examine how community diversity changes as a function of specific growth rate dictated by the chemostat dilution (or turnover) rate. We hypothesized that the number of bacterial species (alpha diversity) would decrease under increasing dilution rates because natural microbial communities are skewed toward bacteria with oligotrophic rather than copiotrophic growth characteristics. The hypothesis was confirmed experimentally, as we observed a decrease in alpha diversity with increasing dilution rate. Observations also showed rapid community turnover at low dilution rate, which we have observed in other chemostat experiments (37), but community stability increased at high dilution rates, likely due to the lack of diversity. Model results show that when the growth rate trait variable is drawn from a beta distribution that is skewed toward oligotrophic growth strategies, alpha diversity decreases with increasing dilution rate as observed in the chemostats, supporting the skewed growth rates hypothesis. When the trait variable is drawn from a uniform distribution, communities retain high diversity as the upper growth rate boundary is approached, which is inconsistent with our observations. However, neither model distribution captured the increase in community stability with increasing dilution rate, which may be due to the lack of explicitly including consumers or cross-feeding in the model. Our results also illustrate the importance of trait-based model initialization for proper prediction of biodiversity dynamics in microbial communities.

## Data Availability

All raw sequences have been placed in the NCBI Sequence Read Archive (SRA) under BioProject ID PRJNA1103251 (accession numbers: SRX24340248–SRX24340273).

## SUPPLEMENTAL MATERIAL

File S1 spectrum.02193-26-s0001.pdf Mathematica code (Trait-based model code) used in manuscript.

Supplemental figures spectrum.02193-26-s0002.pdf Fig. S1 to S4.

Table S1 spectrum.02193-26-s0003.csv Table S1 of ASVs.

Table S2 spectrum.02193-26-s0004.xlsx Table S2 for Fig. 2 statistics.

Table S3 spectrum.02193-26-s0005.xlsx Table S3 for Fig. 4 statistics.

ASM does not own the copyrights to Supplemental Material that may be linked to, or accessed through, an article. The authors have granted ASM a non-exclusive, world-wide license to publish the Supplemental Material files. Please contact the corresponding author directly for reuse.
