# Supplementary material for: Growth characteristics of natural microbial populations are skewed toward bacteria with low specific growth rates
Source: Microbiol Spectr. 2026 Aug 4;14(9):e02193-26. doi: 10.1128/spectrum.02193-26 (PMC13532248; doi:10.1128/spectrum.02193-26)
Supplement: Supplementary file 1 [file spectrum.02193-26-s0001.pdf]

Trait-Based Model

This model examines how the initial distribution of traits in a microbial population impacts diversity in a chemostat as dilution rate increases. Two scenarios are examined, one where the trait governing bacteria specific growth rate, ϵ_i is uniformly distributed, such that there are as many bacteria that have a specific growth rate near the upper growth rate limit as for any other specific growth rate. The other scenario examines our hypothesis that as you approach the upper specific growth rate limit for bacteria, there are many fewer ASVs capable of growing at that boundary. We randomly select ϵ_i from a beta probability distribution to explore the second scenario. Experimental details and data the model simulates can be found here http://ecosystems.mbl.edu/MEP-FoodWeb/Experiments/Exp1/index.html
22-Jul-2024
This Version 2 includes a quadratic mortality term weighted by relative ASV abundance for the bacterial balance equation.

Notebook setup

In[55]:=
```
$Version
```
Out[55]=
```
14.3.0 for Microsoft Windows (64-bit) (July 8, 2025)
```

Set the working directory to where this notebook is saved (it is assumed the github repo directories have not been changed)

In[1]:=
```
SetDirectory[NotebookDirectory[]];
```

Turn off plot highlighting as it slows the front end down when plots have a lot of elements

In[2]:=
```
Charting`$InteractiveHighlighting = False;
SetOptions[Plot, PlotHighlighting → None];
SetOptions[ListPlot, PlotHighlighting → None];
```

Plot sometimes needs more recursion than the default, so set it higher here

In[5]:=
```
$RecursionLimit = 2000;
```

To decrease file size, hold Alt then click on an output cell, then from the menu select "Cell → Convert To → Bitmap". (note, Alt+click selects all output cells). Also, graph outputs can be wrapped with Rasterize[], which does the same thing.

Experimental On-Line Data

Dissolved oxygen, DO (μM), and partial pressure of oxygen in the chemostat gas headspace (%) from MC1 and MC2 during the course of the experiment.

Import Exp1.csv that has the O2, CO2, DO and pH for the two chemostats. From Exp1.csv, just use the

O2 data, as the DO can be obtained from the Exp1_DOpH.csv file, which is of higher resolution because it did not rely on an A2D converted. In both cases drop the header from the csv data.

In[6]:=
```
o2Data = {Drop[Import["../Data/Exp1.csv"], 1]⟦All, {1, 2}⟧,
    Drop[Import["../Data/Exp1.csv"], 1]⟦All, {6, 7}⟧};
```

In[7]:=
```
Dimensions[o2Data]
```

Out[7]=
```
{2, 639, 2}
```

Plot the pO2 data

In[8]:=
```
ListLinePlot[o2Data, PlotRange → All]
```

Out[8]=

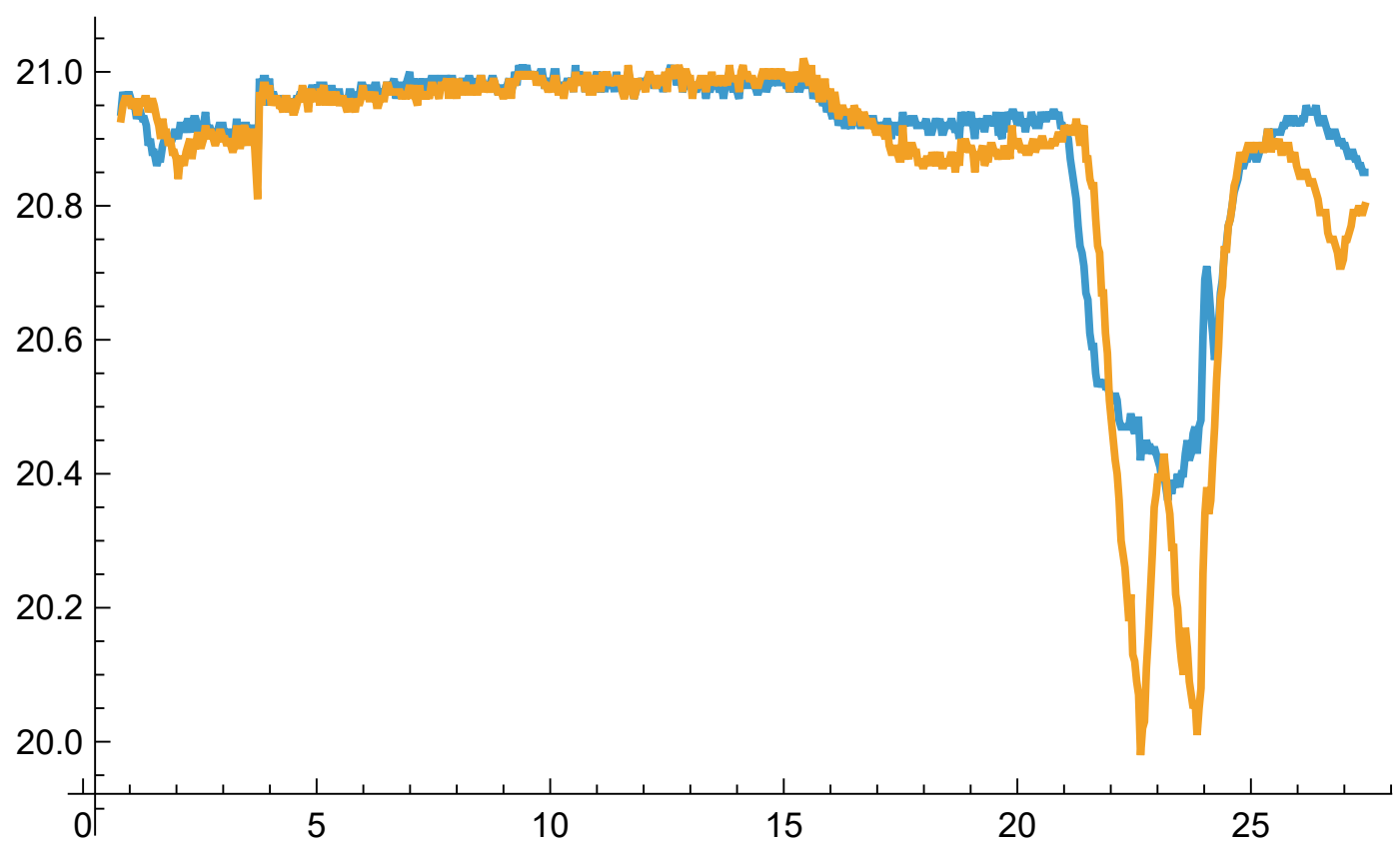

In[9]:=
```
doData = {Drop[Import["../Data/Exp1_DOpH.csv"], 1]⟦All, {3, 4}⟧,
    Drop[Import["../Data/Exp1_DOpH.csv"], 1]⟦All, {7, 8}⟧};
```

Now convert mg/L to uM O2

In[10]:=
```
doData = {doData⟦1⟧ /. {x_, y_} → {x, 1000 y / 32}, doData⟦2⟧ /. {x_, y_} → {x, 1000 y / 32}};
```

Plot the DO data

In[11]:=
```
ListLinePlot[doData, PlotRange → All]
```

Out[11]=

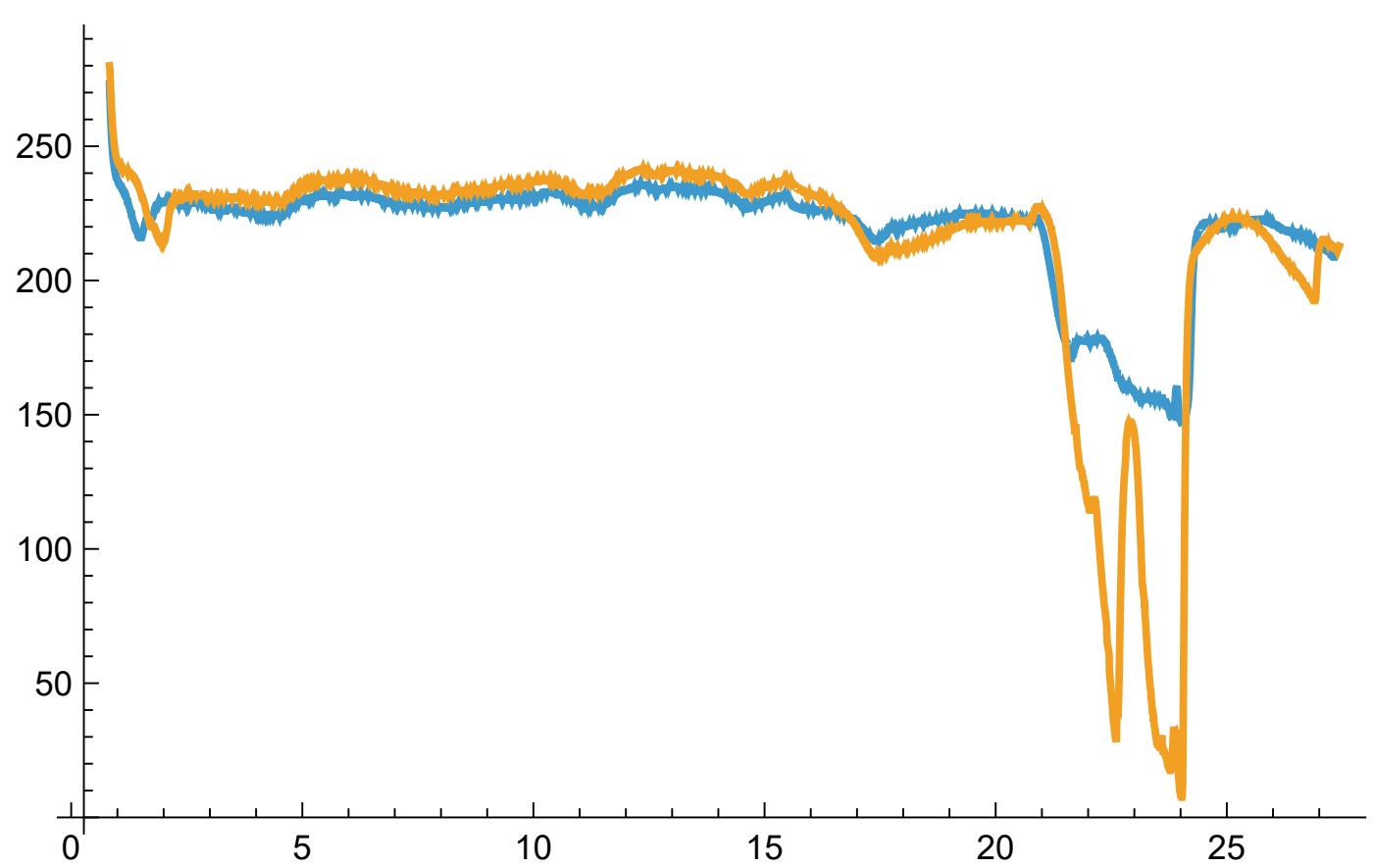

Dissolved Oxygen Data for k_L estimation

This data is used to determine the liquid-side oxygen mass transfer velocity, k_L (see below). DO data was collected following sparging of the chemostat with N_2 gas. After sparging with N2, the gas flow was

changed to air, and the chemostat headspace was quickly equilibrated with air using a small fan for a short time so that the gas overlying the water would be at atmospheric pressures (i.e., fixed boundary condition). Import the DO data:

In[12]:=
```
o2DataN2 = Import["../Data/DO_N2toAir.dat"];
```

Plot the DO observations.

In[13]:=
```
ListPlot[o2DataN2, FrameLabel → {"Time (d)", "DO (μM)"},
 Frame → {{True, False}, {True, False}}, BaseStyle → {FontSize → 15, FontWeight → Bold}]
```

Out[13]=

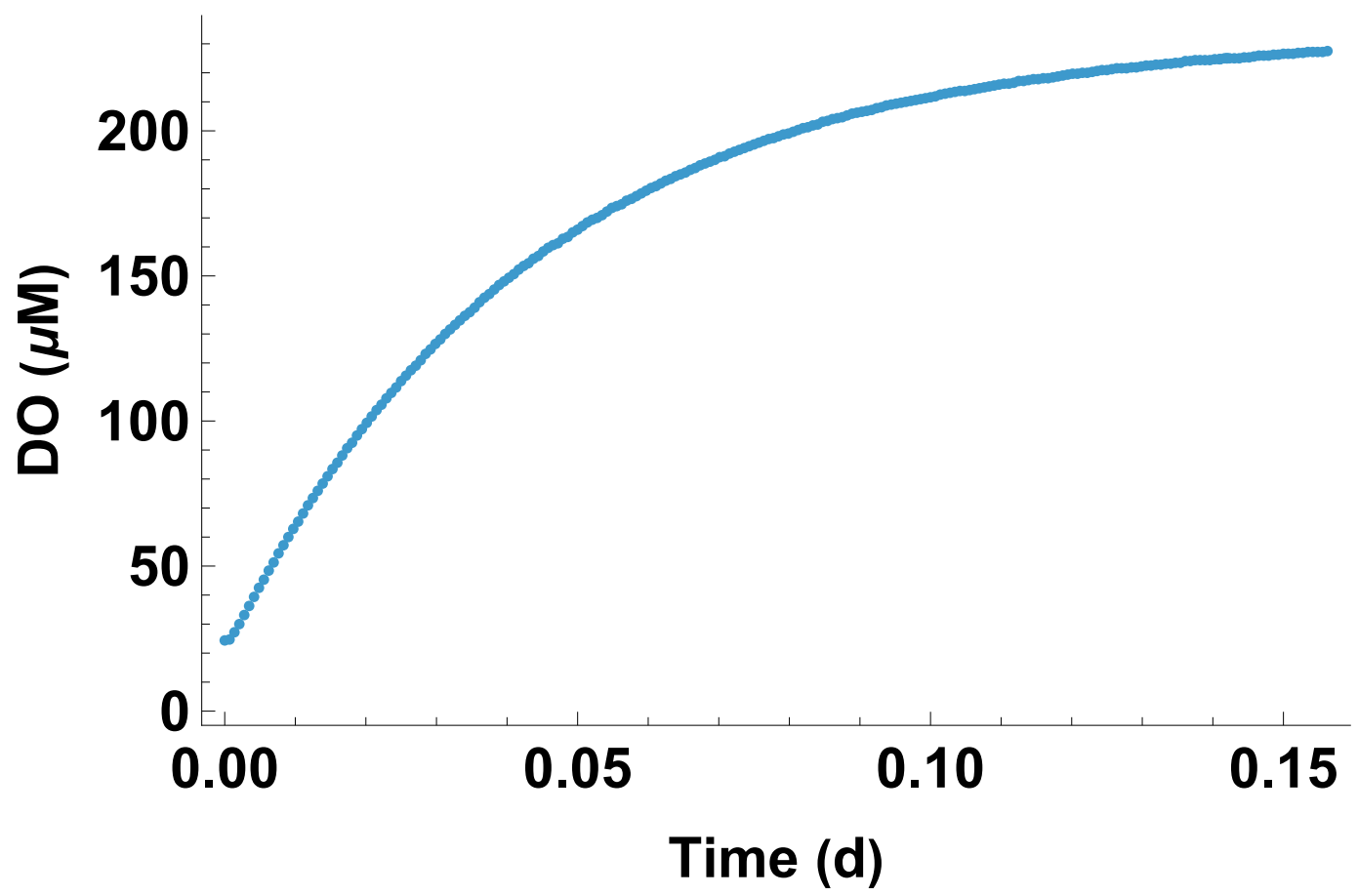

Estimate k_L *a* from data for transition from N_2 to Air

Preliminary model testing (no need to evaluate this subsection)

Estimate kL based on DO observations Only

Since we quickly equilibrated the headspace with air after driving the system anoxic with N_2, the governing ODE only needs to track dissolved oxygen (DO), as the gas phase remains constant as it is in equilibrium with air. Here is the governing equation for DO.

In[26]:=
```
odesDO[input_List] := {
   c'[t] == (fL (cF - c[t]) + kL a (p[t] h - c[t])) / vL,
   c[0] == cF} /. input
```

The Henry's constant as a function of temperature (K) for oxygen (see https://henrys-law.org/henry/casrn/7782-44-7) in μM atm^{-1} is:

In[27]:=
```
hO2[tk_] := 0.00131722 e^(1700 (1/tk - 1/298.15)) 10^6
```

$$hO2[tk] := 0.00131722\, e^{\left(1700\left(\frac{1}{tk}-\frac{1}{298.15}\right)\right)}\, 10^6$$

Below solves the ODE and plots the solution versus the DO observations. The only parameters adjusted here are the oxygen piston velocity, kL (m d^{-1}), which was the objective of the experiment and the oxygen concentration in the atmosphere, p[t] (atm). Note, it should not have been necessary to change p[t], as it should be 0.21 atm; however, our DO probe may not be perfectly calibrated (or per-

haps the headspace still contained some of the N2 gas used for purging) and is reading a bit low; consequently, the O_2 concentration was decreased to 0.177 atm in order to get a good fit. **The value found for the piston velocity is 1.65 m d^{-1}, which is pretty close to the estimated value of 1.3**.

In[28]:=
```
Show[
 Plot[Evaluate[c[t] /. NDSolve[odesDO[{fL → 0, cF → 24.7, kL → 1.65, a → π 0.114^2, h → hO2[298],
       vL → 3 / 1000, p[t] → 0.177}], {c[t]}, {t, 0, 10}][[1]]], {t, 0, 0.2},
  PlotRange → All, PlotStyle → Red, FrameLabel → {"Time (d)", "DO (μM)"},
  Frame → {{True, False}, {True, False}}, PlotLegends → {"Model"}],
 ListPlot[o2DataN2, PlotStyle → Blue, PlotLegends → {"Data"}],
 BaseStyle → {FontSize → 15, FontWeight → Bold}]
```

Out[28]=

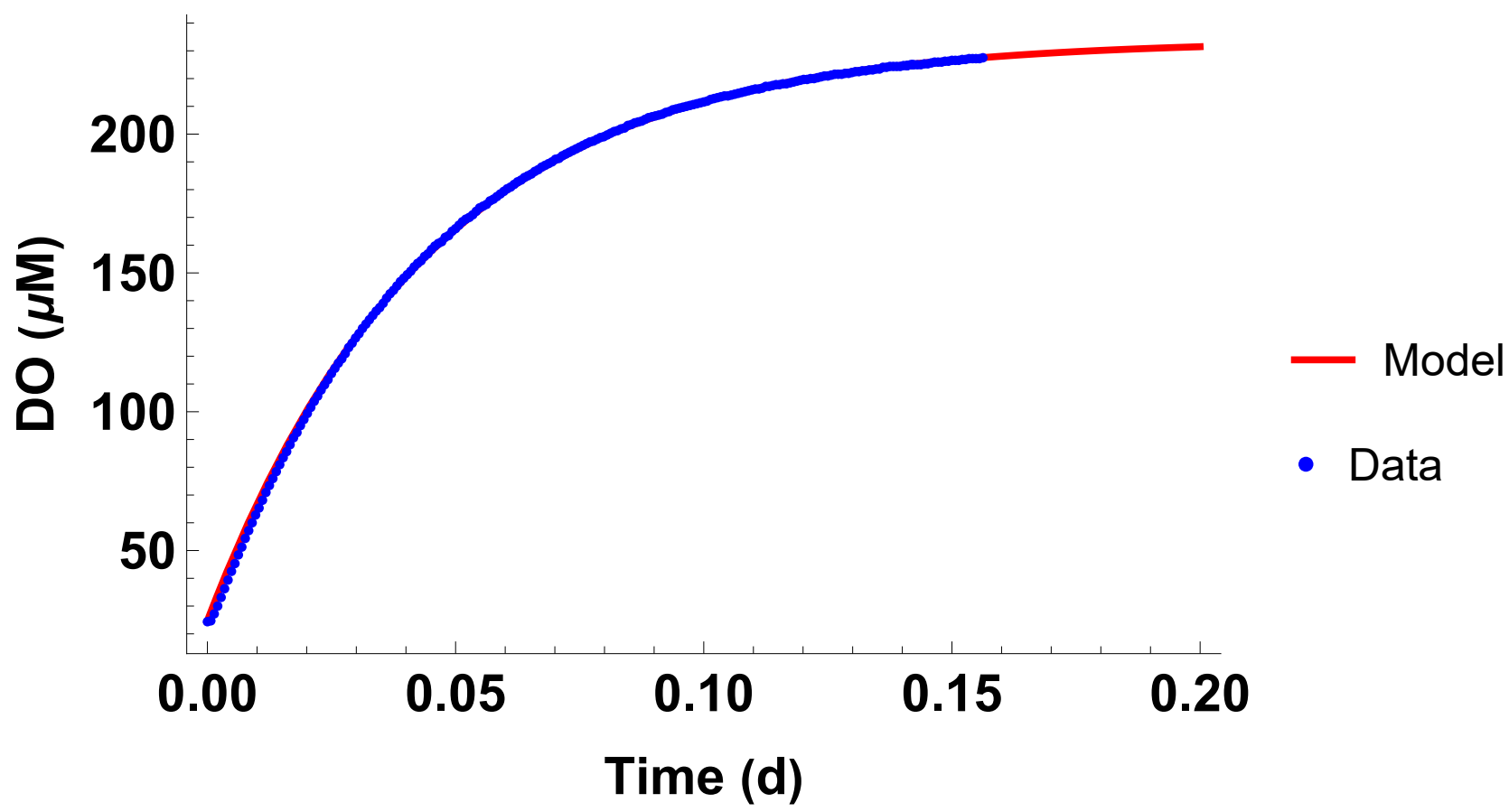

Governing Equations for Trait-Based Model

Adaptive Monod Kinetics

The adaptive Monod equation, written as the specific substrate uptake rate, v, not the specific growth rate; that is, $\mu = \epsilon v$. Standard parameters are used here. This function chooses along a continuum from gleaners ($\epsilon \to 0$) to opportunists ($\epsilon \to 0.7$). Note, as ϵ exceeds 0.77, uptake rate decreases due to loss of thermodynamic drive (i.e., growth reaction approaching equilibrium). Also see Vallino & Huber (2018) doi: 10.3389/fenvs.2018.001000

In[29]:= $$v[s\_, \epsilon\_] := vM\, \epsilon^2 \frac{s}{s + kM\, \epsilon^4} \left(1 - \epsilon^2\right)$$

The adaptive Monod equation gives the following family of curves for different values of ϵ for the standard values of vM (350 d^{-1}) and kM (5000 μM).

In[30]:=
```
Plot[Evaluate[Table[ Tooltip[ϵ v[s, ϵ], ϵ], {ϵ, 0.1, 1, 0.15}] /. {vM → 350, kM → 5000}],
 {s, 0, 5000}, PlotStyle → {{Red, Thick}, {Blue, Thick}, {Green, Thick},
   {Purple, Thick}, {Orange, Thick}, {Gray, Thick}, {Cyan, Thick}},
 Frame → True, FrameStyle → {{Black, White}, {Black, White}},
 FrameLabel → {"[S]  (μM)", "Specific Growth Rate  (d^-1)"},
 LabelStyle → {Bold, FontFamily → "Arial", FontSize → 16}]
```

Out[30]=

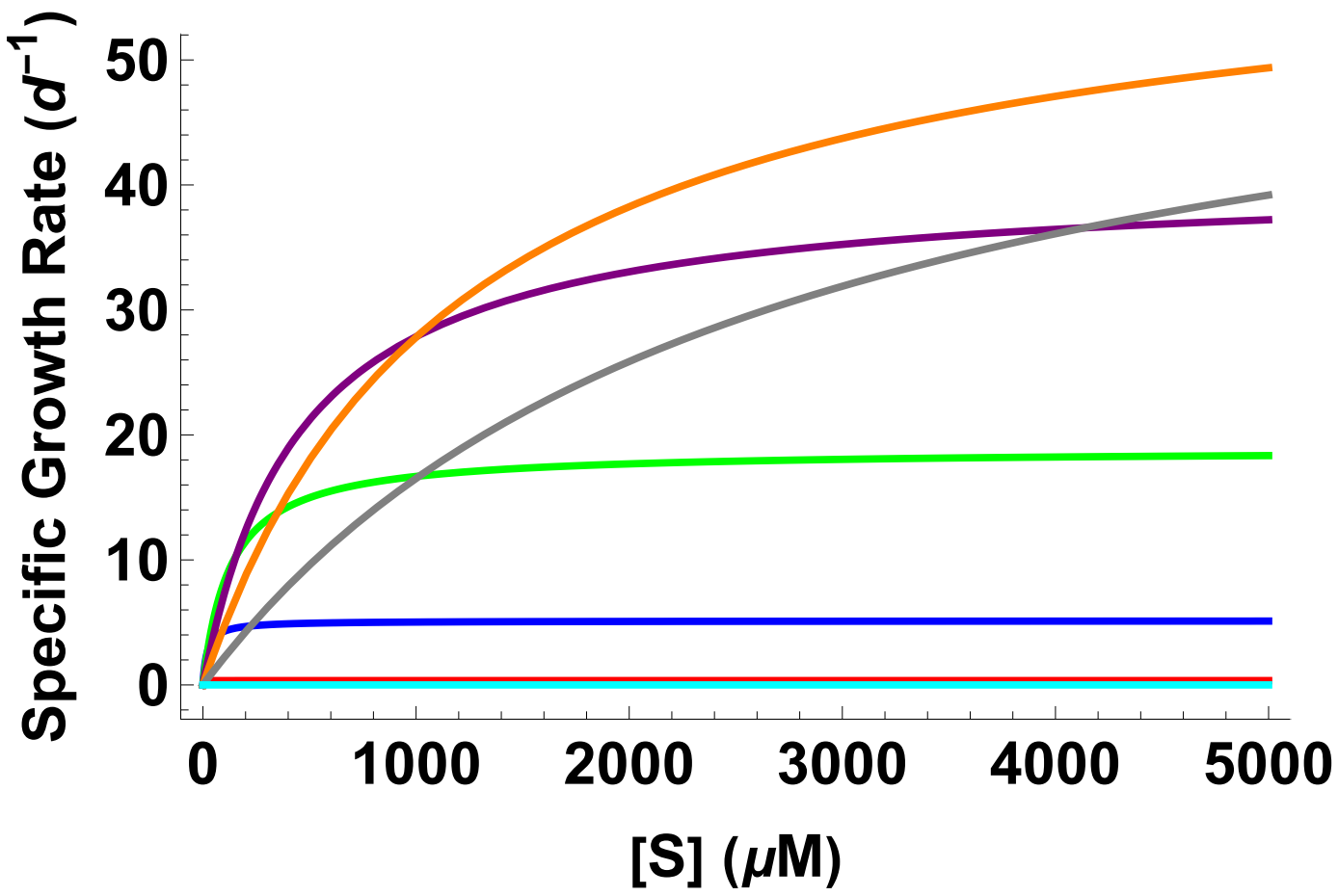

redefine the function with the standard values for the parameters set

In[31]:=
```
(* v[s_,ϵ_]:= 350 ϵ^2 s/(s+5000 ϵ^4) (1-ϵ^2) *)
```

Saturated Oxygen in saline water

This function, satO2, returns the oxygen concentration (in μM) for a given temperature (K) and salinity (PSU) in equilibrium with air in seawater . Reference Weiss1970 : (in deep - sea research, 17 : 721 - 735) . However, the modified version here, hO2, allows for different partial pressures of oxygen, and changes the temperature unit to K .

In[32]:=
```
satO2[tK_, s_] := Module[{a1 = -173.4292, a2 = 249.6339, a3 = 143.3483, a4 = -21.8492,
   b1 = -0.033096, b2 = 0.014259, b3 = -0.0017, mlToumole = 44.62, t100},
t100 = tK/100;
mlToumole Exp[a1 + a2/t100 + a3 Log[t100] + a4 t100 + s (b1 + b2 t100 + b3 t100^2)]
]
```

In[33]:=
```
satO2[293.15, 0]
```

Out[33]=

283.405

This can be used for different partial pressures of oxygen (i.e., not just atmospheric). Also, if variables are passed to satO2, then the Exp term in satO2 is simplified, but this then leads to problems because of large and small number multiplication. By ensuring the arguments are numbers, Exp will not be

simplified which keeps this problem form occurring.

In[34]:=
```
hO2[s_?NumberQ, t_?NumberQ] := satO2[t, s] / 0.20946
```

In[35]:=
```
hO2[3, 298] 0.20946
```

Out[35]=
```
253.757
```

Setup governing equations where the number of bacteria is specified and the ϵ trait is randomly selected from a specified distribution.

The function *generateODEs* generates a set of ODEs based on the length of ranSample, where the latter is a list of ϵ_i values chosen from an appropriate probability distribution (that is, ranSample is given). The initial conditions for x[i][0] is set to 1/n, where n is the number of ASVs used and x[i] biomass of ASV i and has the units of μM C. This version includes simple quadratic mortality weighted by total biomass for closure.

In[36]:=
```
generateODEs[params_List, ranSample_List] :=
 Flatten[{Table[{x[i]'[t] == 𝒟 (0 - x[i][t]) + ϵ[i] × v[s[t], ϵ[i]] × x[i][t] -
        m x[i][t]^3 / Sum[x[i][t], {i, 1, Length[ranSample]}] }, {i, 1, Length[ranSample]}],
    s'[t] == 𝒟 (s0 - s[t]) - Sum[v[s[t], ϵ[i]] × x[i][t], {i, 1, Length[ranSample]}],
    Table[x[i][0] == 1 / Length[ranSample], {i, 1, Length[ranSample]}],
    s[0] == 0,
    o2'[t] == 𝒟 (o2f - o2[t]) + kLO2 a (pO2[t] × hO2[sal, tK] - o2[t]) / vL -
      Sum[(1 - ϵ[i]) v[s[t], ϵ[i]] × x[i][t], {i, 1, Length[ranSample]}],
    pO2'[t] == (fG (pO2f - pO2[t]) + kLO2 a rGas tK (o2[t] - pO2[t] × hO2[sal, tK])) / vG,
    o2[0] == o2f,
    pO2[0] == pO2f} /. Table[ϵ[i] → ranSample[[i]], {i, Length[ranSample]}] /. params]
```

This is an older version of generateODEs that did not include quadratic mortality. This is not used in the current version and has been renamed to generateODEsOld

In[37]:=
```
generateODEsOld[params_List, ranSample_List] :=
 Flatten[{Table[{x[i]'[t] == 𝒟 (0 - x[i][t]) + ϵ[i] × v[s[t], ϵ[i]] × x[i][t]},
      {i, 1, Length[ranSample]}],
    s'[t] == 𝒟 (s0 - s[t]) - Sum[v[s[t], ϵ[i]] × x[i][t], {i, 1, Length[ranSample]}],
    Table[x[i][0] == 1 / Length[ranSample], {i, 1, Length[ranSample]}],
    s[0] == 0,
    o2'[t] == 𝒟 (o2f - o2[t]) + kLO2 a (pO2[t] × hO2[sal, tK] - o2[t]) / vL -
      Sum[(1 - ϵ[i]) v[s[t], ϵ[i]] × x[i][t], {i, 1, Length[ranSample]}],
    pO2'[t] == (fG (pO2f - pO2[t]) + kLO2 a rGas tK (o2[t] - pO2[t] × hO2[sal, tK])) / vG,
    o2[0] == o2f,
    pO2[0] == pO2f} /. Table[ϵ[i] → ranSample[[i]], {i, Length[ranSample]}] /. params]
```

Example, generate a set of ODEs for 3 ASVs with ϵ selected from a flat distribution where ϵ can vary between 0 and 0.7

In[38]:=
```
generateODEs[{}, RandomReal[{0, .7}, 3]] // TableForm
```

Out[38]//TableForm=

$$x[1]'[t] == -\mathcal{D}\, x[1][t] + \frac{0.00545406\, vM\, s[t] \times x[1][t]}{0.00100212\, kM + s[t]} - \frac{m\, x[1][t]^3}{x[1][t] + x[2][t] + x[3][t]}$$

$$x[2]'[t] == -\mathcal{D}\, x[2][t] + \frac{0.140959\, vM\, s[t] \times x[2][t]}{0.135155\, kM + s[t]} - \frac{m\, x[2][t]^3}{x[1][t] + x[2][t] + x[3][t]}$$

$$x[3]'[t] == -\mathcal{D}\, x[3][t] + \frac{0.0405919\, vM\, s[t] \times x[3][t]}{0.0167855\, kM + s[t]} - \frac{m\, x[3][t]^3}{x[1][t] + x[2][t] + x[3][t]}$$

$$s'[t] == \mathcal{D}\, (s0 - s[t]) - \frac{0.0306542\, vM\, s[t] \times x[1][t]}{0.00100212\, kM + s[t]} - \frac{0.232479\, vM\, s[t] \times x[2][t]}{0.135155\, kM + s[t]} - \frac{0.112773\, vM\, s[t] \times x[3][t]}{0.0167855\, kM + s[t]}$$

$$x[1][0] == \frac{1}{3}$$

$$x[2][0] == \frac{1}{3}$$

$$x[3][0] == \frac{1}{3}$$

$$s[0] == 0$$

$$o2'[t] == \mathcal{D}\, (o2f - o2[t]) + \frac{a\, kLO2\, (-o2[t] + hO2[sal, tK] \times pO2[t])}{vL} - \frac{0.0252001\, vM\, s[t] \times x[1][t]}{0.00100212\, kM + s[t]} - \frac{0.0915205\, vM\, s[t] \times x[2][t]}{0.135155\, kM + s[t]} - 0$$

$$pO2'[t] == \frac{fG\, (pO2f - pO2[t]) + a\, kLO2\, rGas\, tK\, (o2[t] - hO2[sal, tK] \times pO2[t])}{vG}$$

$$o2[0] == o2f$$

$$pO2[0] == pO2f$$

Define a function that numerically solves the generated set of ODEs over time from t0 to tf. Note, experimental time is based on 00:00 30-Oct-2018. The simulation “begins” when water was collected and placed into the collection container at about 17:00, or at t0 of 0.71 d.

In[39]:=
```
solveODEs[t0_, tf_, params_List, ranSample_List] :=
 NDSolve[Evaluate[generateODEs[params, ranSample]],
   Evaluate[Flatten[{Table[x[i], {i, 1, Length[ranSample]}], s, o2, pO2}]], {t, t0, tf}][[1]]
```

Define function to capture the shifts in dilution rate to match Exp1

First define a smooth and continuous step function, where the steepness of the function is given by σ. As σ approaches ∞, the step function approaches a perfect step, which produces discontinuities at the steps that we wish to avoid for numerical reasons. A little trial and error was used to figure out what a reasonable value of σ should be.

In[40]:=
$$\text{stepFcn}[t\_, tm\_, \sigma\_] := \frac{1}{1 + \text{Exp}[-\sigma\, (t - tm)]}$$

Then add these together to get the three dilution rates at the experimental step times of 0.84, 15.5 and 20.77 days.

In[41]:=
$$\text{expDil}[t\_, \sigma\_] := \text{stepFcn}[t, 0.84, \sigma] / 10 + \frac{9}{10}\, \text{stepFcn}[t, 15.5, \sigma] + 9\, \text{stepFcn}[t, 20.77, \sigma]$$

Here' s what that function looks like over 25 days.

In[42]:=
```
Plot[expDil[t, 50], {t, 0, 25}, PlotRange → All]
```
Out[42]=

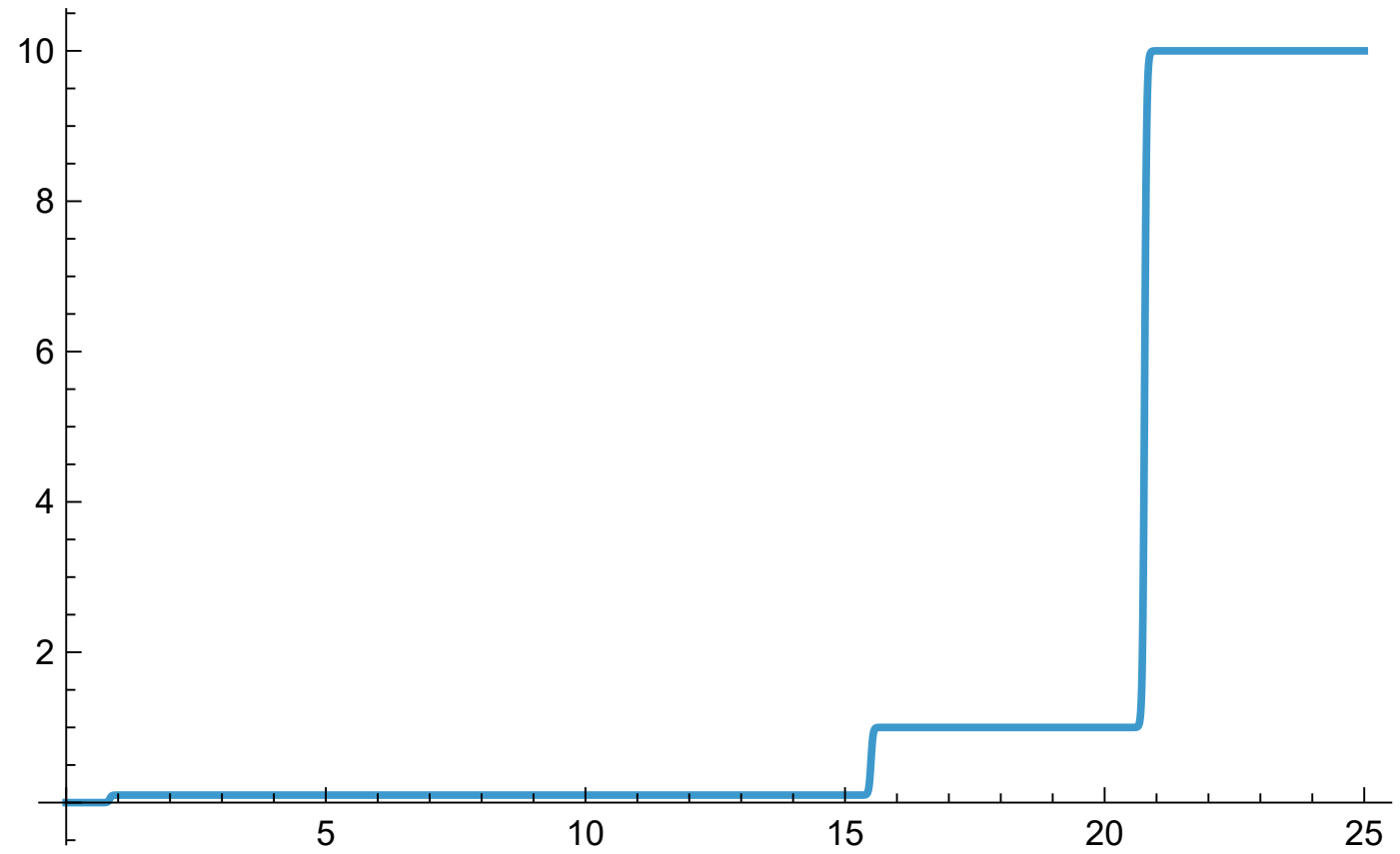

So a value of 50 for σ works well.

Function for bar charts

In[43]:=
```
xiBarChart[xiRel_List, labels_List, dBars_List, fig_] := BarChart[xiRel,
  ChartLayout → "Stacked", ChartLabels → labels, PlotRange → {{0.5, 14.5}, {0, 100}},
  LabelStyle → {Bold, FontFamily → "Arial", FontSize → 16, FontColor → Black}, ChartStyle → 24,
  FrameLabel → {"Time (d)", "x_i(t) (%)"}, Frame → {{True, False}, {True, False}},
  FrameStyle → {{Directive[Thick, Black], None}, {Directive[Thick, Black], None}},
  Epilog → {Text[Style[fig, Bold, FontFamily → "Arial", FontSize → 16], {1, 105}],
    Thick, Text[Style["D = 0.1 d^-1", Bold, 14], {(dBars[[1]] + dBars[[2]]) / 2, 105}],
    Line[{{dBars[[1]] - 0.3, 101}, {dBars[[2]] + 0.3, 101}}],
    Text[Style["D = 1.0 d^-1", Bold, 14], {(dBars[[3]] + dBars[[4]]) / 2, 105}],
    Text[Style["D = 10. d^-1", Bold, 14], {(dBars[[5]] + dBars[[6]]) / 2, 105}],
    Line[{{dBars[[3]] - 0.3, 101}, {dBars[[4]] + 0.3, 101}}],
    Line[{{dBars[[5]] - 0.3, 101}, {dBars[[6]] + 0.3, 101}}]},
  ImagePadding → {{All, All}, {All, 30}}, ImageSize → 550]
```

Uniform Distribution Solution for MS

Look at 500 ASVs with ϵ_i chosen from a uniform distribution. Note, each time this notebook is run, a new distribution will be generated. If you want to use the same distribution, make sure to save it (the one used for the manuscript is given below).

Parameters use for simulations

Notes from Exp 1 state: “Air feed rate to MC1 and MC2 reduced from 20 sccm to 10 sccm (0 °C, 1 atm) (15 : 30 2 - Nov - 2018, t = 3.65 d)”, but just run the gas feed rate constant at 10 sccm for the simulations as the 20 sccm was only run for a short time.
Also, the feed O2 was measured at 20.97 for the first 3.73 days, then either a new air tank was installed or the old one remeasured (probably the former), but that increased the O2 in the feed gas to 21.06, so just use the later for the whole simulation.

In[44]:=
```
params = {𝒟 → expDil[t, 50] (*d^-1*),
   s0 → 600, (*mmol m^-3*)
   pO2f → .2106, (*atm*)
   sal → 3, (*PSU*)
   tK → 298.15, (*K*)
   o2f → hO2[3, 298.15] .21, (*mmol m^-3*)
   kLO2 → 1.65,  (*m d^-1*)
   a → π 0.114^2, (*m^2*)
   rGas → 0.082057338 × 10^-6, (*atm m^3 mmol^-1 K^-1*)
   vL → 3 / 1000, (*m^3*)
   vG → 4.74 / 1000, (*m^3*)
   fG → 15.72 / 1000, (*m^3 d^-1*)
   vM → 350,  (* d^-1 *)
   kM → 5000,  (* mmol m^3 *)
   m → 0.1 (*m^3 mmol^-1 d^-1*)
  };
```

In[∘]:=
```
uniform1 = RandomReal[{0, .7}, 500];
```

This is the uniform distribution used in the manuscript:

In[45]:=
```
uniform1 = {0.2065482419257525`, 0.4172611296271571`, 0.35183185854095655`,
   0.4366451828429523`, 0.3560395316384306`, 0.42449820124996496`, 0.6809594877225937`,
   0.38842794341524045`, 0.5847126988356506`, 0.12897170837409266`, 0.6597463035814124`,
   0.4014891766117026`, 0.23870072093247252`, 0.691996463899059`, 0.014317448635199903`,
   0.652940269807988`, 0.2984156979074397`, 0.4664411076580335`, 0.392198378063897l`,
   0.6771372386680696`, 0.17839566115559657`, 0.35206258907344345`, 0.5171330447617475`,
   0.41650325004106237`, 0.29624707718079657`, 0.45497483718317033`, 0.392919401893258`,
   0.3732743676998975`, 0.08501767658279824`, 0.598904272697989`, 0.23202487559314888`,
   0.21349869826588597`, 0.667393800437001`, 0.3067727707769514`, 0.6166236165635428`,
   0.6314711452881445`, 0.3269875087285028`, 0.05652320530437438`, 0.2386818464093351`,
   0.23618223486855672`, 0.42154032217211923`, 0.5062047536523835`, 0.23721474795391917`,
   0.13196347011561338`, 0.22088640475114218`, 0.699243864658845`, 0.3116951761916804`,
   0.431017207214462`, 0.3002163870388015`, 0.250338211615442`, 0.29806288030076`,
   0.32310060134129626`, 0.33340928184110874`, 0.44892330441803985`, 0.5570765748832738`,
   0.6653657004231293`, 0.5762303034727962`, 0.4839664379219819`, 0.32493975268700726`,
   0.521333415767236 9`, 0.5152970522232245`, 0.6598936468281722`, 0.538656435600180 2`,
   0.5232076771678291`, 0.544446484685907`, 0.27026510317930574`, 0.1833846234772416`,
   0.573610429662316 6`, 0.6006094731759439`, 0.2672580718518641`, 0.04709393805897244`,
   0.05941964602570604`, 0.5622078444826373`, 0.2274815658721403`, 0.500709302515635 5`,
   0.25133611580942705`, 0.6105546481148267`, 0.1979532672525618`, 0.05000807220941472`,
   0.55206682323358 7`, 0.2590003935727958`, 0.237457443753463`, 0.016898439574316693`,
   0.572112610750157 3`, 0.06465811590082526`, 0.6363583574943912`, 0.19032981350339662`,
   0.559154482555692`, 0.26915324214085745`, 0.6896679493978137`, 0.632896679704996`,
   0.5150649385804618`, 0.43121230981447023`, 0.4059060326582662`, 0.6318075104634733`,
   0.265150288983064 6`, 0.41725198184995405`, 0.6893854894405991`, 0.041624697186729565`,
   0.019746996267215988`, 0.14415802609371964`, 0.10793432544039216`,
   0.362822618693736`, 0.05922100283814591`, 0.3421355433833069`, 0.07993191200692384`,
```

```
0.6226334616255764`, 0.5207521764740912`, 0.5929204690868779`, 0.08851949859515662`,
0.024743815530322788`, 0.4215260923680446`, 0.692383172946867`, 0.2844521825106663`,
0.39357078535235956`, 0.04313401864321609`, 0.04664460703269424`, 0.37557069136499965`,
0.5191583634036083`, 0.13789813647016524`, 0.26735140531088375`, 0.00789442336616597`,
0.006493044553240734`, 0.11834930490153739`, 0.4754075910671882`, 0.520063609868203`,
0.6758308528502595`, 0.11776190213599891`, 0.40489996300538067`, 0.515168231249101З`,
0.5265715848238735`, 0.42585993926787546`, 0.6595932369253765`, 0.500100101903842`,
0.10277948543833249`, 0.19363619115146724`, 0.625862692797022З`, 0.10015463157328586`,
0.4494161519649877 4`, 0.6639580030649792`, 0.2458971031642045 4`, 0.29666282432269 5`,
0.3222794353065439`, 0.632920585713636`, 0.4710608030494874`, 0.3073407065991345`,
0.5361889598429055`, 0.3154393501050501`, 0.4450246792831589`, 0.22390945967960085`,
0.22850025250821604`, 0.2732681802864583`, 0.06829244841718873`, 0.3031393426845157`,
0.05019573461237692`, 0.03554777864214709`, 0.3122836938365021`, 0.00377133915501548 5`,
0.3369145107495395`, 0.4552134222451223`, 0.11775377493499473`, 0.5175914026439326`,
0.5237186397082338`, 0.05420197407621552`, 0.5688008019027067`, 0.4996714669896485`,
0.4564187989826092`, 0.27038034952758283`, 0.01442339627684297`, 0.41882803811838465`,
0.413710764108611 5`, 0.6087810281810224`, 0.10070729583572058`, 0.27684253948583837`,
0.3756644167406118`, 0.009527840235670992`, 0.3731076200296297`, 0.3225264273249502`,
0.43326904786272125`, 0.014147089358764187`, 0.13270432978998092`,
0.0655469802774602`, 0.28043454840314586`, 0.3080004140569841`, 0.03656213543141551`,
0.65832884512829`, 0.26509051825202956`, 0.3300361486394825`, 0.4502018712692031`,
0.04773952093543998`, 0.2621757604815901`, 0.6889802476476965`, 0.629855884807345`,
0.13984631542686654`, 0.01757923964267427`, 0.6104937402581505`, 0.09934303877249051`,
0.169071230845382`, 0.45717267734061284`, 0.6504508010188645`, 0.2522272379556365`,
0.5022273971169731`, 0.3765684623791232`, 0.5149103284960848`, 0.4247873460092446`,
0.04822777488911689`, 0.22102623881592098`, 0.14559108428134138`, 0.4055362957450488`,
0.36902889555685725`, 0.43307663966311116`, 0.5696344426145243`, 0.3080624291319367`,
0.270110471565692 1`, 0.6117185734001893`, 0.0093902602305459`, 0.06278858891336392`,
0.5838417162319471`, 0.02900780551683335`, 0.3471502734734766`, 0.1893819695608724`,
0.03422406201783146`, 0.6694259146116552`, 0.14061068774787966`, 0.4968517825663361`,
0.5890820727151236`, 0.42706637558872274`, 0.10359572387306759`, 0.6597840002623023`,
0.4709404884580477`, 0.13970276121854897`, 0.19672091639783873`, 0.5599146198962612`,
0.07630275549233345`, 0.10684108574568352`, 0.5173521151833715`, 0.6103963511901886`,
0.4135177575553539`, 0.20769641660087623`, 0.3789065398505702`, 0.46778882513647213`,
0.042822853000587524`, 0.07409679433592253`, 0.32427759401300893`,
0.2540107391691374`, 0.27928681476832284`, 0.2922690826809595`, 0.46492661047249584`,
0.6814345942230915`, 0.14685698824737725`, 0.36716847668310026`, 0.23877521091896214`,
0.5399763882201027`, 0.4050280510382773`, 0.30254017129046673`, 0.14449246022735462`,
0.63453056953147`, 0.17384426833101085`, 0.4287762953332446`, 0.3755860451070956`,
0.28291452467275524`, 0.5899259142489273`, 0.24798525127403592`, 0.34024573256340473`,
0.11382921457231265`, 0.1499386193208816`, 0.4485868723048414`, 0.4555787169377248`,
0.13305545599992508`, 0.3296476973223019`, 0.16217458133313722`, 0.3649241587876635`,
0.2466542832458693 5`, 0.3251145789644134`, 0.3811604931780477`, 0.26777481529216807`,
0.3859233829913593`, 0.39843041442399274`, 0.21367901211309348`, 0.3368844187090161`,
0.5356207438138778`, 0.19991123084250617`, 0.08527272409912157`, 0.370173623904956`,
0.12751757830050825`, 0.40558520071674353`, 0.5723067926812806`, 0.6695618615760031`,
0.19040052824921672`, 0.49440562752954387`, 0.6809292090875803`, 0.4994083356666488`,
```

```
0.4032815134252552`, 0.3309663577180897`, 0.341373161045812 4`, 0.6351540088058383`,
0.605694035552284`, 0.11327180124522707`, 0.5469156660817527`, 0.027047039765383474`,
0.2638053244903391`, 0.22977235607880309`, 0.5954757029191076`, 0.5094477977916994`,
0.4387785400065811`, 0.2179903592467023`, 0.0368866574851715`, 0.5310169159067182`,
0.6707172118145324`, 0.21345597592247656`, 0.41863771171505215`, 0.3815895732995318`,
0.4248102247655767`, 0.19412481043986296`, 0.0808361503612387`, 0.5004663989257818`,
0.49574593818404367`, 0.3523246024814899`, 0.5436841577233462`, 0.1642127892550807 6`,
0.537256442191552`, 0.2221379801757591 4`, 0.29791918320031807`, 0.2598702747058729`,
0.34861726597628717`, 0.6214245108224481`, 0.4126446542208026 5`, 0.1327439460152339 8`,
0.46639540530449985`, 0.2011993651726387`, 0.29339618979577287`, 0.17565861330516108`,
0.6488071870992942`, 0.48255110726410777`, 0.3578696701881823`, 0.4835773269429428 6`,
0.5097080668089093`, 0.29783127068269544`, 0.5421039541466608`, 0.5067617238851734`,
0.5781021129517645`, 0.105674828699027`, 0.241858092141067 8`, 0.6359859147360984`,
0.21614322002293906`, 0.6514983971431387`, 0.39478548904559463`, 0.384468315540396 3`,
0.30969947260076713`, 0.32649147360537367`, 0.4947863773287675`, 0.010521962139906194`,
0.6494553238693299`, 0.4294003996078899`, 0.1929944572220853`, 0.1546951964279590 4`,
0.039917320657550825`, 0.5260988113956633`, 0.23579130471925858`, 0.3371519879451188`,
0.6290213594015168`, 0.3281784693720078`, 0.5858753959171139`, 0.020587273425080 6`,
0.32211316850911165`, 0.3464977067174497`, 0.613194480452915`, 0.2821644470030129`,
0.06766244571250635`, 0.5409083213601404`, 0.3588434777950602`, 0.08244302955417204`,
0.351451971628621 3`, 0.0714344987434734`, 0.4979492668409198`, 0.1162155963026190 2`,
0.21473308996235463`, 0.6634217560065465`, 0.6489452642437117`, 0.019836432709910534`,
0.136406482283564`, 0.37530637999389316`, 0.677472026639682 1`, 0.525560127639988 2`,
0.447229231764219`, 0.09499037882820016`, 0.542201304395878 9`, 0.3953419482650642`,
0.2166322514574046`, 0.3281954751520617`, 0.6623971077752824`, 0.1810190726271452 3`,
0.624155202345908 5`, 0.016511817410057916`, 0.539115602026595 7`, 0.6662903734546595`,
0.6561237479173125`, 0.306816532754952 6`, 0.5552057467067735`, 0.0898565845336392`,
0.41748334971300083`, 0.6295216478605794`, 0.45415946628305504`, 0.3254019880735495`,
0.6275351676978755`, 0.4008693425916874`, 0.6280778318971867`, 0.1305289119835934`,
0.3577954974155293`, 0.03815430590973501`, 0.2717405099220672`, 0.6634969529024755`,
0.3615148752261521`, 0.6842371281532844`, 0.536276579173385`, 0.6833934401359436`,
0.6905416089972782`, 0.06317887158481994`, 0.6433864303628316`, 0.2082159859106154`,
0.6738000597945417`, 0.6555720881238061`, 0.5554346153246026`, 0.6176525267784809`,
0.29729638414525184`, 0.37108743414729295`, 0.40091978301839504`, 0.08575633556693 33`,
0.39704447609159343`, 0.16543233487499953`, 0.09218986791140704`, 0.5698681076233858`,
0.6956767933281949`, 0.41950240939788275`, 0.2952400204149369`, 0.08067700407842804`,
0.25445234120507754`, 0.36144733726060085`, 0.3957455661682834`, 0.21194023467288614`,
0.5895714586824707`, 0.105130076108367`, 0.4130576892931277`, 0.1577372407632886 6`,
0.0348287500414356`, 0.13923808962973494`, 0.5763757494959347`, 0.3081722320227287`,
0.10879844961608343`, 0.063858091223713`, 0.22018399730578853`, 0.3330353253304428`,
0.345312610169551 6`, 0.1919241414515721`, 0.2412064425045216`, 0.530511984069446 1`,
0.5279098494207628`, 0.09222150904942761`, 0.624262419506506 7`, 0.5902858683907959`,
0.11540739636530517`, 0.5043633698671388`, 0.23482345851686148`, 0.222379266410820 3`,
0.3334528599286597`, 0.061433857369995404`, 0.0747635812348807`, 0.4563892275506414`,
0.41160558793918733`, 0.542439113807941`, 0.4796261046982988`, 0.4077866867246682`,
0.2591494587638222`, 0.10554280829901075`, 0.33913813127207737`, 0.127021890466895 6`,
0.191630644970842 6`, 0.67144799246926 8`, 0.37673456646458847`, 0.15856388976844382`,
```

```
0.5261913306831196`, 0.528606821109352`, 0.582104699603283`, 0.13973327283704529`,
0.5873707991867072`, 0.3189383131140051`, 0.16079910882618154`, 0.099264140210233`,
0.3617999954507791`, 0.14617602634649052`, 0.01762801396450997`, 0.6849049180681488`,
0.6698191461374112`, 0.6668137282252296`, 0.44924603983737343`, 0.006476528919752256`,
0.17729932817205374`, 0.49145878512771213`, 0.5208475936058174`, 0.334942734026195`};
```

Plot the realized probability density function based on uniform1 sample as well as the theoretical one.

In[46]:=
```
uniformDistributionPlot = Show[{Histogram[uniform1, Automatic, "ProbabilityDensity",
    Frame → {{True, False}, {True, False}}, FrameLabel → {"ϵi", "Probability Density"},
    LabelStyle → {Bold, FontFamily → "Arial", FontSize → 18, FontColor → Black},
    FrameStyle → {{Directive[Thick, Black], None}, {Directive[Thick, Black], None}},
    Epilog → {Text[Style["A", Bold, FontFamily → "Arial", FontSize → 16], {0.05, 1.65}]},
    ImageSize → 550], Plot[PDF[UniformDistribution[{0, .7}], x],
    {x, 0, .7}, PlotStyle → {Blue, Thick}]}]
```

Out[46]=

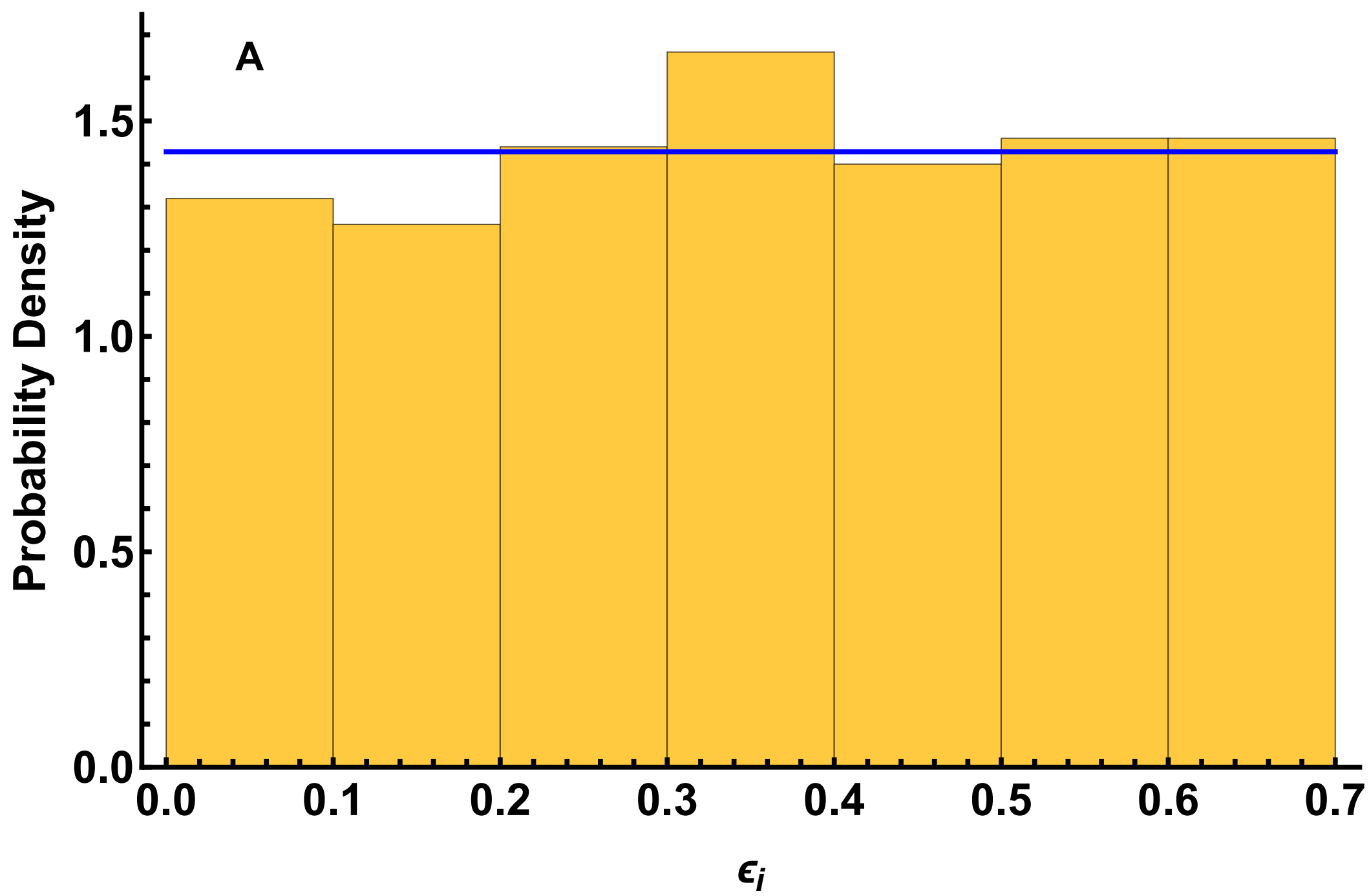

In[47]:=
```
Export["uniformDistributionPlot.svg", uniformDistributionPlot, LightDark → "Light"]
```

Out[47]=

```
uniformDistributionPlot.svg
```

Solve the 500 ASV ode equations for the uniform distribution

In[48]:=
```
soln500 = solveODEs[0.71, 24, params, uniform1];
```

500 ASVs for the uniform distribution case in absolute concentrations (μM C).

In[52]:=
```
Rasterize[xiUniformPlot =
  ListLinePlot[Evaluate[Table[{t, x[i][t]}, {i, 1, 500}, {t, 0.71, 24, 0.1}] /. soln500],
   PlotRange → All, Frame → {{True, False}, {True, False}},
   FrameLabel → {"Time (d)", "xi(t) concentration (μM)"}, LabelStyle →
    {Bold, FontFamily → "Arial", FontSize → 18, FontColor → Black}, PlotStyle → Thick,
   FrameStyle → {{Directive[Thick, Black], None}, {Directive[Thick, Black], None}},
   Epilog → {Text[Style["A", Bold, FontFamily → "Arial", FontSize → 16], {2, 3.5}]},
   ImageSize → 550], LightDark → "Light"]
```

Out[52]=

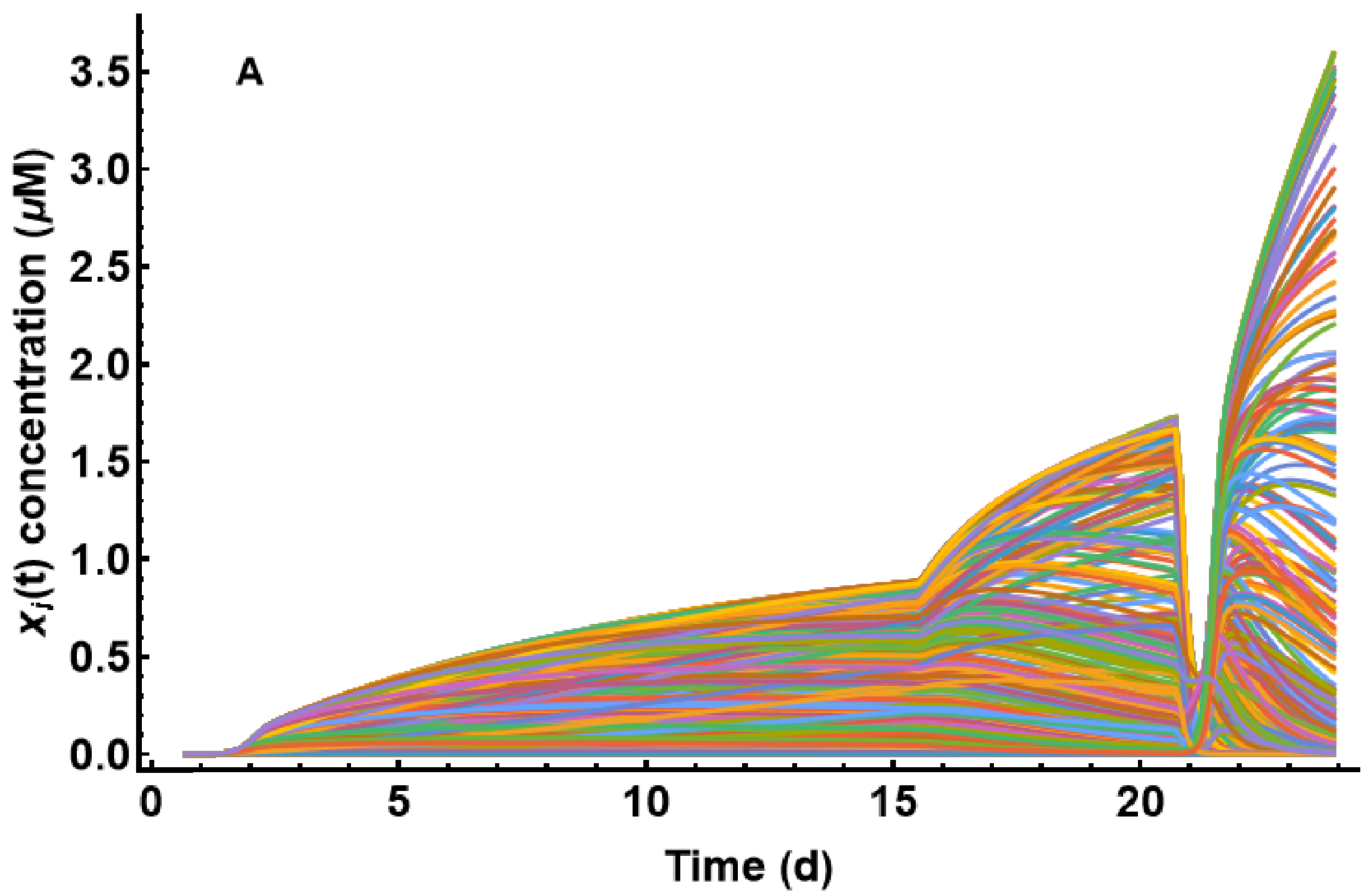

Save this plot for the manuscript

```
Export["xiUniformPlot.svg", xiUniformPlot, LightDark → "Light"]
```

Out[54]=

xiUniformPlot.svg

The Manipulate function below allows you to slide the slider below to look at profile for individual ASVs (μM C). However, this can cause issues with the front end, so it's not evaluated below

In[ ]:=
```
Manipulate[Plot[Evaluate[x[i][t] /. soln500],
  {t, 0.71, 24}, PlotLabel → "ASV: " <> ToString[i]], {i, 1, 500, 1}]
```

Plot the sum of all ASVs (μM C).

In[56]:=
```
Plot[Evaluate[Sum[x[i][t], {i, 1, 500}] /. soln500], {t, 0.71, 24}, PlotRange → All]
```

Out[56]=

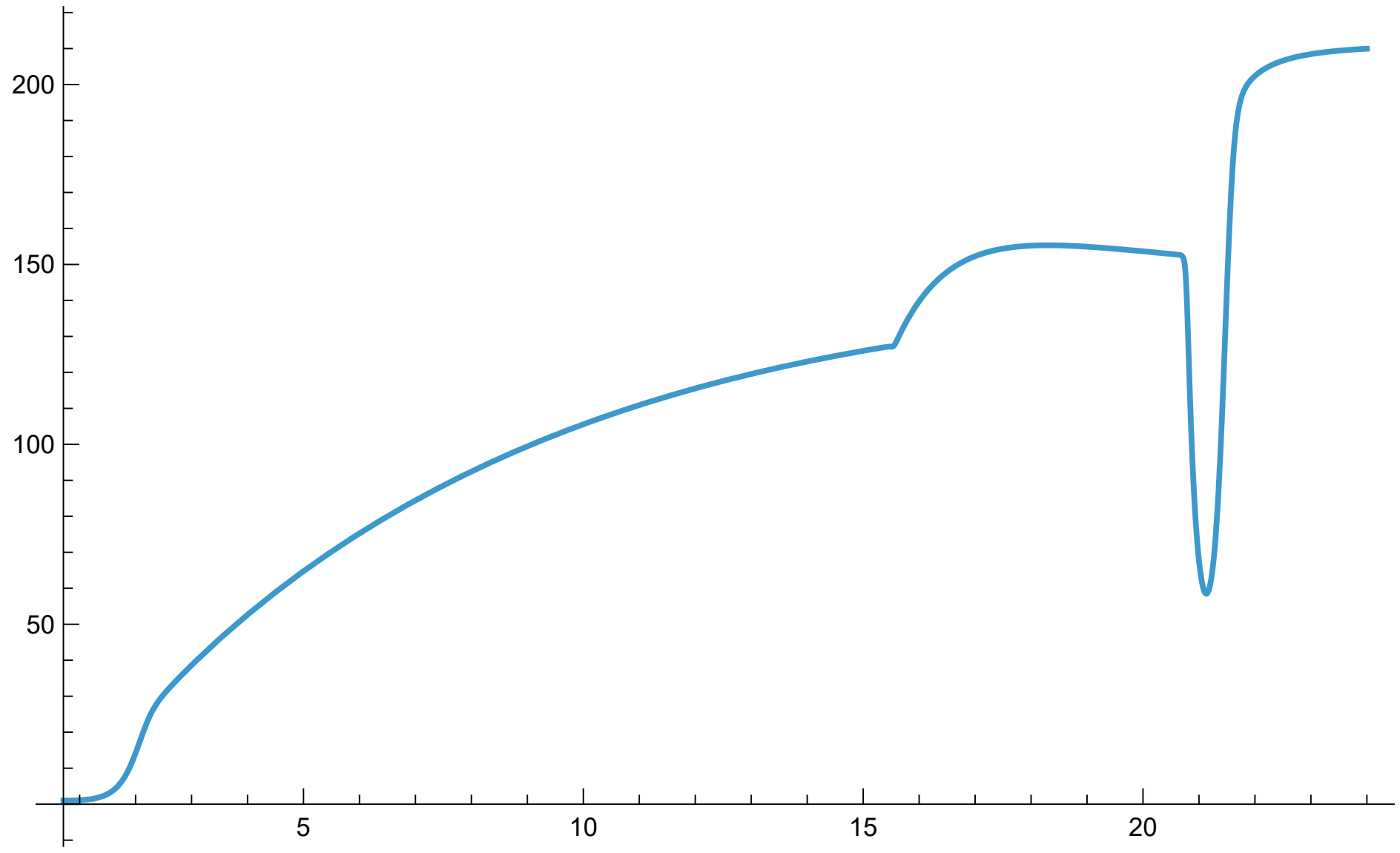

Plot the substrate concentration (μM C).

In[57]:=
```
Plot[Evaluate[s[t] /. soln500], {t, 0.71, 24}, PlotRange → All]
```

Out[57]=

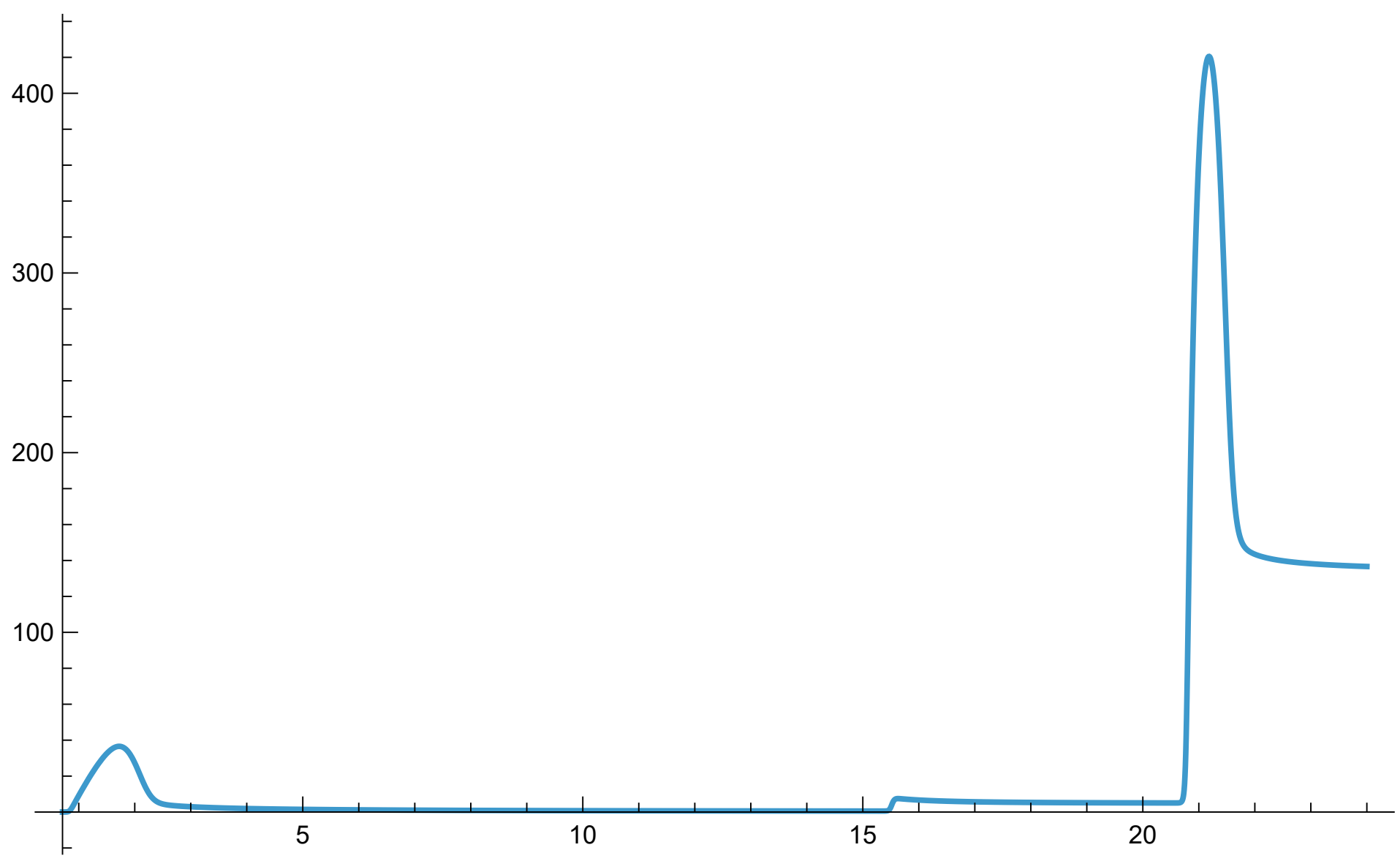

Look at diversity using relative abundance, and sample at the same times points as the MC's were. Note, because of the high diversity, the min ASV relative abundance must be set rather low, as no ASV reaches high rel abundance. Here, using 1% (that is, include any ASV that at any times exceeds 1% in abundance)

```
In[58]:= times = {0.8, 6.8, 9.5, 10.5, 15.5, 16.5, 17.6, 18.8, 19.8, 21.5, 21.8, 22.4, 22.8, 23.5};
uniformASVs = Prepend[Evaluate[Table[Table[x[i][t], {i, 1, 500}] /. soln500 /. t → times[[j]],
      {j, 1, Length[times]}]], Evaluate[Table["ASV_" <> ToString[i], {i, 500}]]]ᵀ;
uniformASVsRel = Prepend[Table[100 uniformASVs[[1 ;;, i]] / Total[uniformASVs[[1 ;;, i]]],
     {i, 2, Dimensions[uniformASVs][[2]]}], uniformASVs[[1 ;;, 1]]]ᵀ;
```

With a 1 % cutoff, there are not ASVs that make it to the end of the experiment

```
In[62]:= Rasterize[asvUniformRelGT1 = Select[uniformASVsRel, (Max[#[[2 ;;]]] > 1) &];
  ListLinePlot[Table[{times, asvUniformRelGT1[[i, 2 ;;]]}ᵀ, {i, 1, Length[asvUniformRelGT1]}],
   PlotRange → All], LightDark → "Light"]
Out[62]=
```

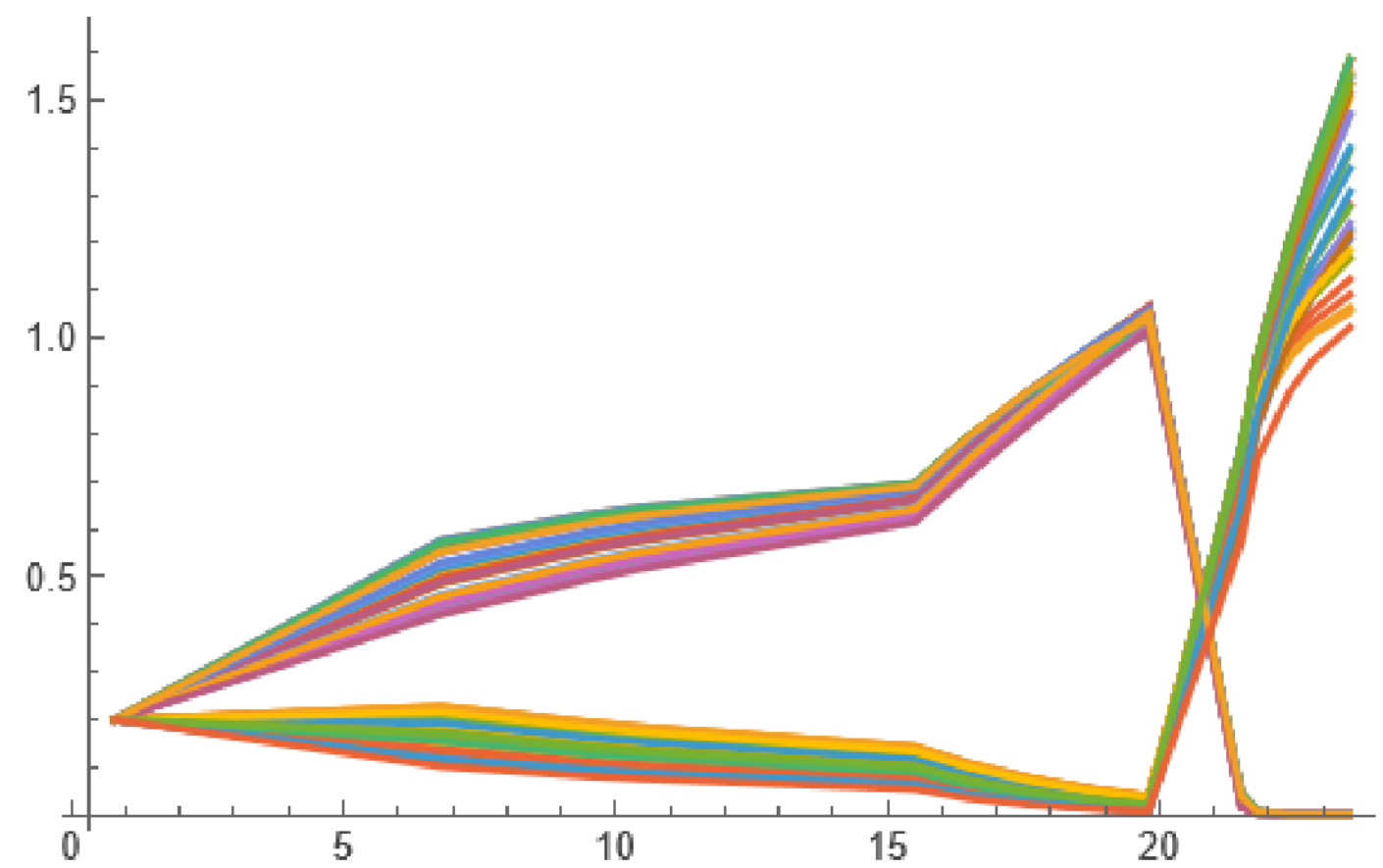

Look at including all ASVs

```
In[63]:= Rasterize[asvUniformRelGT0 = Select[uniformASVsRel, (Max[#[[2 ;;]]] > 0) &];
  ListLinePlot[Table[{times, asvUniformRelGT0[[i, 2 ;;]]}ᵀ, {i, 1, Length[asvUniformRelGT0]}],
   PlotRange → All], LightDark → "Light"]
Out[63]=
```

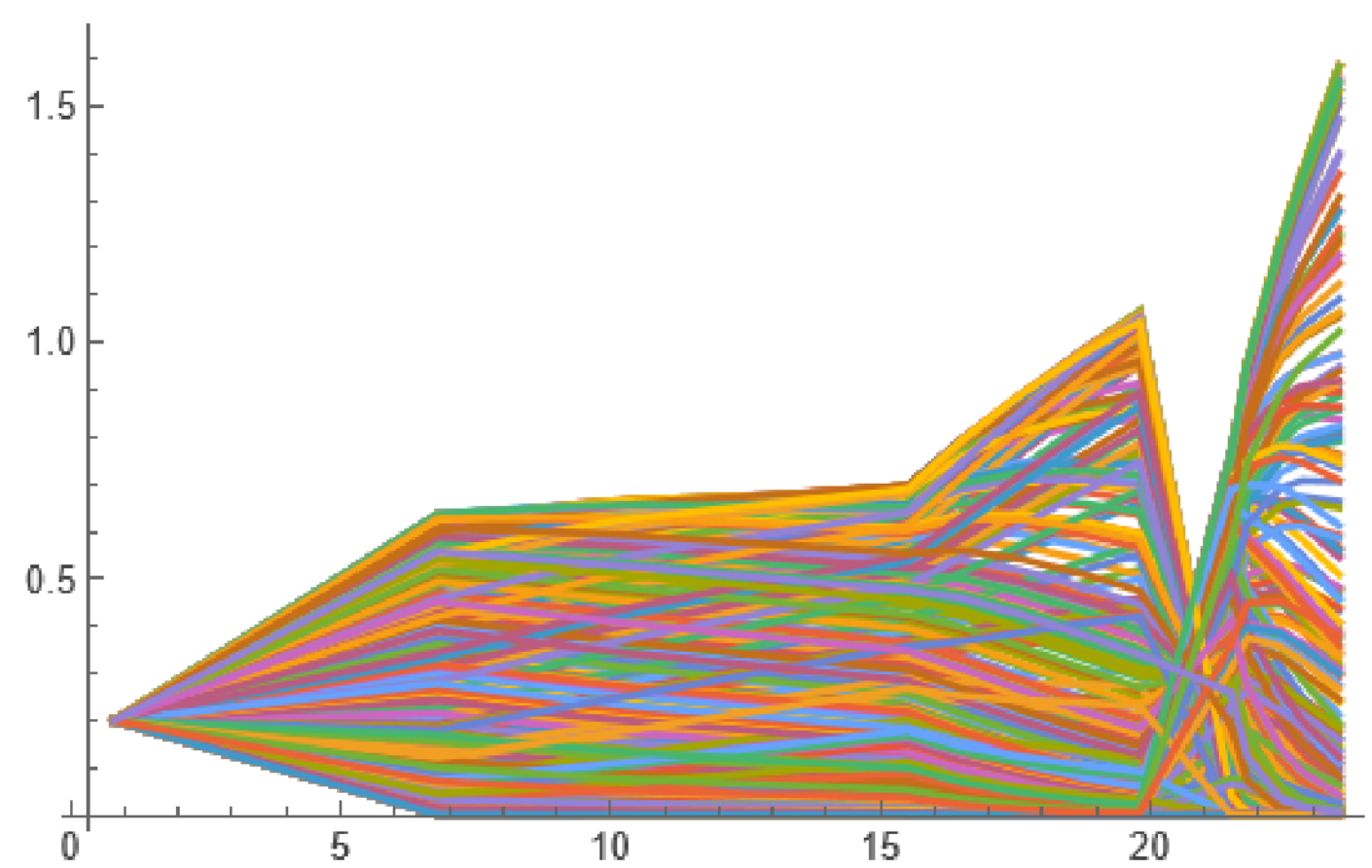

Plot absolute ASV concentrations and relative abundance at the experimental sample times as a bar plot at the 0.1% relative abundance or greater.

In[64]:=
```
asvMinAbunRel = 0.1;
asvUniformMin = Chop[N[uniformASVsRel], asvMinAbunRel];
(* this sets any ASV that is < asvMinAbunRel to 0 *)
```

In[66]:=
```
Rasterize[GraphicsRow[{Plot[Evaluate[Table[x[i][t], {i, 1, 500}] /. soln500],
    {t, 0.71, 24}, PlotRange → All, Frame → {{True, False}, {True, False}},
    FrameLabel → {"Time (d)", "xi(t) concentration (μM)"},
    LabelStyle → {Bold, FontFamily → "Arial", FontSize → 16, FontColor → Black}, FrameStyle →
     {{Directive[Thick, Black], None}, {Directive[Thick, Black], None}}, PlotStyle → Thick,
    Epilog → {Text[Style["A", Bold, FontFamily → "Arial", FontSize → 16], {2, 3.6}]}],
   BarChart[asvUniformMin[[1 ;;, 2 ;;]]ᵀ, ChartLayout → "Stacked",
    ChartLabels → {{0.8, 6.8, 9.5, 10.5, 15.5, 16.5, 17.6, 18.8, 19.8, 21.5, 21.8,
       22.4, 22.8, 23.5}, Table["", {14}]}, PlotRange → {{0.5, 14.5}, {0, 100}},
    LabelStyle → {Bold, FontFamily → "Arial", FontSize → 16, FontColor → Black},
    FrameLabel → {"Time (d)", "xi(t) (%)"}, Frame → {{True, False}, {True, False}},
    FrameStyle → {{Directive[Thick, Black], None}, {Directive[Thick, Black], None}},
    ChartStyle → 24,
    Epilog → {Text[Style["B", Bold, FontFamily → "Arial", FontSize → 16], {2, 97}]}]},
  ImageSize → 1024], LightDark → "Light"]
```

Out[66]=

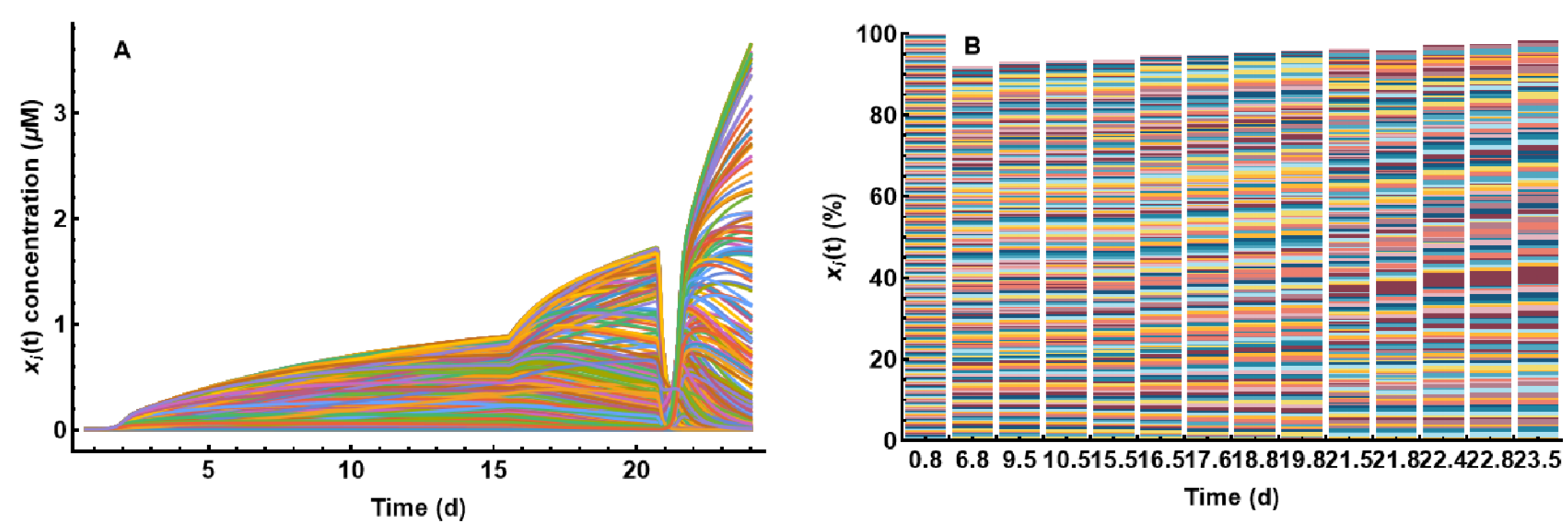

Bar Plot alone

In[67]:=
```
Rasterize[xiBarUniformPlot = xiBarChart[asvUniformMin[[1 ;;, 2 ;;]]ᵀ,
   {{0.8, 6.8, 9.5, 10.5, 15.5, 16.5, 17.6, 18.8, 19.8, 21.5, 21.8, 22.4, 22.8, 23.5},
    Table["", {14}]}, {1, 5, 6, 9, 10, 14}, "A"], LightDark → "Light"]
```

Out[67]=

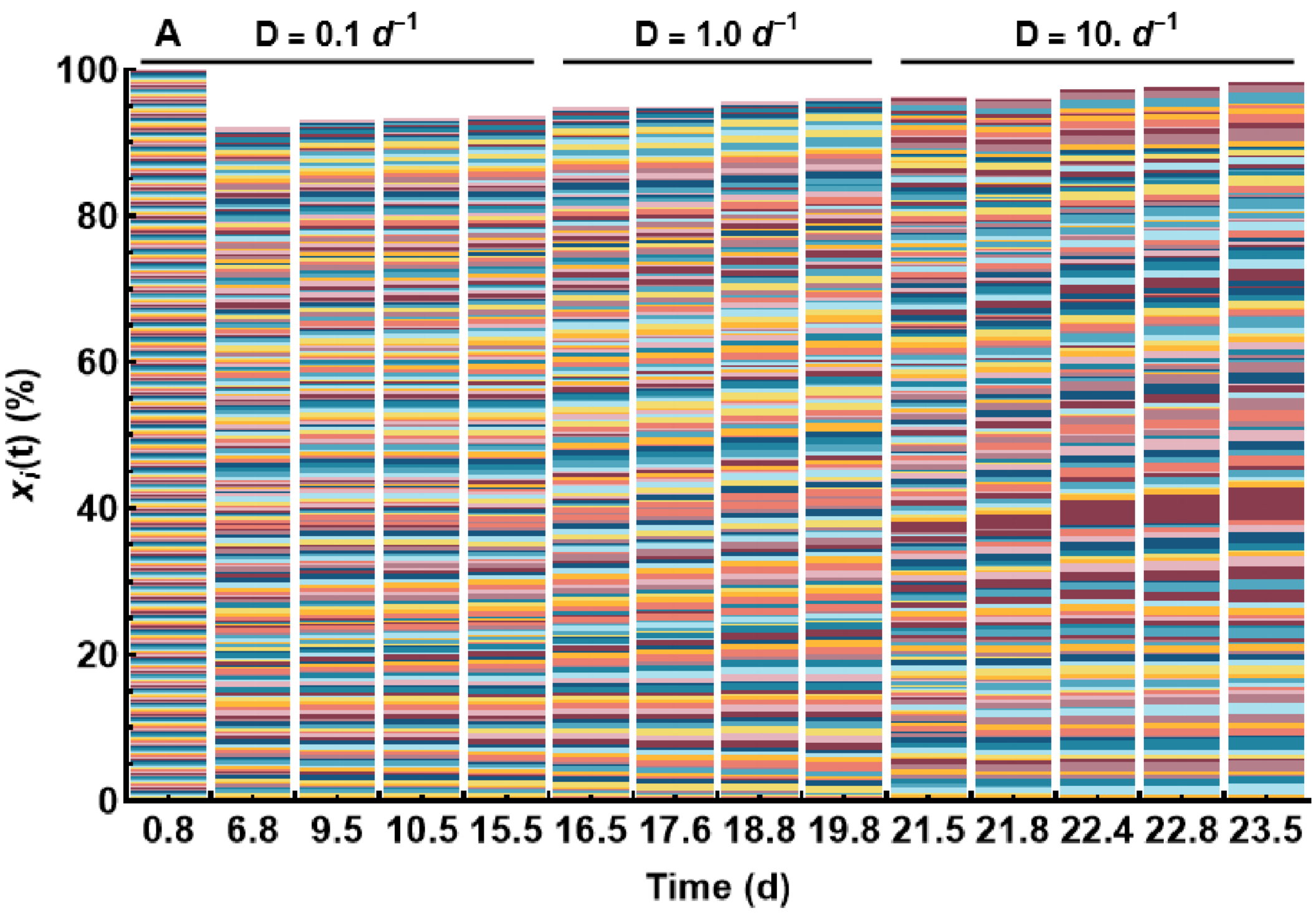

Save this plot for the manuscript

In[68]:=
```
Export["xiBarUniformPlot.svg", xiBarUniformPlot, LightDark → "Light"]
```

Out[68]=

```
xiBarUniformPlot.svg
```

Export the ASVs table for the time points that correspond to all sample times

In[155]:=

```
Export["uniformASVs.csv", Prepend[uniformASVs, Flatten[{"ASVs", times}]]]
```

Out[155]=

```
uniformASVs.csv
```

Export the relative abundances with no cutoff

In[156]:=

```
Export["uniformASVsRelative.csv", Prepend[uniformASVsRel, Flatten[{"ASVs", times}]]]
```

Out[156]=

```
uniformASVsRelative.csv
```

Uniform Distribution #2

Select another uniform distribution for statistical comparison like the experiment

In[∘]:=
```
uniform2 = RandomReal[{0, .7}, 500];
```

In[71]:=
```
uniform2 = {0.5165735824339561`, 0.1980857886199866`, 0.4143254015176081`,
   0.4783368871369138`, 0.1034833654725913`, 0.526057482775403`, 0.6003021079433981`,
```

```
0.6435859570869096`, 0.2602062799741649`, 0.6633169315693819`, 0.5081218474069846`,
0.20415279866263358`, 0.5278481183286174`, 0.11830099776804737`, 0.523325261126566`,
0.6625346195826904`, 0.6987746808677391`, 0.4129498202618569`, 0.435949718256397 6`,
0.42257969641459536`, 0.28925741679143335`, 0.1244195313625831`, 0.02570299998278025`,
0.6881473184918472`, 0.6796156477464579`, 0.02516230538110109`, 0.4818043378126342`,
0.409648335626003`, 0.4825195483064324`, 0.1961683709727149`, 0.6688246267190816`,
0.574615113628784 3`, 0.32561340661435234`, 0.04985249566718797`, 0.37626409069621713`,
0.4181657721138998`, 0.5468226976656663`, 0.18276444147990423`, 0.037845280861015795`,
0.2627361368894816`, 0.5418846992362616`, 0.47064719582168246`, 0.20647480301157084`,
0.07940096049795486`, 0.12597594941374712`, 0.1513617214295146 5`, 0.2790833221111445`,
0.43677983647314234`, 0.5348098193472786`, 0.05772422601877192`, 0.3869401255836358`,
0.1627598744695491`, 0.2753308782175764`, 0.34018732253669515`, 0.32082057521118346`,
0.21571087139181622`, 0.5091090447778415`, 0.15799593455834693`, 0.2801675363298336`,
0.04156141900660581 5`, 0.6531945737125919`, 0.5839968902315966`, 0.2227040995610915`,
0.4751439062379095`, 0.14687157027886089`, 0.5851568500847735`, 0.07917146589263901`,
0.32167245788535004`, 0.37640141733055765`, 0.6472017782757409`, 0.435300361654948`,
0.35873148823236645`, 0.6786271103209036`, 0.6248676918705169`, 0.27874126260037646`,
0.21725974667701353`, 0.009288770433434235`, 0.3675586693228632`, 0.20399747000519242`,
0.3061369447963267`, 0.3716063372293874`, 0.18824644787574618`, 0.6029677508646796`,
0.09996195575556754`, 0.0010969928648916216`, 0.3457502107579564`, 0.554652682306397 7`,
0.33334806146016005`, 0.6372037958809165`, 0.5414804746435036`, 0.4700095545044902`,
0.6389303710876073`, 0.4698993119385908`, 0.6719053769859114`, 0.4539382774410618 5`,
0.03312602461994374`, 0.06379337932149398`, 0.696419299914357`, 0.6660908156791834`,
0.42740586141557846`, 0.2682301563106432`, 0.10050975665381268`, 0.2258995810191421`,
0.2222474551426712`, 0.6776526738009501`, 0.33146883912557534`, 0.5766151782308202`,
0.5761868881689047`, 0.42066715674064303`, 0.3189877221478097`, 0.64341972416980 5`,
0.396411217481778`, 0.1614963351656744`, 0.1456500851345368`, 0.2675311506302238`,
0.11497636154691326`, 0.3884832391969504`, 0.1400015022195894`, 0.4337937485898615`,
0.11699538389462905`, 0.28985575834732413`, 0.4141110178302123`, 0.1187557383354082`,
0.31112563138752236`, 0.165415312644362 5`, 0.13313758486057248`, 0.05807637517945652`,
0.04756103904733833`, 0.11783269050096823`, 0.5444679843484153`, 0.48763480015343896`,
0.5889152395402824`, 0.43577196812212926`, 0.2307824727283171`, 0.2714166757740517`,
0.23660254203688724`, 0.1309227173141928`, 0.17909580375171175`, 0.5166233486933944`,
0.1018686812157682`, 0.6692784029229466`, 0.31085154543164073`, 0.03788924824078321`,
0.2904486623044715`, 0.5064032193802637`, 0.1610055683912066`, 0.6373753797172985`,
0.309361402248253 6`, 0.2572112778534722`, 0.5303486098396808`, 0.0297157562296492 8`,
0.6498413705112394`, 0.44849904391707796`, 0.17422446823289783`, 0.46072225340883977`,
0.5505875244476541`, 0.32468275915553546`, 0.3188275266443692`, 0.15011989962064098`,
0.006606218049896362`, 0.40070740735791954`, 0.15211744488306322`,
0.179059904786127`, 0.6967508357000094`, 0.6506125135530891`, 0.16194254638695982`,
0.1965236139836951`, 0.5416323775449332`, 0.4448315904702016`, 0.19486284788003072`,
0.6108263962751426`, 0.053293937418018356`, 0.3381688958252065`, 0.1360251395416534`,
0.48036507091131986`, 0.24931595289474118`, 0.0028739417088263775`,
0.2053810168947613`, 0.6003672062570959`, 0.05162896841627196`, 0.1559373778014812`,
0.3050100081317444`, 0.5325944961132172`, 0.314357338170673`, 0.5405917522706358`,
0.45195874514635337`, 0.1665373077096446`, 0.39566964318415154`, 0.4757429787193921`,
0.378655421208695 9`, 0.0576892018745051`, 0.0764841725570996`, 0.44758940579572104`,
```

0.2766194413865999`, 0.5124319561321193`, 0.6172561432364949`, 0.13754244439631524`,
0.43078860808025143`, 0.005732961276525339`, 0.5274973870295936`, 0.37559172935996865`,
0.3890439232286418`, 0.1741783946595058`, 0.6409909731811023`, 0.478431283406902`,
0.4066162539357674`, 0.4605509272724748`, 0.05719671367516632`, 0.2832827492943662 5`,
0.2955102427858013`, 0.10478726432102892`, 0.22198352610819427`, 0.6523463488354915`,
0.47789052703298385`, 0.671961368865394`, 0.0970575983550368`, 0.4592871708524086 7`,
0.410260276322288`, 0.12597891836974973`, 0.368966662195630 9`, 0.398902125918386 5`,
0.02101119764344183 7`, 0.02365758226395264 8`, 0.0285490112235309 3`,
0.4773651884272978`, 0.272324033230709`, 0.14263801546271349`, 0.0705598474657905 8`,
0.5932584391675224`, 0.09077035524602162`, 0.1941030196152385 7`, 0.1593021101438584 2`,
0.3187249221974382`, 0.06246434403936485`, 0.578068281559965 4`, 0.490882849031361 5`,
0.6635552946114238`, 0.36052254060296507`, 0.641789871606275 3`, 0.1816176659270245 5`,
0.2821822408997936`, 0.29837492835213975`, 0.0923021216340338 4`, 0.3915391259403646 6`,
0.5425639628824961`, 0.4630512535534921`, 0.079167175644609 4`, 0.3322473211241665 5`,
0.41740157027001223`, 0.16591127129205574`, 0.5577084518697384`, 0.550816827350362 2`,
0.26111542833480295`, 0.6805643909279124`, 0.2014791608842854`, 0.3210482793628963 7`,
0.002077119763100432 3`, 0.3441369093167423 5`, 0.4197958752962700 5`, 0.555378030921091 4`,
0.573057585045128`, 0.20713530234506705`, 0.562144115926447 1`, 0.0173454709078083 58`,
0.4906176379616878`, 0.6354480136866993`, 0.224993616265369 3`, 0.598638550012150 9`,
0.5884170625749978`, 0.194546618884576 7`, 0.154863583493949 9`, 0.2240654157899879 5`,
0.28568689142737014`, 0.3732500665888119`, 0.6086656269688304`, 0.641602247596196 2`,
0.6598468905438626`, 0.35469520754174`, 0.3283084225287973 3`, 0.544491857229311 8`,
0.38106681085792915`, 0.5011273652117225`, 0.617418606271434`, 0.633337036778360 8`,
0.34372516318416113`, 0.28378802397818936`, 0.5391350018569758`, 0.2367318328002365 7`,
0.4762879059132006`, 0.034134132184376775`, 0.3457998797755728`, 0.2467900837408557 6`,
0.6761103480527411`, 0.5704331935346867`, 0.5397040237847961`, 0.581448195372480 6`,
0.17879920378986647`, 0.1321899639651466`, 0.4367260125441208`, 0.2470698350161676 4`,
0.062110011472131244`, 0.5247834938817504`, 0.5962403057455443`, 0.1722116814767028 5`,
0.6427583374071104`, 0.011415660169478259`, 0.2339174070437512`, 0.549991609540308 3`,
0.4268317945198643`, 0.5135944209285206`, 0.4573191732009642 7`, 0.0488702814242368 8`,
0.6869240994323815`, 0.0655124711326287`, 0.4971247167683621`, 0.389315943254898 5`,
0.0318384385736491`, 0.25059989776193614`, 0.1478380001259444`, 0.1902067147359881 6`,
0.3119373699524286`, 0.12241952701633962`, 0.05261166227501523 6`, 0.467186843042741 3`,
0.515213090299927`, 0.05996982417354168 6`, 0.385215514736757 5`, 0.519090469150034 9`,
0.4458549229266444`, 0.44083405849753476`, 0.5660762036360527`, 0.1725048665270110 2`,
0.22031327564357195`, 0.3007469876293296`, 0.02043430619156649 8`, 0.2841063130439551 7`,
0.3038151108672782`, 0.17521234546137665`, 0.116090994622078 5`, 0.01224544465385446 5`,
0.1542462850361659`, 0.20453505784800496`, 0.0391586110223465 2`, 0.272363722201632 2`,
0.48127108021753595`, 0.023941985622894957`, 0.47734391822294064`, 0.500422366709651 9`,
0.4543445754825697`, 0.6012129742810082`, 0.627164087984085 6`, 0.332578762466421 8`,
0.3824425153019402`, 0.4581603966474028`, 0.639280682943009 3`, 0.0459218354341617 3`,
0.1818630492771396`, 0.3305544904355395`, 0.1928200465295152 3`, 0.046312529477067 32`,
0.414111429665016 8`, 0.08657163979388605`, 0.476101950042581 8`, 0.003902367991333144 4`,
0.43686536623730565`, 0.34946098995677044`, 0.0831934272972945 1`, 0.4475022225166316 3`,
0.17153633247409295`, 0.4742003546314202`, 0.4226984841054750 6`, 0.400750642651991 1`,
0.45172090027176304`, 0.6779312418654397`, 0.603899925855381 2`, 0.1061721293211256 3`,
0.12188009384653142`, 0.6570598186911727`, 0.1733486791108662 7`, 0.2531926762580690 7`,

```
0.6614204831777646`, 0.3571003533861503`, 0.3242339618524481`, 0.2602290303777195`,
0.3097652308184642`, 0.13112803822516894`, 0.3872956215735568`, 0.317398345044289`,
0.008366104879790282`, 0.014332623247805154`, 0.3651007593452573`, 0.07137193323925106`,
0.44160083090865987`, 0.39703322471332303`, 0.5824913231991671`, 0.012819903157630264`,
0.12182065402854936`, 0.2924247417506166`, 0.4961281007068987`, 0.4458455048184664`,
0.48363908021565627`, 0.5120347597946684`, 0.5751814444524226`, 0.561169793128174`,
0.3679347272706701`, 0.2171347873673669`, 0.32252856768694493`, 0.0579280900897543`,
0.5348232235856691`, 0.12239207111416883`, 0.17459326949606735`, 0.11043873482333566`,
0.20921837242680197`, 0.11221016184649557`, 0.14507614070745278`, 0.12566205978920697`,
0.05027700373352295`, 0.5695238262083511`, 0.6893510161689107`, 0.6435735931507789`,
0.21440275247751794`, 0.39743791734226286`, 0.6941516686846618`, 0.564821138207465`,
0.010374553534024167`, 0.038215136937455374`, 0.2365486961419384`, 0.5930592117504718`,
0.23729944952851645`, 0.2773915011736654`, 0.024829199904495614`, 0.080267340053362`,
0.4100418465746878`, 0.23222071108672082`, 0.43607658677258554`, 0.5139983656965705`,
0.3739591099053825`, 0.6952762892428317`, 0.5316543494924388`, 0.5455374720948576`,
0.42040469275229153`, 0.32503328488524086`, 0.43309543943253104`, 0.08879787074400458`,
0.4826758359272785`, 0.5994206615488782`, 0.4639811031266068`, 0.29850423574882146`,
0.17906917112887233`, 0.1953001423725409`, 0.03025929739673283`, 0.5235075500488184`,
0.568476975248497`, 0.20402773497392357`, 0.525536087702092`, 0.5456446214589117`,
0.07861011693670483`, 0.6140315978943838`, 0.4916413329115321`, 0.5714279173635017`,
0.5054078566206923`, 0.5817019603536946`, 0.5218980635560444`, 0.2985894204964593`,
0.4991199280210179`, 0.1438885971787165`, 0.6002041677762422`, 0.5524606061649737`,
0.0003203607085954241`, 0.5616684770155649`, 0.3485915402838018`, 0.4878243092682686`,
0.48232631236460133`, 0.5246158286927993`, 0.2690026031113396`, 0.6549148911997813`,
0.15589519336581348`, 0.5860115236609147`, 0.5724221689221705`, 0.00267509632032803`,
0.5709780804571862`, 0.20732464988693178`, 0.49634343601838693`, 0.6831567741198707`,
0.2359589168927726`, 0.09228088070002927`, 0.48246575223662025`, 0.30866612269726357`,
0.39427830027807875`, 0.429371617526757`, 0.03357609230469949`, 0.4123464742555827`,
0.608360013504452`, 0.2565265598855999`, 0.032315271202563944`, 0.4636726288485069`,
0.6446299162462004`, 0.2872935822591598`, 0.38686348877416554`, 0.4925557734597801`};
```

```
In[72]:= soln500b = solveODEs[0.71, 24, params, uniform2];
```

In[73]:=
```
Rasterize[xiUniformPlot =
  ListLinePlot[Evaluate[Table[{t, x[i][t]}, {i, 1, 500}, {t, 0.71, 24, 0.1}] /. soln500b],
   PlotRange → All, Frame → {{True, False}, {True, False}},
   FrameLabel → {"Time (d)", "xᵢ(t) concentration (μM)"}, LabelStyle →
    {Bold, FontFamily → "Arial", FontSize → 18, FontColor → Black}, PlotStyle → Thick,
   FrameStyle → {{Directive[Thick, Black], None}, {Directive[Thick, Black], None}},
   Epilog → {Text[Style["A", Bold, FontFamily → "Arial", FontSize → 16], {2, 3.5}]},
   ImageSize → 550], LightDark → "Light"]
```

Out[73]=

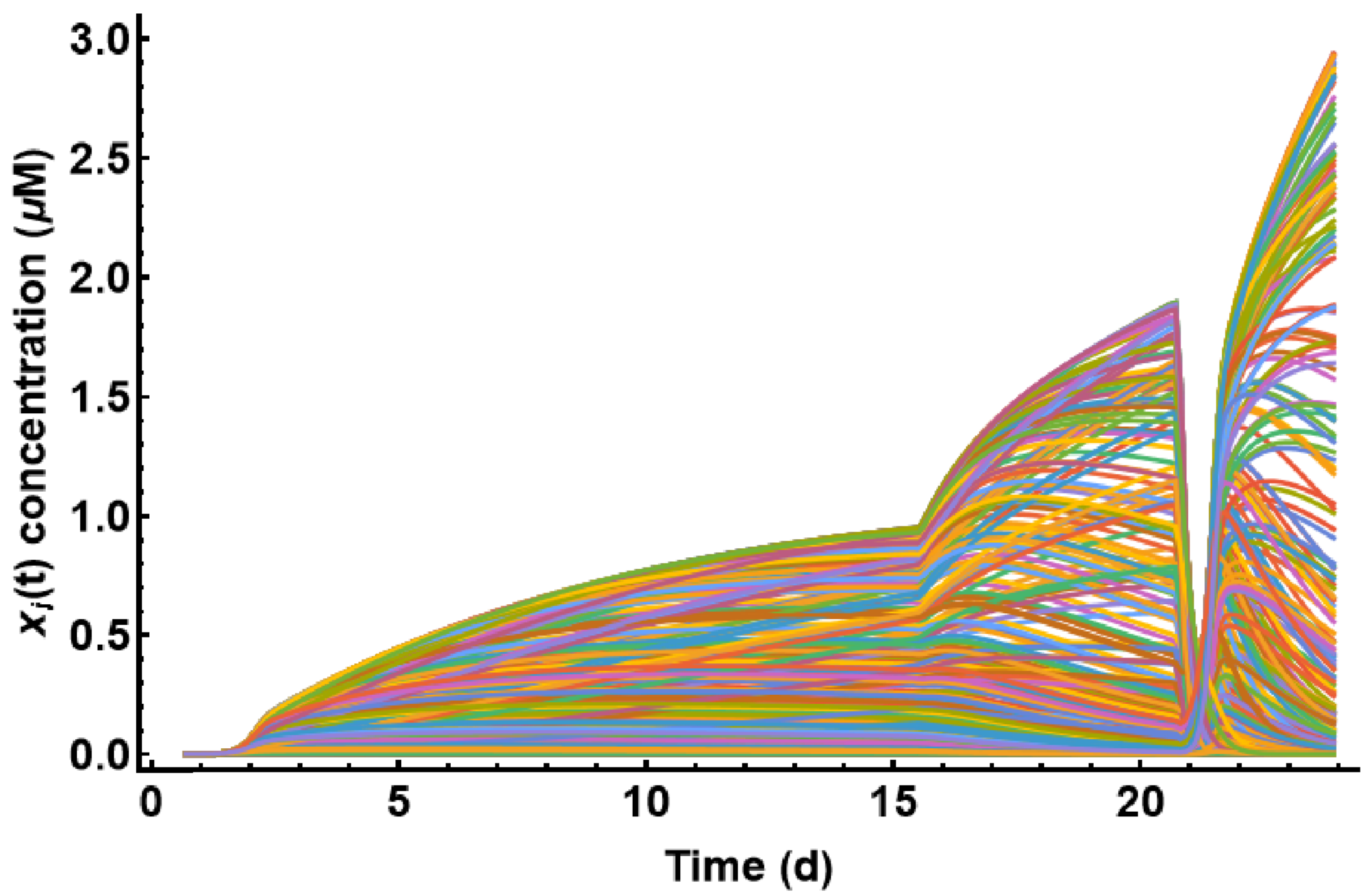

In[74]:=
```
times = {0.8, 6.8, 9.5, 10.5, 15.5, 16.5, 17.6, 18.8, 19.8, 21.5, 21.8, 22.4, 22.8, 23.5};
uniformASVsB =
  Prepend[Evaluate[Table[Table[x[i][t], {i, 1, 500}] /. soln500b /. t → times[[j]],
      {j, 1, Length[times]}]], Evaluate[Table["ASV_" <> ToString[i], {i, 500}]]]ᵀ;
uniformASVsRelB = Prepend[Table[100 uniformASVsB[[1 ;;, i]] / Total[uniformASVsB[[1 ;;, i]]],
     {i, 2, Dimensions[uniformASVsB][[2]]}], uniformASVsB[[1 ;;, 1]]]ᵀ;
```

In[77]:=
```
asvMinAbunRel = 0.1;
asvUniformMinB = Chop[N[uniformASVsRelB], asvMinAbunRel];
(* this sets any ASV that is < asvMinAbunRel to 0 *)
```

In[79]:=
```
Rasterize[xiBarUniformPlotB = xiBarChart[asvUniformMinB[[1 ;;, 2 ;;]]ᵀ,
   {{0.8, 6.8, 9.5, 10.5, 15.5, 16.5, 17.6, 18.8, 19.8, 21.5, 21.8, 22.4, 22.8, 23.5},
    Table["", {14}]}, {1, 5, 6, 9, 10, 14}, "A"], LightDark → "Light"]
```

Out[79]=

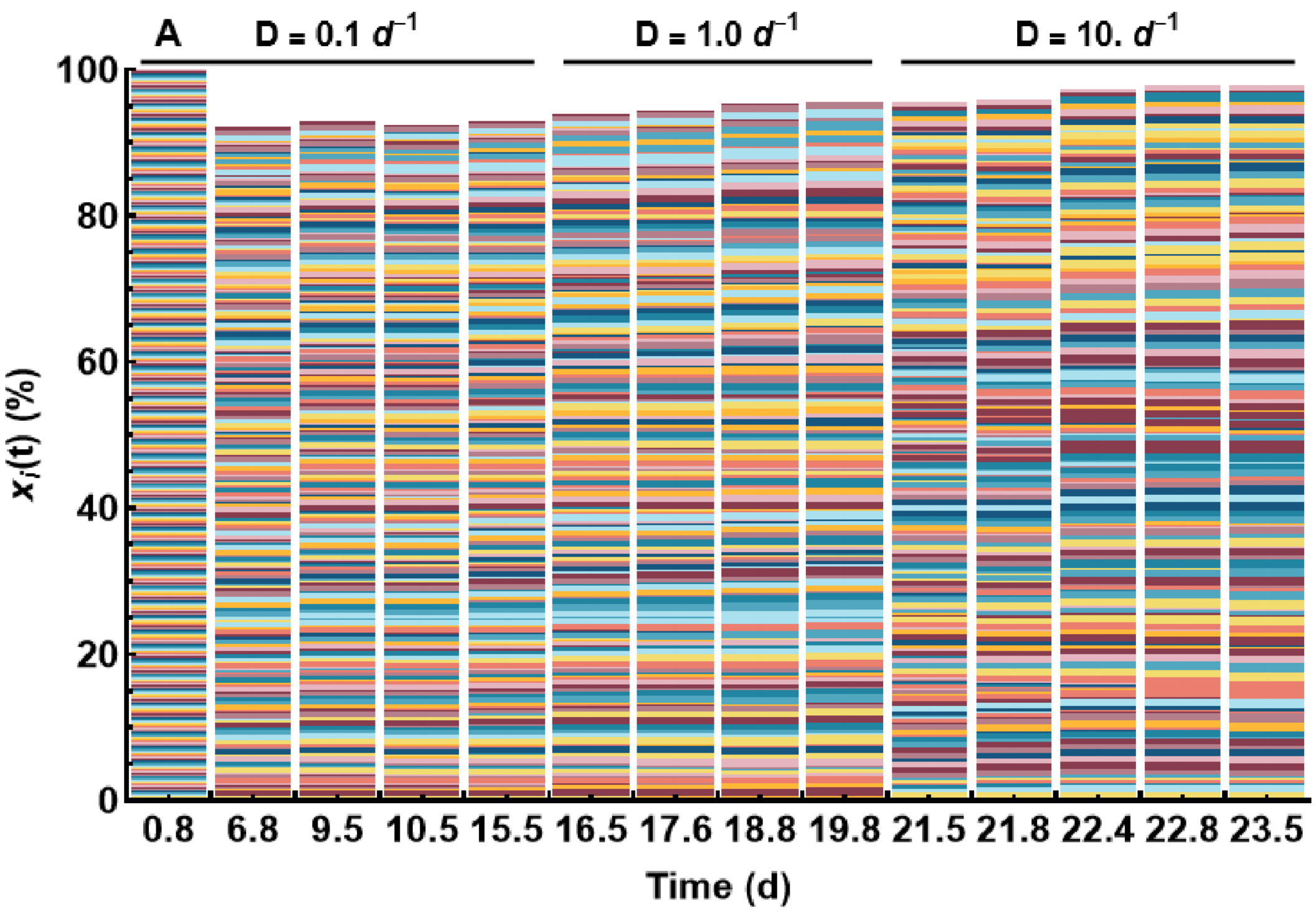

In[80]:=
```
Export["xiBarUniformPlotB.svg", xiBarUniformPlotB, LightDark → "Light"]
```

Out[80]=
```
xiBarUniformPlotB.svg
```

In[81]:=
```
Export["uniformASVsRelativeB.csv", Prepend[uniformASVsRelB, Flatten[{"ASVs", times}]]]
```

Out[81]=
```
uniformASVsRelativeB.csv
```

In[82]:=
```
Export["uniformASVsB.csv", Prepend[uniformASVsB, Flatten[{"ASVs", times}]]]
```

Out[82]=
```
uniformASVsB.csv
```

Beta distribution function Solution for MS

This is basically the same as above, except ϵ is drawn from a beta probability distribution that is skewed towards oligotrophic growth characteristics.

Run with $\alpha = 1.1$, $\beta = 13.3$

In[83]:=
```
params = {𝒟 → expDil[t, 50] (*d^-1*),
    s0 → 600, (*mmol m^-3*)
    pO2f → .2106, (*atm*)
    sal → 3, (*PSU*)
    tK → 298.15, (*K*)
    o2f → hO2[3, 298.15] .21, (*mmol m^-3*)
    kLO2 → 1.65, (*m d^-1*)
    a → π 0.114^2, (*m^2*)
    rGas → 0.082057338 × 10^-6, (*atm m^3 mmol^-1 K^-1*)
    vL → 3 / 1000, (*m^3*)
    vG → 4.74 / 1000, (*m^3*)
    fG → 15.72 / 1000, (*m^3 d^-1*)
    vM → 350, (* d^-1 *)
    kM → 5000, (* mmol m^3 *)
    m → 0.1, (*m^3 mmol^-1 d^-1*)
    α → 1.1,
    β → 13.3};
```

Look at 500 samples taken from the beta distribution for $\alpha = 1.1$ and $\beta = 13.3$

In[ ]:=
```
betaSample = RandomVariate[BetaDistribution[α, β] /. params, 500];
```

This is the beta distribution used in the manuscript

In[84]:=
```
betaSample = {0.04871417003885394`, 0.03017318186219966`, 0.012564993680192262`,
    0.13259384803132962`, 0.021620267469453208`, 0.2344049750022628`, 0.1514991154058023 5`,
    0.004684454408583026`, 0.004601369086191348`, 0.042992808450216524`,
    0.025453219010936827`, 0.09417271597016663`, 0.045696091326077035`,
    0.1328462526375 2917`, 0.0266933889076973`, 0.2629338141682852`, 0.059354581681200495`,
    0.07931635152117568`, 0.2205508867126184`, 0.02118909371323286`, 0.08205366876494401`,
    0.010931389024451262`, 0.1496050188706156`, 0.05525010558659861`, 0.1528846810909582 6`,
    0.015915678507397803`, 0.031122272663232253`, 0.0803226560606708`, 0.04314709139378374`,
    0.14315676215797007`, 0.058359206495349786`, 0.18170250048485312`, 0.03360900445255381`,
    0.02942456554931353`, 0.2087416396838662`, 0.005404580974647168 5`,
    0.0013462079542849493`, 0.07212716572067226`, 0.10450967555532742`,
    0.08744099507163389`, 0.09493157153736054`, 0.0784467555946978`, 0.0516665116899048 6`,
    0.0013341493487887 44`, 0.07578982056455534`, 0.037153957616935386`,
    0.005309644300830824`, 0.15411445948443941`, 0.009751849308989518`,
    0.07225647802771631`, 0.06936608679819813`, 0.06120727001345328`, 0.12843678844929338`,
    0.042131323662609736`, 0.01672018869895678`, 0.015215508943382137`,
    0.11225009904324758`, 0.04919497886917243`, 0.0188384047140116`, 0.037266167674637106`,
    0.11093762041353886`, 0.12789992411848625`, 0.12052621965819525`, 0.010627490500548538`,
    0.01826746581890762`, 0.023365705979300037`, 0.02368827336416486`, 0.073902030557419 3`,
    0.011295655622854098`, 0.05408094424198332`, 0.1490046520058161`, 0.12578634491357937`,
    0.004869659200823763`, 0.26630301156497094`, 0.003849534540199007`,
    0.06905215114004311`, 0.06859481953405061`, 0.028748724748098115`, 0.1484736925281987 3`,
```

```
0.2174523572636497`, 0.193708893081989`, 0.07714866769087032`, 0.022871419185523347`,
0.06341184944387644`, 0.12244641637062309`, 0.11624046267496087`, 0.07164536542764394`,
0.08039840001044087`, 0.052431646380330536`, 0.10668348177075203`, 0.09154832908079456`,
0.03257046394954066`, 0.09198072965736782`, 0.12734788662242694`, 0.1615990690664287`,
0.0007023097483443249`, 0.04599757673754196`, 0.1420387979824347`, 0.02609236076796676`,
0.05483319833189066`, 0.003454154633556137 8`, 0.05979826807602424 4`,
0.022552148777120484`, 0.023829340082174116`, 0.03753009055321549`,
0.15590155990015542`, 0.1329241242662998 5`, 0.0031347163603508955`,
0.005809975013229053`, 0.0534773192046998 3`, 0.1195926517599274`, 0.024169207539693684`,
0.033868128972670254`, 0.0047255961787532365`, 0.009001096613049143`,
0.05456996503276114`, 0.015376698457814768`, 0.03673534349017801`, 0.0442168643374372`,
0.004980503399767064`, 0.06866586347795299`, 0.03990994494206686 5`,
0.024100042679365354`, 0.06122708284578606`, 0.12067197707201006`, 0.22428778681067332`,
0.10321455171062971`, 0.061810528166035536`, 0.03429125097271478`,
0.009354432062220638`, 0.13554475676696812`, 0.05585587463340101`,
0.0011726470568291143`, 0.01148917385931319`, 0.0035962648649721933`,
0.03181949730803888`, 0.2267838072655365`, 0.1611755471264737 8`, 0.02698462446839613`,
0.010845142280690185`, 0.023816314095584358`, 0.1305232086044227 6`,
0.024311528150703785`, 0.027374071069603177`, 0.002366588015184559`,
0.024849074414056854`, 0.2439961188452165`, 0.0235668780209888`, 0.021101438545775366`,
0.003727398656108021`, 0.22215983371715115`, 0.06126381635770991`, 0.03857094915832731`,
0.010191563790865634`, 0.09609645624578363`, 0.02155436426391609`, 0.0497287884915788`,
0.027378315530424253`, 0.10126542602303192`, 0.11074720582468918`,
0.059013521119175315`, 0.08009191489674815`, 0.026025990574873595`,
0.0516453688165229`, 0.436635733572928`, 0.09505766406257224`, 0.18151396744044626`,
0.004026497589513533`, 0.08694990562535915`, 0.010689776034261059`,
0.06093180898944036`, 0.14815681455394025`, 0.017389728855135546`, 0.15376068053083183`,
0.10547790711750345`, 0.053319059254476144`, 0.007828492425429658`,
0.11547561707596206`, 0.001962946563101294`, 0.020742429167826728`,
0.09263697203414759`, 0.11065848773597486`, 0.06350342699675426`, 0.22296694310636778`,
0.08771449197198464`, 0.04811220738693807`, 0.03711701562767455`, 0.037113529737442814`,
0.1275420670922201`, 0.10890010804702391`, 0.0010086477888882534`, 0.12210310077445455`,
0.032606956528330545`, 0.03526221608701037`, 0.17583830800943565`, 0.06714135752884476`,
0.09828443656338597`, 0.0009216775278314409`, 0.1705419780457287`, 0.03811325289180692`,
0.047644995061664995`, 0.021183078103196543`, 0.13362095999997362`,
0.01524195729847747`, 0.012765560839104026`, 0.012902106693768548`,
0.09892142768556995`, 0.03809981023599537`, 0.09142479321216293`, 0.02117021052803575`,
0.01790428904971133`, 0.023014618759056738`, 0.041208699630478025`,
0.1604234626667358`, 0.13624906440479095`, 0.2172044212009787`, 0.01472299488027583`,
0.10320904273889435`, 0.03538900540536`, 0.15383534367786517`, 0.083176512728805 8`,
0.2891594570145767`, 0.012784100076480495`, 0.05813724808168957 4`,
0.07989725697937083`, 0.03750493565627171`, 0.030127139394336866`, 0.0692569612013841`,
0.1057124791160422`, 0.07636299363027825`, 0.02805523454207417`, 0.09803811080087738`,
0.004096890461484481 4`, 0.18555557437046988`, 0.13923635889454775`,
0.054409267476525254`, 0.07170089091396022`, 0.17785360980939177`, 0.08703292497589263`,
0.11066124950446958`, 0.01849525339465957`, 0.01125351097552394`, 0.02617265456166756`,
0.02870312528614626 2`, 0.0243516311096407 7`, 0.1476413191385037 6`, 0.04282363862056955`,
```

0.01638874161649552З`, 0.0024044815732956557`, 0.06621756332129385`,
0.08482442792532743`, 0.0972863022943594`, 0.06252463091101566`, 0.006323773999152156`,
0.07362815024186246`, 0.0611440977466474`, 0.07972642169305205`, 0.1447556892689672`,
0.09928994351387667`, 0.020794784747151036`, 0.050520000331074226`,
0.12141743932292878`, 0.036350985169579256`, 0.01753499671893606`, 0.0998144140966093`,
0.3040912124150068`, 0.21577960371639454`, 0.1228229840148005 7`, 0.00560971331127957З`,
0.014979100908650154`, 0.0317019843479898 8`, 0.008771309668806833`,
0.12799598858285297`, 0.18253831146701313`, 0.22604220035805841`, 0.022101791111936452`,
0.17276189858838992`, 0.08861263428291366`, 0.0481247271989185 6`, 0.046674493469162186`,
0.021464146507729518`, 0.16044991188722343`, 0.0466983600698937`, 0.0695767764708827З`,
0.09043801855017315`, 0.1644442606878382`, 0.12545534607706213`, 0.0826074242900713З`,
0.08309558671739452`, 0.014564491167656008`, 0.07667963494845564`,
0.051622435206747475`, 0.12374328132302406`, 0.002498968309387258`,
0.013030313862055545`, 0.006428129906045408`, 0.0500162607716743З`,
0.026443982143654638`, 0.0539879735120826`, 0.1312999823623269`, 0.11512505634597918`,
0.21686950178922165`, 0.13585031425223854`, 0.0721000435565657 7`, 0.0479386880519318З`,
0.09283559178732992`, 0.08969893726785652`, 0.1127165179660344`, 0.10775185052769168`,
0.082934653731241`, 0.06823372191103245`, 0.06587945352658324`, 0.2328410568252529`,
0.29667855682759264`, 0.2417366751952173`, 0.02268594766727997`, 0.1053665697509538 9`,
0.041595829142700776`, 0.022853557801980416`, 0.1421097807446515 8`, 0.0832995335530726`,
0.042037241378529315`, 0.1356399267952631`, 0.1019334755670714`, 0.017516502335782242`,
0.09479937599913722`, 0.10588947742985351`, 0.027886623944396186`, 0.0482951140874707З`,
0.0537767186800631 2`, 0.027548383900668754`, 0.10032500675730449`,
0.0025943474034798727`, 0.0002914995586228621`, 0.11763744700827708`,
0.10308827759380966`, 0.07394200450453615`, 0.10622159011543529`, 0.014848515802661616`,
0.25550171739791566`, 0.205313181183687З`, 0.0017261960196748958`, 0.06294153015556311`,
0.01920130174043932`, 0.03554558881839803`, 0.0791365044139423`, 0.026971779170970305`,
0.07574165069700076`, 0.1421689759972292`, 0.09329687854788936`, 0.05024255981023416`,
0.0490125971519095`, 0.022976297480557004`, 0.029544565269354898`, 0.15065479884821742`,
0.008209166237822623`, 0.17455831112154385`, 0.013752289171807503`,
0.07422293537549755`, 0.1639564288054622`, 0.14087312121450782`, 0.014014135664685779`,
0.0027830560397054402`, 0.16666839002916034`, 0.026505413568927255`,
0.11446821433343855`, 0.1105076221966884 4`, 0.00541900015390286`, 0.037520865133616896`,
0.030772458132362467`, 0.05280786810800987`, 0.03407556710718542`, 0.03084800257306437`,
0.10102930634113923`, 0.13044375778720677`, 0.030600156418795557`,
0.010512867983209667`, 0.1274287373380895`, 0.17745371321519915`, 0.11057254754925154`,
0.007516639911862173`, 0.026979097585571113`, 0.002611592186387166`,
0.0337798156974438`, 0.05597223108797968`, 0.04911962522972667`, 0.13053360462565602`,
0.12067387539058357`, 0.11878864049784978`, 0.1250216585092904`, 0.1207410013046579 4`,
0.20558704294754135`, 0.14246463035757395`, 0.07402279649358105`, 0.07786475965990819`,
0.0340581506060693`, 0.08041523444578938`, 0.01558213710139311`, 0.16397534159102004`,
0.11779291952672884`, 0.017105792256956742`, 0.08357290011776101`, 0.23607846408267072`,
0.040883668439963376`, 0.03321036888035268`, 0.04703692179272006`, 0.1166662308048623`,
0.0038126213775626595`, 0.07725972617027421`, 0.01032278551667974`,
0.13436662312586525`, 0.36570938724633445`, 0.10770468048277729`, 0.01296953141933141`,
0.0473113270066783 6`, 0.044446624328923344`, 0.00618235122746949`, 0.05300837236083615`,
0.10050303763689357`, 0.190210728684964`, 0.0513335253832927`, 0.11537750376189984`,

```
0.4878235997476536`, 0.009631465023873832`, 0.16066235202414658`, 0.17448920272235646`,
0.0816645835667271`, 0.21018687411435585`, 0.06529908683101382`, 0.14954184309941784`,
0.03624690107933765`, 0.02128298021358151`, 0.025108762686227807`, 0.10885757617356026`,
0.015409928243411416`, 0.05263634391372248`, 0.013831751270294623`, 0.1400540494420741`,
0.00406843759633261`, 0.07963999651514335`, 0.07330640592822639`, 0.07304679090028407`,
0.005040331979276583`, 0.05734420056467882`, 0.021502024380563593`,
0.03827259182580616`, 0.10814138756654423`, 0.12808460159445015`, 0.06217340387635631`,
0.031154479356549615`, 0.12116323038579138`, 0.010500654548535904`,
0.06516840129529362`, 0.03301747044044952`, 0.07547029436558676`, 0.04258003424194382`,
0.04384589188197849`, 0.03184852282369526`, 0.0780225475565889`, 0.01872239907846052`,
0.07086199931284655`, 0.010902596630949998`, 0.13966318078624584`,
0.040832296776574635`, 0.037824259976154990`, 0.06320106847160721`,
0.052262142440238066`, 0.046381696437415730`, 0.04276469913748643`, 0.12672730542857397`,
0.0456168335292967`, 0.05412109296934094`, 0.11581652833622333`, 0.078930530472329`,
0.04601024282871352`, 0.1506702363443959`, 0.07005305360127806`, 0.047111011427714684`,
0.017198257945144423`, 0.111219962811526`, 0.205405329278806`, 0.09596661055086143`,
0.0031196112401487386`, 0.049203736550980134`, 0.188870226685046`, 0.09812922801643555`,
0.04157488368849383`, 0.1878757151311634`, 0.06781604975098905`, 0.13737459056442122`,
0.0017052641579928189`, 0.01147809467931195`, 0.10381208418397085`,
0.09294383557583803`, 0.025770371736442615`, 0.0766033200309779`, 0.04359861555511431`,
0.0015258878654216925`, 0.130528588016478`, 0.15164736597095563`};
```

In[85]:=
```
betaDistributionPlot = Show[Histogram[betaSample,
   Automatic, "ProbabilityDensity", Frame → {{True, False}, {True, False}},
   FrameLabel → {{"Probability Density", ""}, {"ϵi", ""}},
   LabelStyle → {Bold, FontFamily → "Arial", FontSize → 18},
   FrameStyle → {{Directive[Thick, Black], None}, {Directive[Thick, Black], None}},
   Epilog → {Text[Style["B", Bold, FontFamily → "Arial", FontSize → 16], {0.03, 10}]},
   ImageSize → 550], Plot[PDF[BetaDistribution[α, β] /. params, x],
   {x, 0, 1}, PlotStyle → {Thick, Blue}, PlotRange → {{0, 0.7}, All}]]
```

Out[85]=

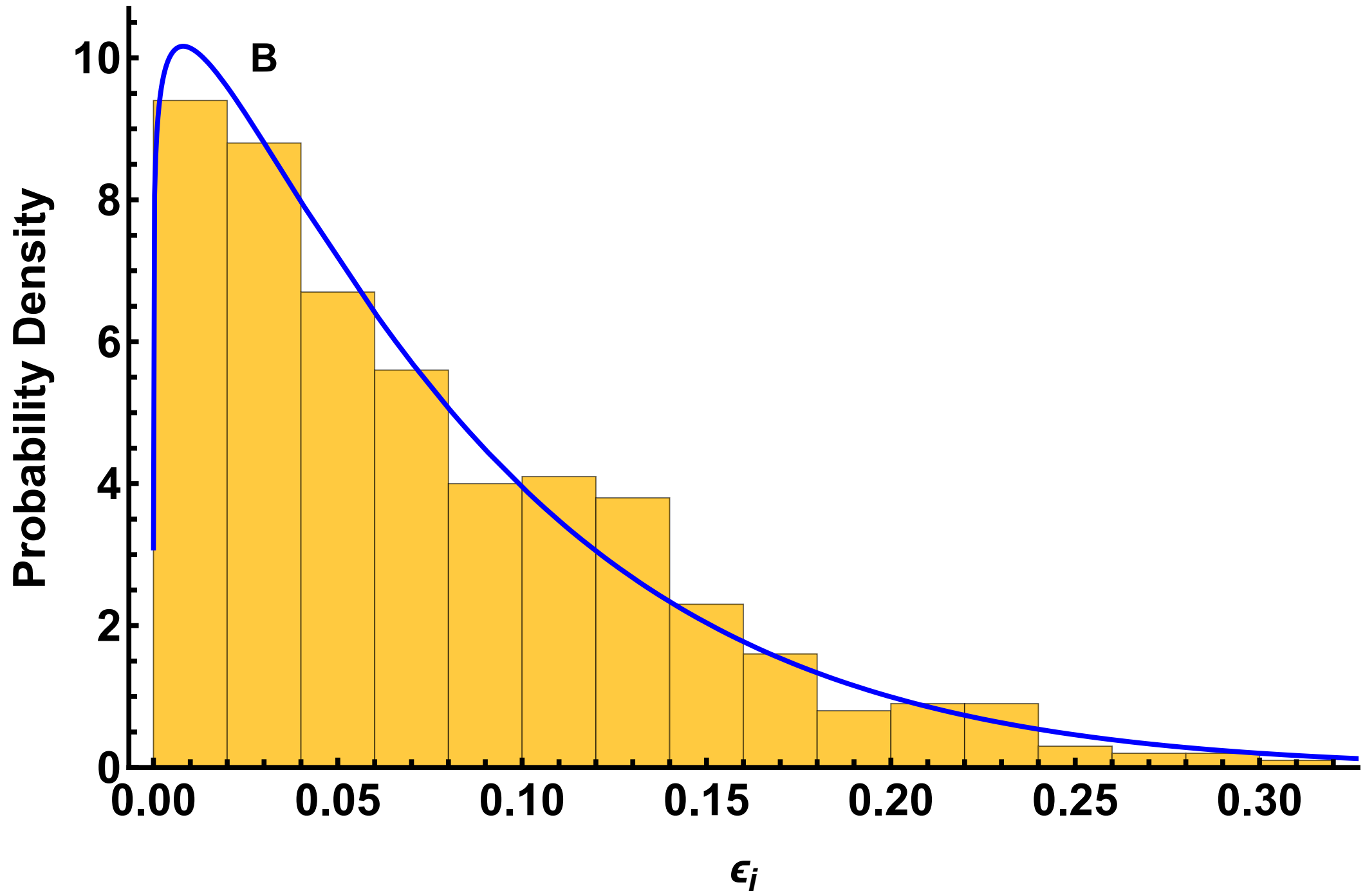

In[86]:=
```
Export["betaDistributionPlot.svg", betaDistributionPlot, LightDark → "Light"]
```

Out[86]=
```
betaDistributionPlot.svg
```

Solve ODEs for 500 ASVs again, but using the sampled beta distribution.

In[87]:=
```
soln500beta = solveODEs[0.71, 24, params, betaSample];
```

Plot the absolute concentrations over time

In[88]:=
```
Rasterize[xiBetaPlot = ListLinePlot[
    Evaluate[Table[{t, x[i][t]}, {i, 1, 500}, {t, 0.71, 24, 0.1}] /. soln500beta],
    PlotRange → All, Frame → {{True, False}, {True, False}},
    FrameLabel → {"Time (d)", "xi(t) concentration (μM)"}, LabelStyle →
     {Bold, FontFamily → "Arial", FontSize → 18, FontColor → Black}, PlotStyle → Thick,
    FrameStyle → {{Directive[Thick, Black], None}, {Directive[Thick, Black], None}},
    Epilog → {Text[Style["B", Bold, FontFamily → "Arial", FontSize → 16], {2, 70}]},
    ImageSize → 550], LightDark → "Light"]
```

Out[88]=

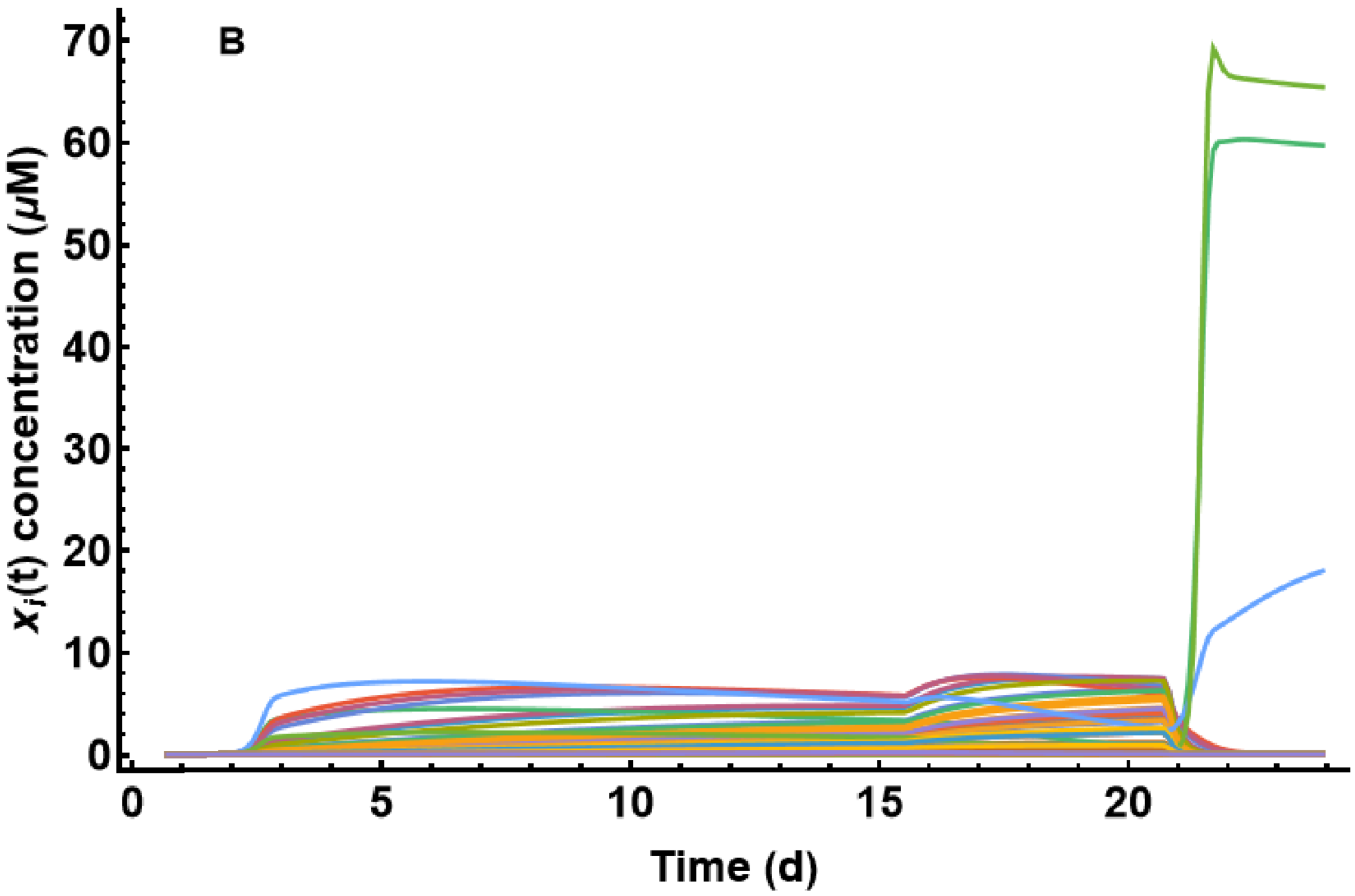

In[89]:=
```
Export["xiBetaPlot.svg", xiBetaPlot, LightDark → "Light"]
```

Out[89]=

```
xiBetaPlot.svg
```

Look at relative abundances

In[90]:=
```
times = {0.8, 6.8, 9.5, 10.5, 15.5, 16.5, 17.6, 18.8, 19.8, 21.5, 21.8, 22.4, 22.8, 23.5};
betaASVs = Prepend[Evaluate[Table[Table[x[i][t], {i, 1, 500}] /. soln500beta /. t → times[[j]],
      {j, 1, Length[times]}]], Evaluate[Table["ASV_" <> ToString[i], {i, 500}]]]ᵀ;
betaASVsRel = Prepend[Table[100 betaASVs[[1 ;;, i]] / Total[betaASVs[[1 ;;, i]]],
     {i, 2, Dimensions[betaASVs][[2]]}], betaASVs[[1 ;;, 1]]]ᵀ;
```

Plot ASVs that have a relative abundance > 5 %

In[93]:=
```
Rasterize[asvBetaRelGT5 = Select[betaASVsRel, (Max[#[[2 ;;]]] > 5) &];
  ListLinePlot[Table[{times, asvBetaRelGT5[[i, 2 ;;]]}ᵀ, {i, 1, Length[asvBetaRelGT5]}],
   PlotRange → All], LightDark → "Light"]
```

Out[93]=

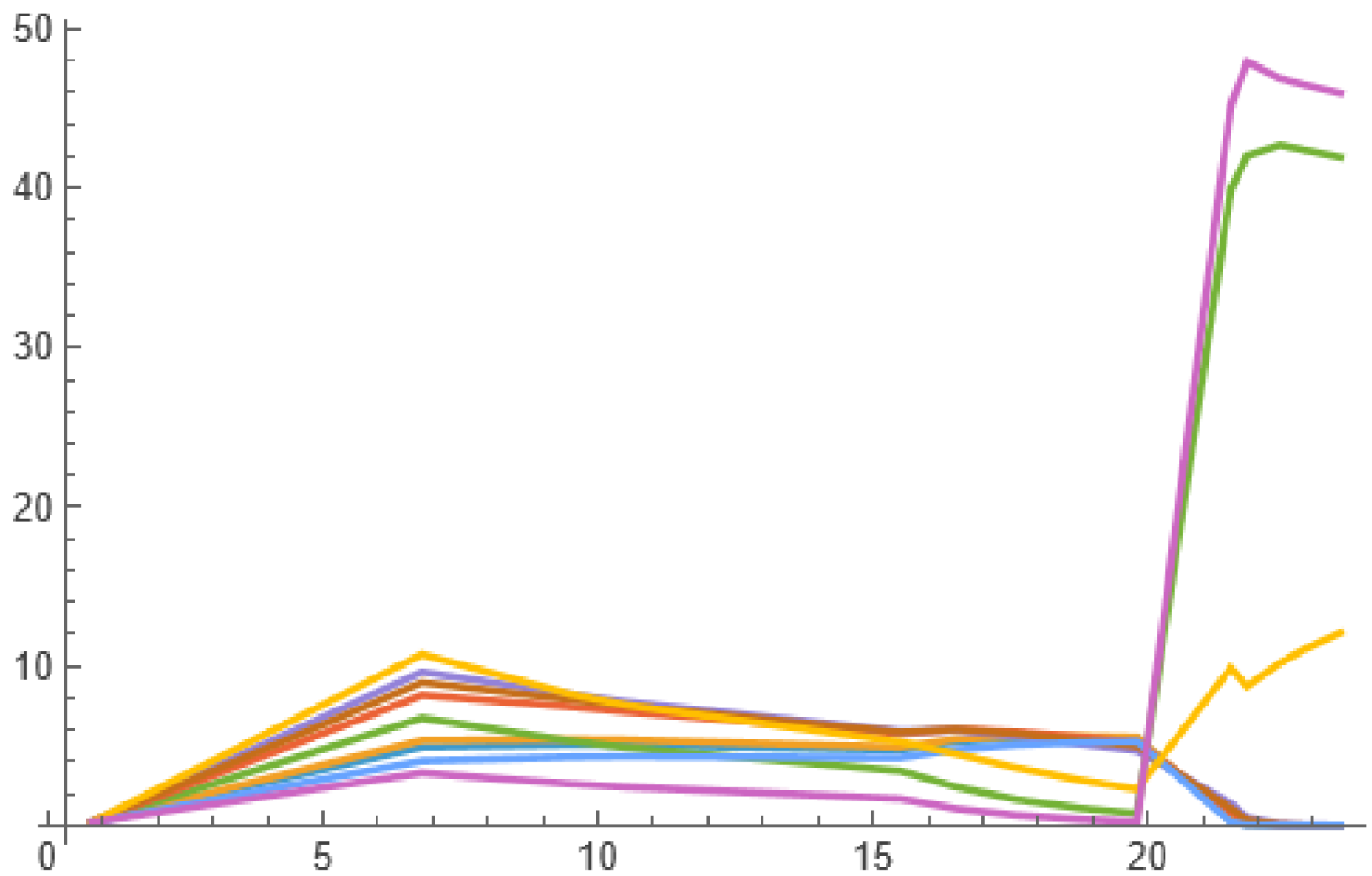

Look at bar plot with relative abundances > 0.1%.

In[94]:=
```
asvMinAbunRel = 0.1;
asvBetaMin = Chop[N[betaASVsRel], asvMinAbunRel];
(* this sets any ASV that is < asvMinAbunRel to 0 *)
```

```
In[96]:= Rasterize[xiBarBetaPlot = xiBarChart[asvBetaMin⟦1 ;;, 2 ;;⟧ᵀ,
    {{0.8, 6.8, 9.5, 10.5, 15.5, 16.5, 17.6, 18.8, 19.8, 21.5, 21.8, 22.4, 22.8, 23.5},
     Table["", {14}]}, {1, 5, 6, 9, 10, 14}, "B"], LightDark → "Light"]
```

Out[96]=

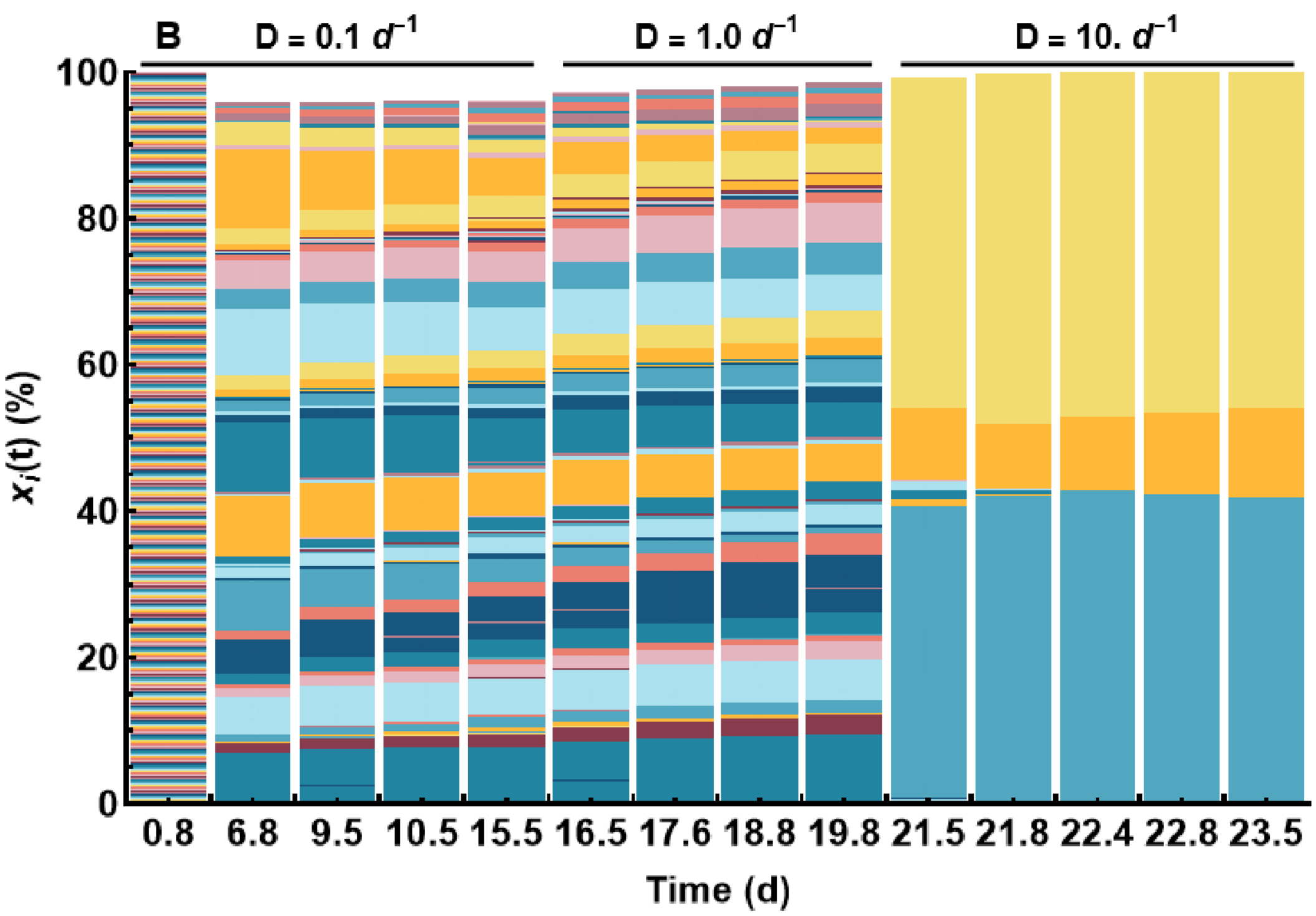


```
In[97]:= Export["xiBarBetaPlot.svg", xiBarBetaPlot, LightDark → "Light"]
```

Out[97]=

xiBarBetaPlot.svg

For a single ASVs (this evaluation has been removed from the notebook)

```
In[ ]:= Manipulate[Plot[Evaluate[x[i][t] /. soln500beta],
   {t, 0.71, 24}, PlotLabel → "ASV: " <> ToString[i]], {i, 1, 500, 1}]
```

Sum of all ASVs (absolute concentration in μM C)

In[98]:=
```
Plot[Evaluate[Sum[x[i][t], {i, 1, 500}] /. soln500beta], {t, 0.71, 24}, PlotRange → All]
```

Out[98]=

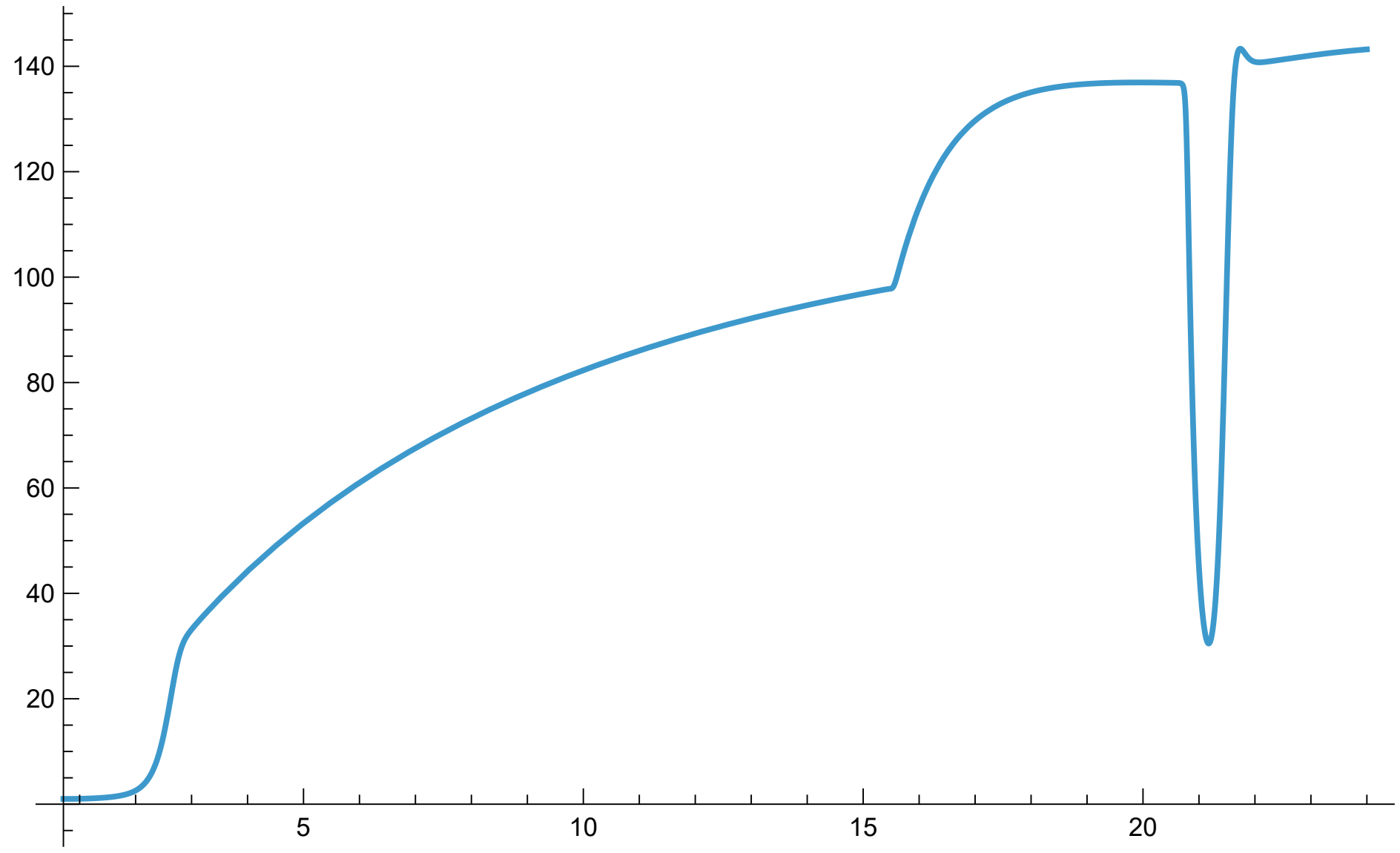

Substrate concentration (μM C)

In[99]:=
```
Plot[Evaluate[s[t] /. soln500beta], {t, 0.71, 24}, PlotRange → All]
```

Out[99]=

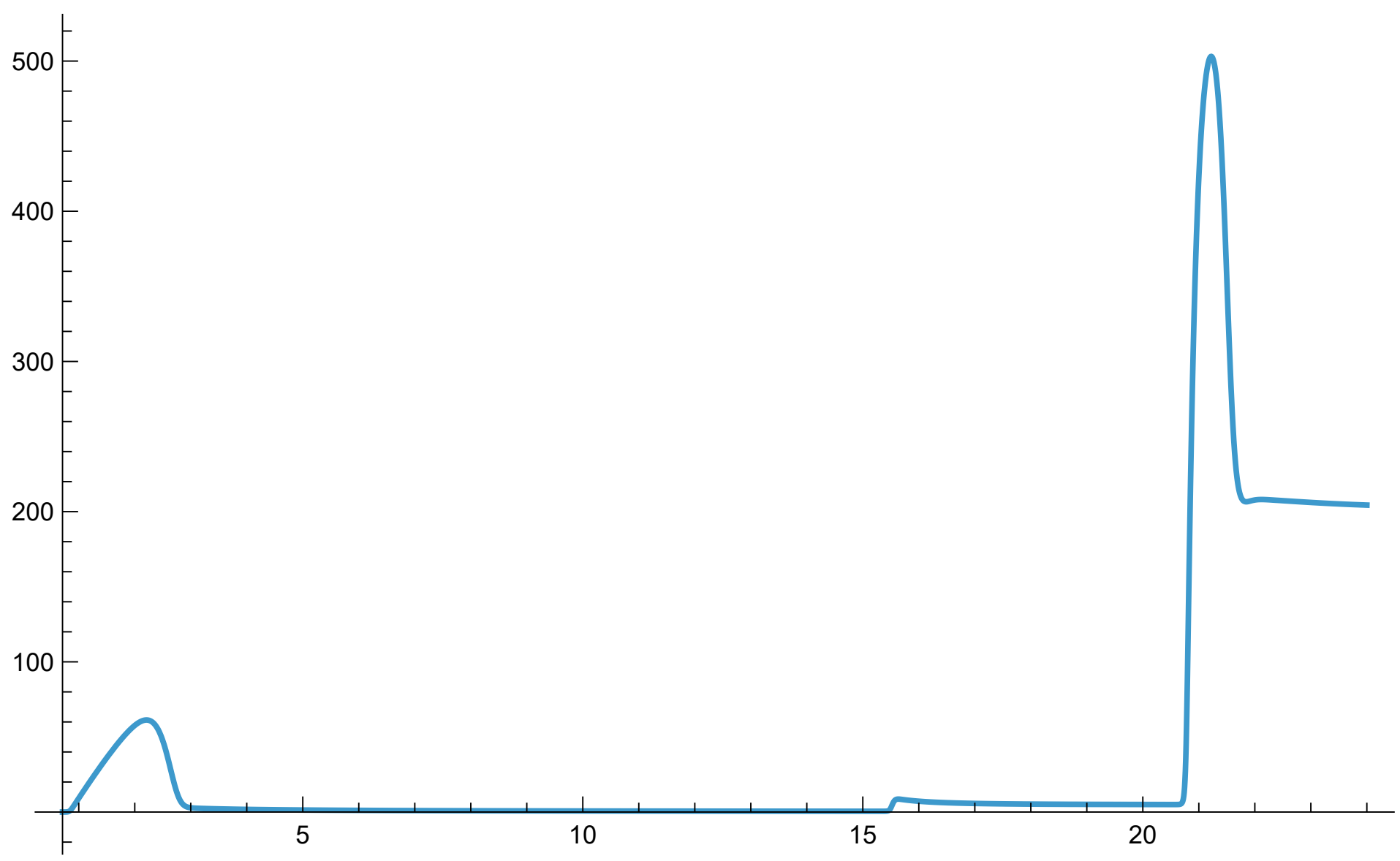

Export the ASVs for the beta distribution simulation

In[153]:=
```
Export["betaASVs.csv", Prepend[betaASVs, Flatten[{"ASVs", times}]]]
```

Out[153]=
```
betaASVs.csv
```

In[154]:=
```
Export["betaASVsRelative.csv", Prepend[betaASVsRel, Flatten[{"ASVs", times}]]]
```
Out[154]=
```
betaASVsRelative.csv
```

Beta Distribution #2

In[ ]:=
```
betaSampleB = RandomVariate[BetaDistribution[α, β] /. params, 500];
```
In[103]:=
```
betaSampleB = {0.05839435028147007`, 0.017130115740773093`, 0.3818713220125133`,
   0.08127155963949621`, 0.022914871547148632`, 0.01487719454832803`,
   0.0027823040606488533`, 0.1652520514192369`, 0.02703296352699693`,
   0.1784663582608669`, 0.03441818017748306`, 0.03960929636809701`, 0.11523276989993306`,
   0.06399453792119747`, 0.1394687983579816`, 0.05939453886861214`, 0.05868506609193801`,
   0.008889687199764284`, 0.10085252710162547`, 0.019760428575211802`, 0.114057669267189 7`,
   0.26204842925265276`, 0.10345665631734609`, 0.008110936887527122`, 0.149630936698004 7`,
   0.08468676177731703`, 0.022522473984154214`, 0.01228043558627182 9`,
   0.022494738023104217`, 0.005523866726018677`, 0.03568552777184305`,
   0.29192504395606067`, 0.12800629820143952`, 0.02022872471766756`, 0.13741641421093376`,
   0.023073848542150506`, 0.14748325837978668`, 0.03535883689797409`,
   0.004115608640459704`, 0.009346203976004384`, 0.08656199431859946`,
   0.0639692548828862`, 0.07492354123613465`, 0.12040638679347285`, 0.02541573856032714`,
   0.01754861866924918`, 0.040062069003748105`, 0.06054741026234647`, 0.08522253875759474`,
   0.029762437495614438`, 0.2100449873325659`, 0.0011354581545743837`,
   0.1344572600319794 7`, 0.025790445633291866`, 0.11879127418861948`, 0.11261749914195784`,
   0.0886975395219119 8`, 0.01686515682033391`, 0.1820949690068539`, 0.017578965153871084`,
   0.10520607202459703`, 0.00022400130916982615`, 0.019267154423809447`,
   0.02493604493167722`, 0.13401963381924187`, 0.2472950377083757`, 0.09076592394220666`,
   0.11781836738519624`, 0.006204720654685361`, 0.05102814170802059`,
   0.051945412698574736`, 0.006618664550995789`, 0.17110285772482237`,
   0.02969553341716303`, 0.021699759981477343`, 0.02847440123577255 4`,
   0.09971751680236897`, 0.05907321046292947`, 0.020207144934675545`, 0.021605482431777 17`,
   0.028679674538883548`, 0.04415878130998605`, 0.021249109700996617`,
   0.03844818522143149`, 0.021182682798526992`, 0.003095178759429924 4`,
   0.028152700265128724`, 0.21830726392670782`, 0.06786658752847553`,
   0.021825464672670415`, 0.02633999380331808`, 0.010548286695742974`,
   0.05042811815008133`, 0.020040040157006934`, 0.11291860729312315`, 0.02130167357779572`,
   0.022401916592283033`, 0.05937398311945144`, 0.049996277582139155`, 0.1260433291933589`,
   0.13518887915012978`, 0.006640606506917134`, 0.18648500213533`, 0.07262979854302545`,
   0.41276308612986806`, 0.05861418428968702 4`, 0.12148202106332945`, 0.26987048262765784`,
   0.04705189263025712`, 0.06340417924448331`, 0.18399851093537906`, 0.07782829731477856`,
   0.025044770203730832`, 0.13465223527295853`, 0.23018702191281123`, 0.07087051256021994`,
   0.016466448276807672`, 0.2252012804809484`, 0.07310878484367096`, 0.014074465378942315`,
   0.10570003753307308`, 0.009223853769699171`, 0.09424474135053598`, 0.08818820728942249`,
   0.05449597336988779`, 0.02373791813583209`, 0.009413192593426642`, 0.24413978210907553`,
   0.08316224586299825`, 0.04608912795215825 6`, 0.33956693020359885`,
   0.089850821195628`, 0.011561112412154502`, 0.04503975205749347`, 0.2582639268986554`,
```

```
0.001845721516394414`, 0.19588339816578926`, 0.029254947197993045`,
0.22038818247620795`, 0.07303963745154962`, 0.023772920471881308`, 0.03931973331396152`,
0.053395877012656634`, 0.04456920758595596`, 0.019905106954980996`,
0.11559507167614341`, 0.07234544720387388`, 0.08642713928347033`, 0.08157378361651227`,
0.05765424406841648`, 0.02121229459459935`, 0.011714542455985872`, 0.01483807040854573`,
0.06828461292090594`, 0.09067115344897794`, 0.3338798906462406`, 0.04487176724439002`,
0.006912821101001466`, 0.09868503382227532`, 0.1017886262949806`, 0.13746698585246908`,
0.05110536745035699`, 0.10843570459039348`, 0.06390187999398254`, 0.043386313154534825`,
0.21881732610611743`, 0.02034232079159844`, 0.002282888642637737`,
0.07757148082250877`, 0.009008546045143164`, 0.04988611041248321`, 0.12608597267326244`,
0.001753129951205073`, 0.015401594308821062`, 0.08497108136791583`,
0.13182424103841542`, 0.08135380804226125`, 0.11985862931885964`, 0.14728252959677407`,
0.05233584759001518`, 0.020296796102226095`, 0.08339199229567158`, 0.15018892727621944`,
0.11320482496703634`, 0.004929849932102611`, 0.030150360828894968`,
0.09161468640102331`, 0.07247295981080948`, 0.024638054880529826`,
0.15012304207917515`, 0.04757543723888899`, 0.19725291484801002`, 0.0354323249817157`,
0.17886712987933906`, 0.09470899620115891`, 0.015633928448647947`,
0.05728784008628413`, 0.021149636533166354`, 0.007825214502252547`,
0.05700537319742431`, 0.018159129470547354`, 0.10671095473022894`,
0.052361356864442946`, 0.004862594363425698`, 0.04643488355350014`,
0.10751358450843625`, 0.1362075100414313`, 0.23033009290242934`, 0.05211096609330643`,
0.048167442574141045`, 0.03493631990934665`, 0.07991841476810262`, 0.2565064210477877`,
0.056705550771185866`, 0.041803784269080914`, 0.027586584895162847`,
0.2357306271061681`, 0.17398768503567363`, 0.03072574191612392`, 0.016056749604105466`,
0.1363892118035187`, 0.05697096957716956`, 0.08230944944103274`, 0.07198743057054578`,
0.0018705683923403958`, 0.01182795728493418`, 0.40563577184488925`,
0.14528046049415955`, 0.031683127688862174`, 0.031019797919321888`,
0.06071110232869761`, 0.05845097148687713`, 0.2443274383009821`, 0.3084291177781131`,
0.0025512732970496724`, 0.022920219950319766`, 0.11514859760248128`,
0.11170173740717453`, 0.08744428671435751`, 0.04466432705706432`, 0.006053467735036677`,
0.13815612972137645`, 0.03578244612252999`, 0.12197769778777545`, 0.04895672812845912`,
0.04178710698877259`, 0.01031070945870479`, 0.003585176333015315`, 0.08837025374996933`,
0.10765526119035328`, 0.13293294529116684`, 0.22426900031546623`, 0.03492684918650012`,
0.064325124688272`, 0.06996610099788127`, 0.06685681643939785`, 0.006229418542580043`,
0.0007372819700430921`, 0.00042485712935468647`, 0.18678193571821478`,
0.05307608074268565`, 0.08686171439635243`, 0.03850153854833844`, 0.01797314449295057`,
0.010337782675126722`, 0.2242078743386014`, 0.013265499706055941`, 0.15393510807976707`,
0.0709558044747955`, 0.07092132149442211`, 0.011269837825784198`, 0.08709225970175254`,
0.09315875468787692`, 0.011349991508477784`, 0.13959151099230188`,
0.020663084506969357`, 0.06468832694326283`, 0.00736772835780695`, 0.05812615419901117`,
0.12338448777676873`, 0.07582727782756535`, 0.09619956074949612`, 0.07782322087210797`,
0.002895367130992011`, 0.03853258224322587`, 0.012033157869333039`,
0.08691534050924866`, 0.09102016905951496`, 0.18595157318658376`, 0.028085228953894467`,
0.02571361720363191`, 0.17676115693166633`, 0.19634871321502748`, 0.13039048529937136`,
0.0198337507201804`, 0.09572518659959074`, 0.08911202172863561`, 0.09572041502433273`,
0.07605636012926886`, 0.22404520685297025`, 0.07011128201359357`, 0.027957880591808502`,
0.1908408567042673`, 0.0226704478566824`, 0.1200984940393503`, 0.0671465237677161`,
```

```
0.10011002184047667`, 0.021397724946114`, 0.09042100213852125`, 0.0681566051106101 4`,
0.12370201031703568`, 0.07897089712308399`, 0.1298103392963639`, 0.0497205157657811 1`,
0.05984614876581862`, 0.11862319212390261`, 0.07189460675034552`, 0.08633260449211115`,
0.002337370749790977`, 0.001601460443605651`, 0.01358278954202532 4`,
0.34133181797338136`, 0.012202731668999319`, 0.11745428736845635`, 0.1592502283332045`,
0.06897012545550137`, 0.09958394548341781`, 0.02635587659755446`, 0.0890026859365772`,
0.19114695848243027`, 0.10644558058025887`, 0.048355552662851825`, 0.03867078763650651`,
0.13182544792914738`, 0.23449490835204442`, 0.08841019729331348`, 0.050875315296079845`,
0.05025616179162224`, 0.012312583111424896`, 0.04344646481716098`, 0.06466349876360136`,
0.022240939541930807`, 0.07506942678060664`, 0.005973574544063427`,
0.034757684433484315`, 0.005337472962901332`, 0.026061253660024618`,
0.1582749835667611`, 0.07024726504131697`, 0.04727937070939695`, 0.041212311020921454`,
0.09943640121200233`, 0.028082852477776477`, 0.031800891805282915`,
0.022243998225758146`, 0.05685982374993542`, 0.03211055149428075`,
0.1104406729885982`, 0.040190608045716114`, 0.0237711234489349`, 0.15643571344830945`,
0.23335275809118577`, 0.25206004588097863`, 0.16741418092977822`, 0.1460981332484746`,
0.005554385220726165`, 0.010622677256137083`, 0.059604188624287485`,
0.05122472873793097`, 0.05421892851748007`, 0.05943845325829362`, 0.03907678583310773`,
0.05569961857560133`, 0.022188766800331102`, 0.10425381731853146`, 0.10972932720107767`,
0.0390309902559725 8`, 0.03658916362770861`, 0.06963758835664327`, 0.07525802955461706`,
0.17553808779759575`, 0.021496999015783197`, 0.06630748727203353`, 0.2504519231479152`,
0.02106772898161155`, 0.09828628238432187`, 0.00896369342038081 9`,
0.004885526824956441`, 0.0033490535961217716`, 0.009604628749154973`,
0.031785829294156825`, 0.05436476682927451`, 0.027731529339855555`,
0.04966289070598242`, 0.06946688760946905`, 0.02914798564691675`, 0.09647737722510955`,
0.019339068520185482`, 0.026327082726986344`, 0.030612450181564218`,
0.0011656407623632976`, 0.097020213808844 2`, 0.010666870713809534`,
0.06439448750676871`, 0.1269665200113848`, 0.015659133503467444`, 0.059952570148255335`,
0.009861961350595753`, 0.12487617477796636`, 0.026071693499343244`,
0.012540753509291722`, 0.0031817645165900968`, 0.0033970015435181525`,
0.042633687013771224`, 0.023744993384910606`, 0.12412925487293269`,
0.10211215002403713`, 0.012833513328999823`, 0.20914957927106337`,
0.021856273602169334`, 0.08518294739882216`, 0.054052612198223216`,
0.1124444463617302`, 0.0023878136428712605`, 0.0921643132035803`, 0.0987943470198615 9`,
0.011315550240603585`, 0.21823609687004805`, 0.027011899653441137`,
0.10816729236836295`, 0.014994448534586528`, 0.16072250380010736`,
0.019966670182296233`, 0.07805505194442235`, 0.1516197034028032`, 0.00587371823841655`,
0.03958896668157025`, 0.01309879481066764`, 0.04550400393064221`, 0.08260824167638457`,
0.0025638908890102054`, 0.04534832192693787`, 0.19033858030147263`,
0.006835663914783465`, 0.013659084220883194`, 0.004446863035751444`,
0.0056018759112590 3`, 0.13044864361506275`, 0.02680386808959284`, 0.17196148360282393`,
0.10774817690047944`, 0.17091907384085858`, 0.21169683123486224`, 0.023830088289395403`,
0.0906512971499089`, 0.005438793436488682`, 0.06353035072048843`, 0.06646488244872166`,
0.04502644756405811`, 0.19744134862175158`, 0.022416372139109398`, 0.07661625416224596`,
0.03713457568246325`, 0.002761819963988091`, 0.05954664677164597`, 0.10589023253971605`,
0.02298677269383468`, 0.14169551926394405`, 0.01917971253085128`, 0.049750463660632877`,
0.06987703819641987`, 0.09862253832939528`, 0.22435411313621387`, 0.11510552319465979`,
```

```
0.056850250272995395`, 0.0792987832621478`, 0.01378206041786628`, 0.054930270601357335`,
0.02456678118734301`, 0.00842485904985989`, 0.1271833879105442`, 0.01601490387004966`,
0.08366508570590009`, 0.05158242787425294`, 0.0032679953305752235`,
0.09976253587982355`, 0.04384319087127049`, 0.03319147443633237`, 0.025864298187574894`,
0.024235058518988047`, 0.0589947579111899`, 0.06113942410034057`, 0.1784474153464483`,
0.1014734053859587`, 0.06340124026370007`, 0.020169489641513404`, 0.06725797452768702`,
0.13394966063729155`, 0.0757320302286874`, 0.16579074001783525`};
```

In[105]:=

```
betaDistributionPlot = Show[Histogram[betaSampleB,
   Automatic, "ProbabilityDensity", Frame → {{True, False}, {True, False}},
   FrameLabel → {{"Probability Density", ""}, {"ϵi", ""}},
   LabelStyle → {Bold, FontFamily → "Arial", FontSize → 18},
   FrameStyle → {{Directive[Thick, Black], None}, {Directive[Thick, Black], None}},
   Epilog → {Text[Style["B", Bold, FontFamily → "Arial", FontSize → 16], {0.03, 10}]},
   ImageSize → 550], Plot[PDF[BetaDistribution[α, β] /. params, x],
   {x, 0, 1}, PlotStyle → {Thick, Blue}, PlotRange → {{0, 0.7}, All}]]
```

Out[105]=

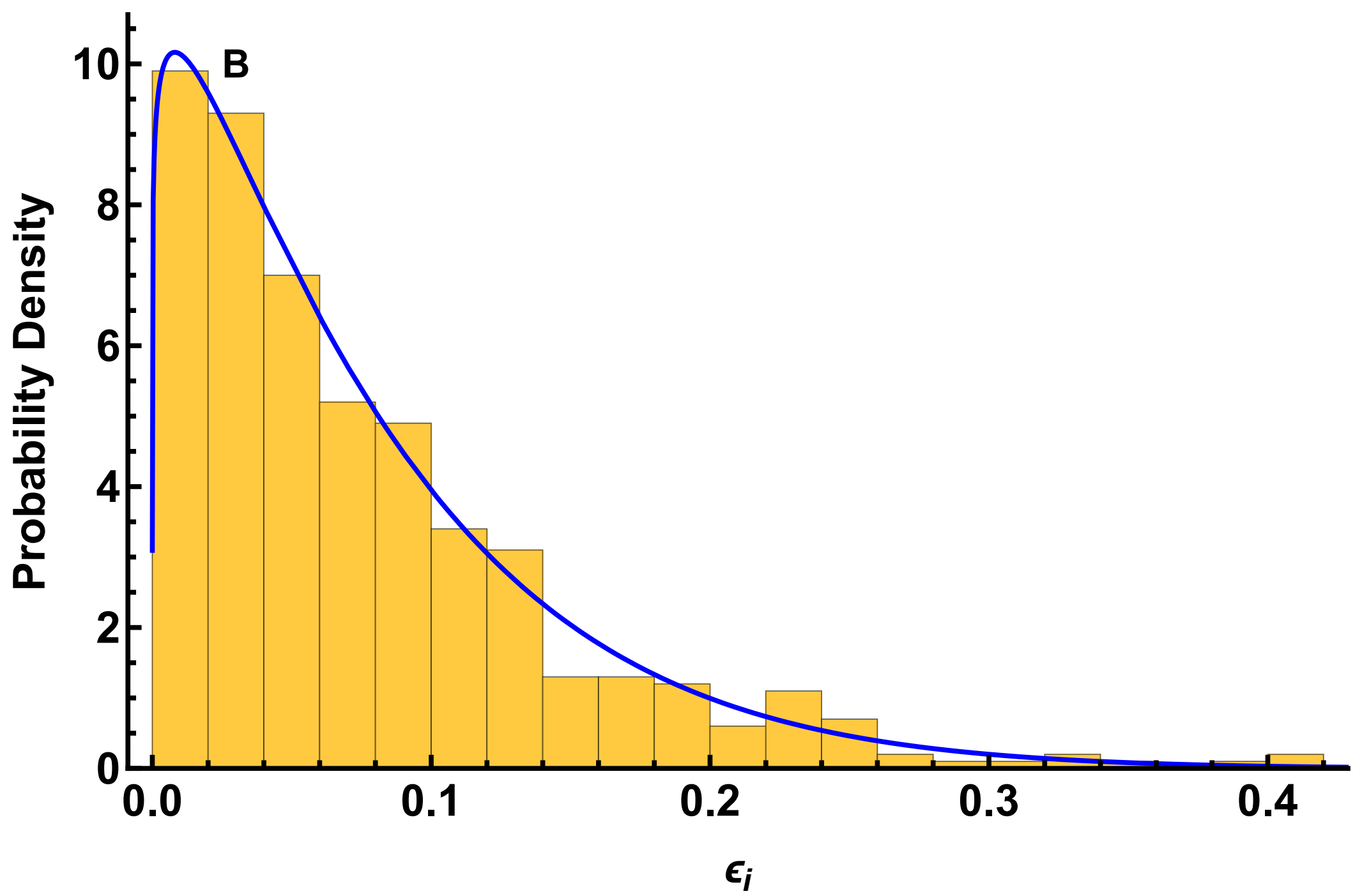

In[106]:=

```
soln500betaB = solveODEs[0.71, 24, params, betaSampleB];
```

In[107]:=

```
Rasterize[xiBetaPlotB = ListLinePlot[
   Evaluate[Table[{t, x[i][t]}, {i, 1, 500}, {t, 0.71, 24, 0.1}] /. soln500betaB],
   PlotRange → All, Frame → {{True, False}, {True, False}},
   FrameLabel → {"Time (d)", "xi(t) concentration (μM)"}, LabelStyle →
    {Bold, FontFamily → "Arial", FontSize → 18, FontColor → Black}, PlotStyle → Thick,
   FrameStyle → {{Directive[Thick, Black], None}, {Directive[Thick, Black], None}},
   Epilog → {Text[Style["B", Bold, FontFamily → "Arial", FontSize → 16], {2, 70}]},
   ImageSize → 550], LightDark → "Light"]
```

Out[107]=

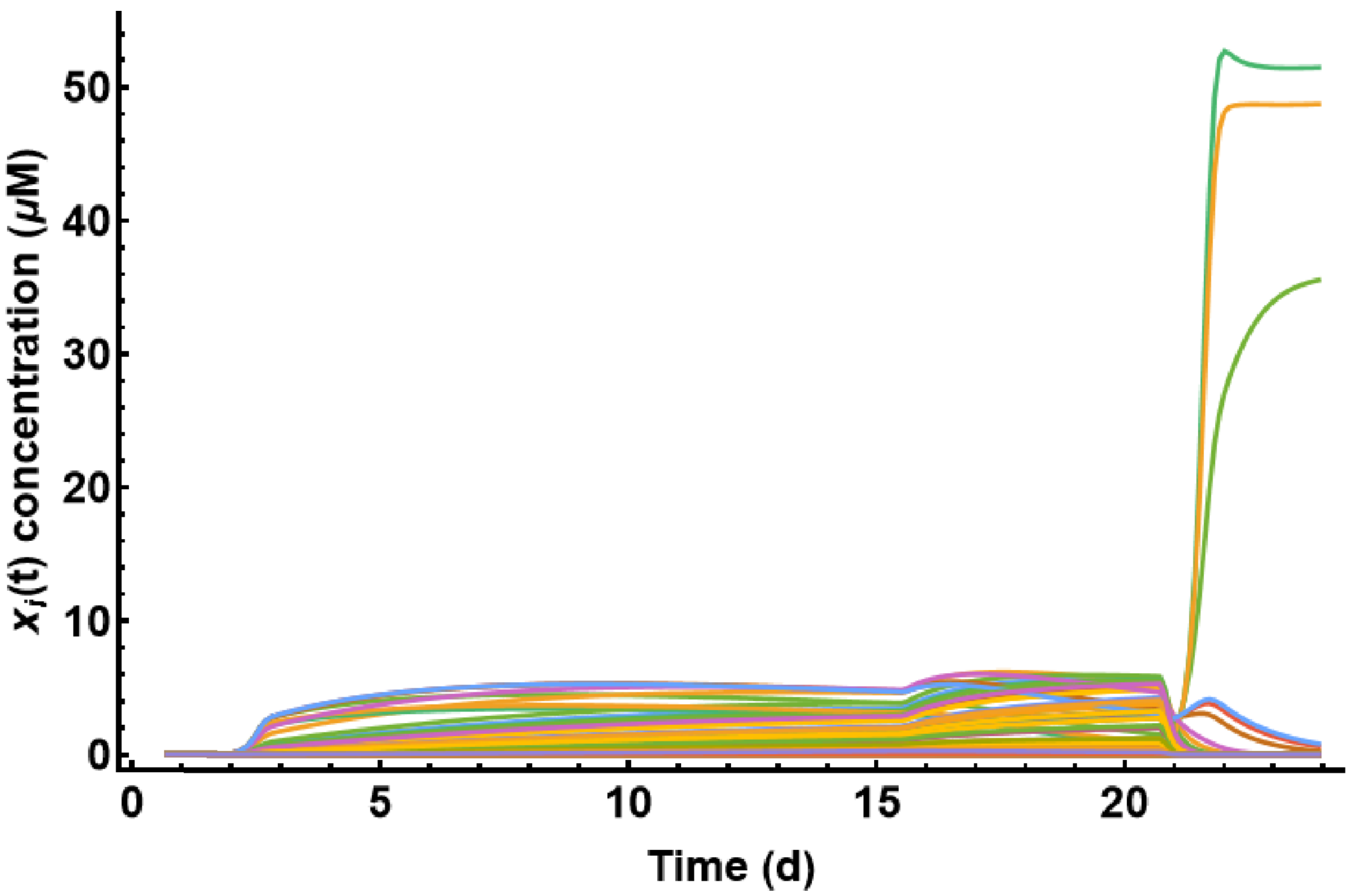

In[108]:=

```
times = {0.8, 6.8, 9.5, 10.5, 15.5, 16.5, 17.6, 18.8, 19.8, 21.5, 21.8, 22.4, 22.8, 23.5};
betaASVsB =
  Prepend[Evaluate[Table[Table[x[i][t], {i, 1, 500}] /. soln500betaB /. t → times⟦j⟧,
     {j, 1, Length[times]}]], Evaluate[Table["ASV_" <> ToString[i], {i, 500}]]]ᵀ;
betaASVsRelB = Prepend[Table[100 betaASVsB⟦1 ;;, i⟧ / Total[betaASVsB⟦1 ;;, i⟧],
    {i, 2, Dimensions[betaASVsB]⟦2⟧}], betaASVsB⟦1 ;;, 1⟧]ᵀ;
```

In[111]:=

```
asvMinAbunRel = 0.1;
asvBetaMinB = Chop[N[betaASVsRelB], asvMinAbunRel];
(* this sets any ASV that is < asvMinAbunRel to 0 *)
```

In[113]:=
```
Rasterize[xiBarBetaPlotB = xiBarChart[asvBetaMinB[[1 ;;, 2 ;;]]ᵀ,
   {{0.8, 6.8, 9.5, 10.5, 15.5, 16.5, 17.6, 18.8, 19.8, 21.5, 21.8, 22.4, 22.8, 23.5},
    Table["", {14}]}, {1, 5, 6, 9, 10, 14}, "B"], LightDark → "Light"]
```
Out[113]=

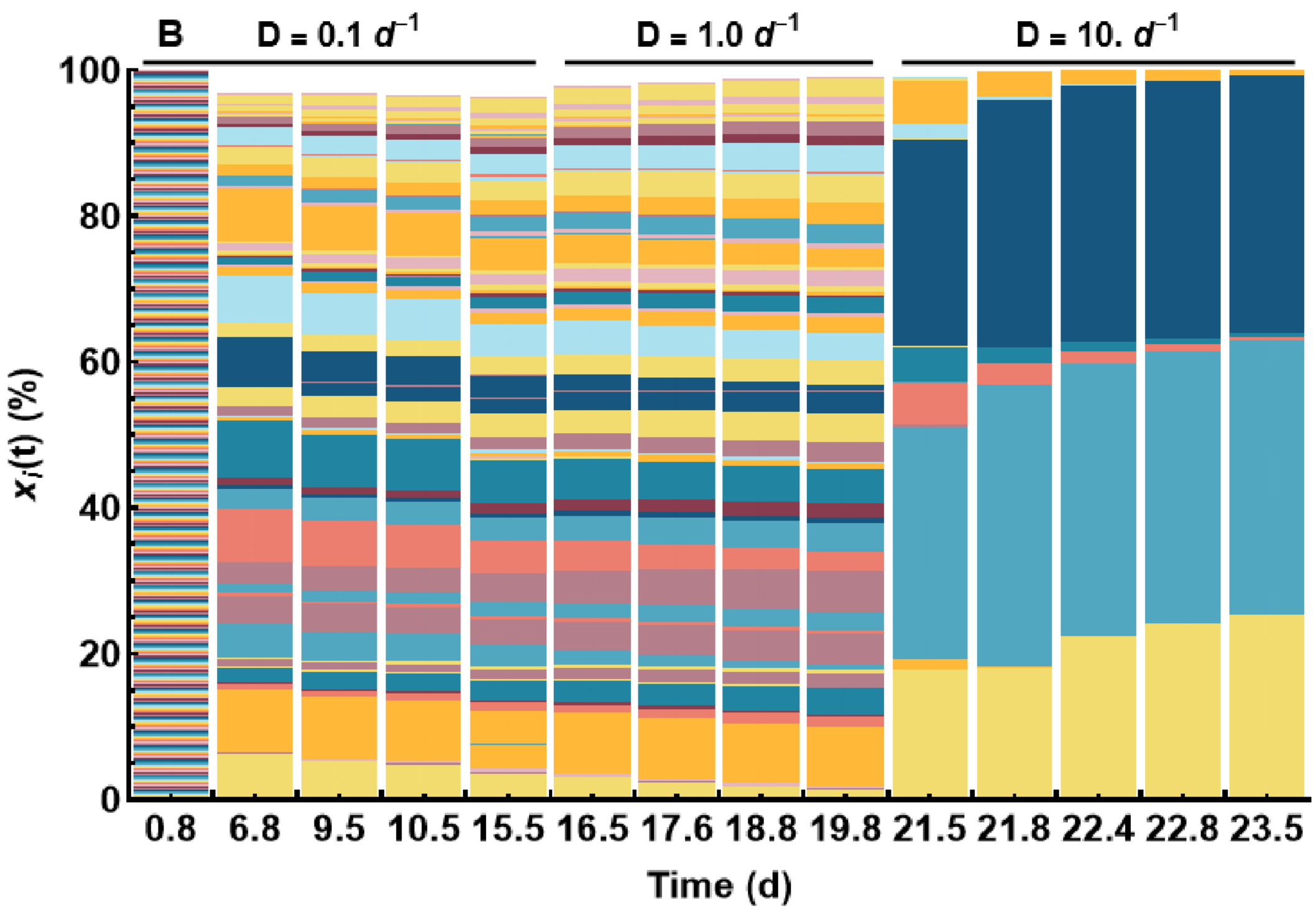

In[114]:=
```
Export["xiBarBetaPlotB.svg", xiBarBetaPlotB, LightDark → "Light"]
```
Out[114]=
```
xiBarBetaPlotB.svg
```
In[115]:=
```
Export["betaASVsRelativeB.csv", Prepend[betaASVsRelB, Flatten[{"ASVs", times}]]]
```
Out[115]=
```
betaASVsRelativeB.csv
```
In[116]:=
```
Export["betaASVsB.csv", Prepend[betaASVsB, Flatten[{"ASVs", times}]]]
```
Out[116]=
```
betaASVsB.csv
```

Simulation comparison to observed pO_2 and DO

Plot the simulated vs observed and export fig.

In[117]:=

```
Rasterize[pO2andDOFig = GraphicsRow[
  {Show[ListLinePlot[o2Data, PlotRange → {{0, 23.6}, All}, PlotStyle → {Black, Gray},
     PlotLegends → Placed[LineLegend[{Black, Gray, Blue, Red},
        {"MC 1", "MC 2", "Uniform", "Beta"}], Center], Epilog → {Text[Style["A", Bold,
         FontFamily → "Arial", FontSize → 16, FontColor → Black], {23, 21.1}]}], Plot[
     Evaluate[100 pO2[t] /. soln500beta], {t, 0.71, 25}, PlotStyle → Red, PlotRange → All],
    Plot[Evaluate[100 pO2[t] /. soln500], {t, 0.71, 24}, PlotStyle → Blue, PlotRange → All],
    Frame → {{True, False}, {True, False}}, FrameLabel → {"Time (d)", "pO2 (%)"},
    FrameStyle → {{Directive[Thick, Black], None}, {Directive[Thick, Black], None}},
    LabelStyle → {Bold, FontFamily → "Arial", FontSize → 16, FontColor → Black},
    PlotRange → {{0, 23.6}, {20, 21.1}}, PlotLegends → Placed[LineLegend[
       {Black, Gray, Blue, Red}, {"MC 1", "MC 2", "Uniform", "Beta"}], Center]],
   Show[ListLinePlot[doData,
     PlotRange → {{0, 23.6}, All}, PlotStyle → {Black, Gray}, PlotLegends → Placed[
       LineLegend[{Black, Gray, Blue, Red}, {"MC 1", "MC 2", "Uniform", "Beta"}], Center],
     Epilog → {Text[Style["B", Bold, FontFamily → "Arial", FontSize → 16,
         FontColor → Black], {23, 270}]}],
    Plot[Evaluate[o2[t] /. soln500beta], {t, 0.71, 24}, PlotStyle → Red, PlotRange → All],
    Plot[Evaluate[o2[t] /. soln500], {t, 0.71, 24}, PlotStyle → Blue, PlotRange → All],
    Frame → {{True, False}, {True, False}},
    FrameStyle → {{Directive[Thick, Black], None}, {Directive[Thick, Black], None}},
    FrameLabel → {"Time (d)", "DO (μM)"},
    LabelStyle → {Bold, FontFamily → "Arial", FontSize → 16, FontColor → Black},
    PlotRange → {{0, 23.6}, {0, 275}}]}, ImageSize → 1024], LightDark → "Light"]
```

Out[117]=

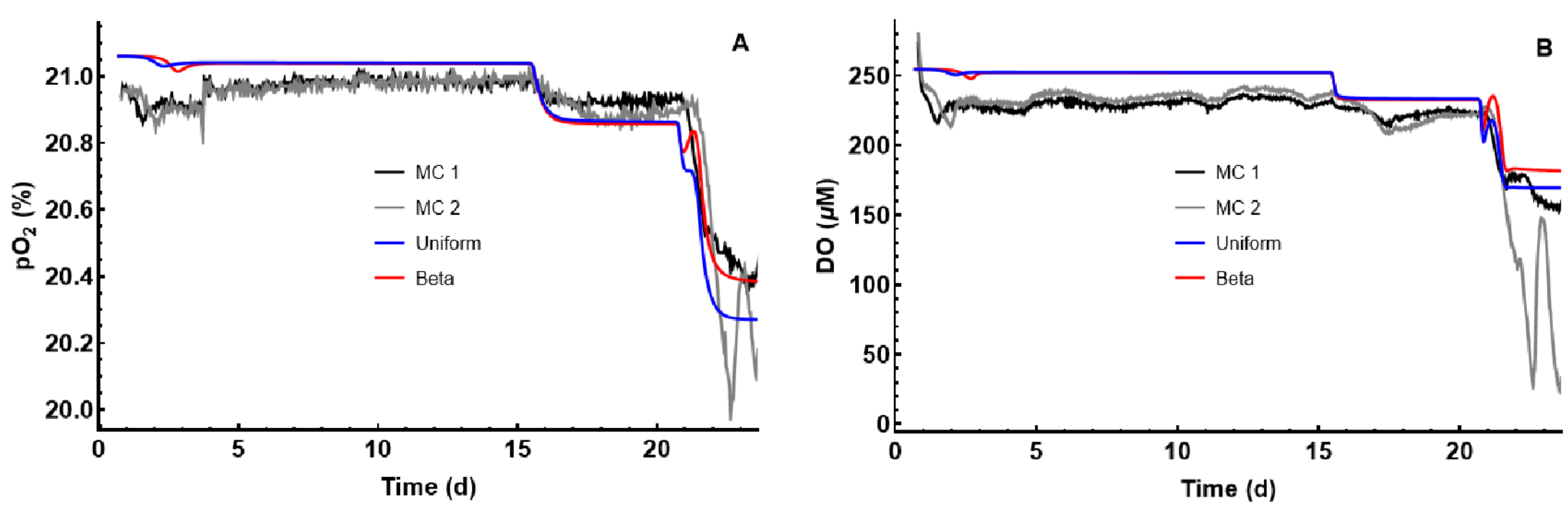

In[118]:=

```
Export["pO2andDOFig.svg", pO2andDOFig, LightDark → "Light"]
```

Out[118]=

```
pO2andDOFig.svg
```

In[119]:=

```
Rasterize[
 pO2Fig = Show[ListLinePlot[o2Data, PlotRange → {{0, 23.6}, All}, PlotStyle → {Black, Gray},
    PlotLegends → Placed[LineLegend[{Black, Gray, Blue, Red}, {"MC 1",
        "MC 2", "Uniform", "Beta"}], Center], Epilog → {Text[Style["A", Bold,
        FontFamily → "Arial", FontSize → 16, FontColor → Black], {23, 21.1}]}],
   Plot[Evaluate[100 pO2[t] /. soln500beta], {t, 0.71, 25}, PlotStyle → Red, PlotRange → All],
   Plot[Evaluate[100 pO2[t] /. soln500], {t, 0.71, 24}, PlotStyle → Blue, PlotRange → All],
   Frame → {{True, False}, {True, False}}, FrameLabel → {"Time (d)", "pO2 (%)"},
   FrameStyle → {{Directive[Thick, Black], None}, {Directive[Thick, Black], None}},
   LabelStyle → {Bold, FontFamily → "Arial", FontSize → 16, FontColor → Black},
   PlotRange → {{0, 23.6}, {20, 21.1}}, PlotLegends →
    Placed[LineLegend[{Black, Gray, Blue, Red}, {"MC 1", "MC 2", "Uniform", "Beta"}],
     Center], ImageSize → 550], LightDark → "Light"]
```

Out[119]=

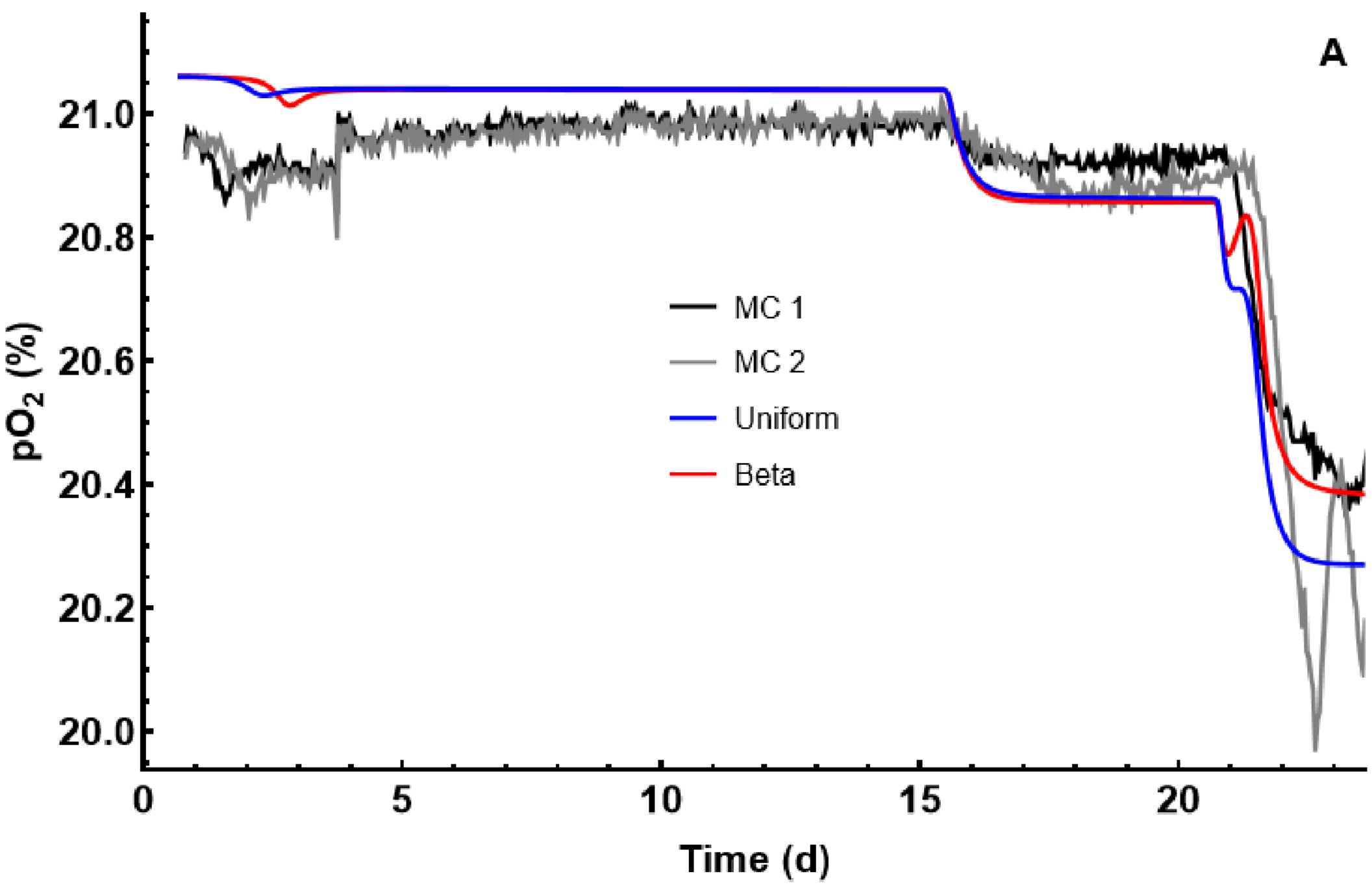

In[120]:=

```
Export["pO2Fig.svg", pO2Fig, LightDark → "Light"]
```

Out[120]=

pO2Fig.svg

In[121]:=

```
Rasterize[
 DOFig = Show[ListLinePlot[doData, PlotRange → {{0, 23.6}, All}, PlotStyle → {Black, Gray},
     PlotLegends → Placed[LineLegend[{Black, Gray, Blue, Red}, {"MC 1",
          "MC 2", "Uniform", "Beta"}], Center], Epilog → {Text[Style["B", Bold,
          FontFamily → "Arial", FontSize → 16, FontColor → Black], {23, 270}]}],
    Plot[Evaluate[o2[t] /. soln500beta], {t, 0.71, 24}, PlotStyle → Red, PlotRange → All],
    Plot[Evaluate[o2[t] /. soln500], {t, 0.71, 24}, PlotStyle → Blue, PlotRange → All],
    Frame → {{True, False}, {True, False}},
    FrameStyle → {{Directive[Thick, Black], None}, {Directive[Thick, Black], None}},
    FrameLabel → {"Time (d)", "DO (μM)"},
    LabelStyle → {Bold, FontFamily → "Arial", FontSize → 16, FontColor → Black},
    PlotRange → {{0, 23.6}, {0, 275}}, ImageSize → 550], LightDark → "Light"]
```

Out[121]=

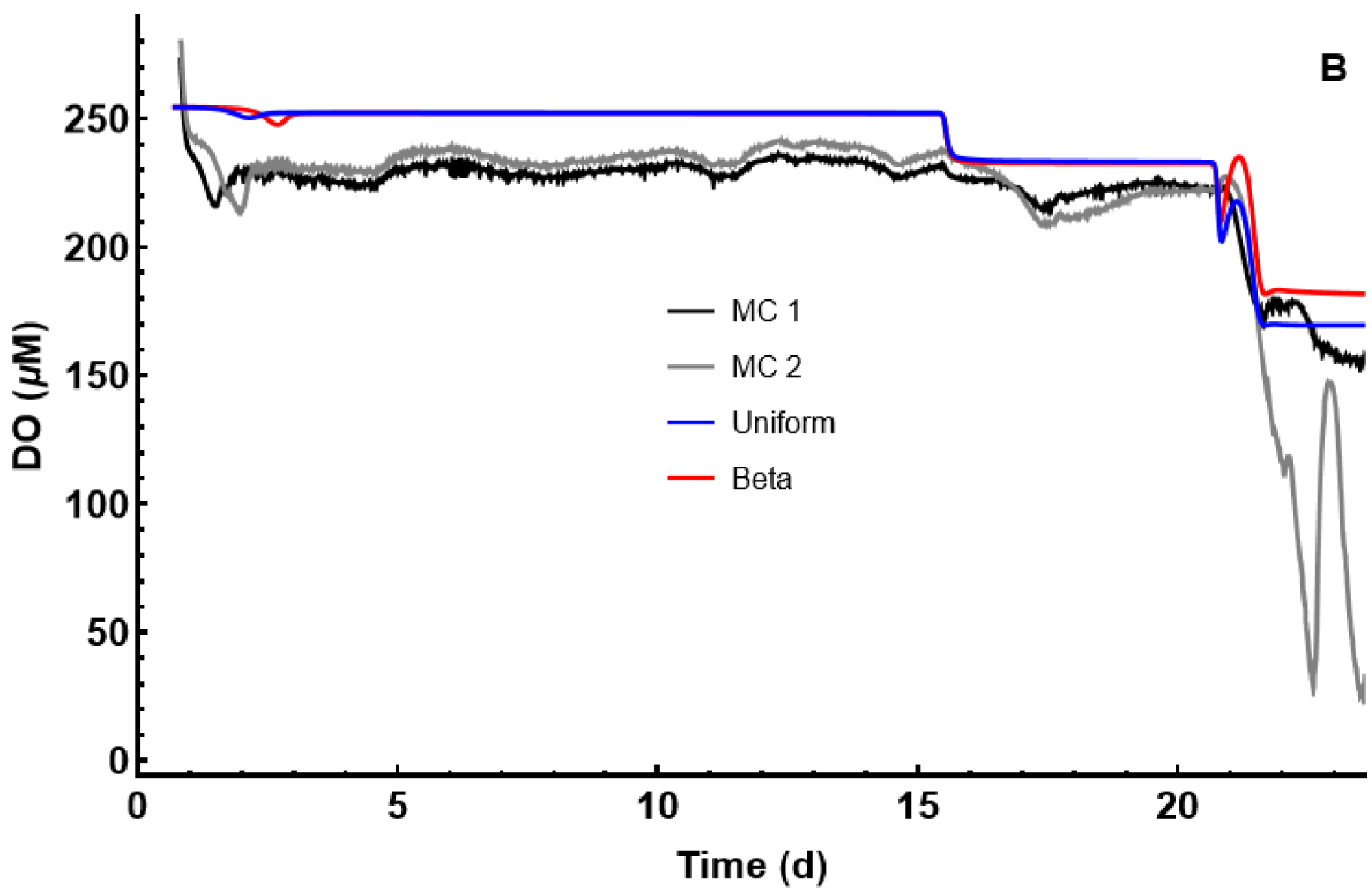

In[122]:=

```
Export["DOFig.svg", DOFig, LightDark → "Light"]
```

Out[122]=

DOFig.svg

Total simulated bacterial concentrations for both distributions

In[123]:=

```
modelBacteriaConcentrationFig = Plot[{Evaluate[Sum[x[i][t], {i, 1, 500}] /. soln500],
    Evaluate[Sum[x[i][t], {i, 1, 500}] /. soln500beta]}, {t, 0.71, 24},
   Frame → {{True, False}, {True, False}}, FrameLabel → {"Time (d)", "Bacteria (μM)"},
   LabelStyle → {Bold, FontFamily → "Arial", FontSize → 16, FontColor → Black},
   PlotRange → {{0, 25}, {0, 250}}, ImageSize → 512, PlotStyle → {{Thick, Blue}, {Thick, Red}},
   FrameStyle → {{Directive[Thick, Black], None}, {Directive[Thick, Black], None}},
   PlotLegends → Placed[LineLegend[{Blue, Red}, {"Uniform", "Beta"}], {0.25, 0.8}]]
```

Out[123]=

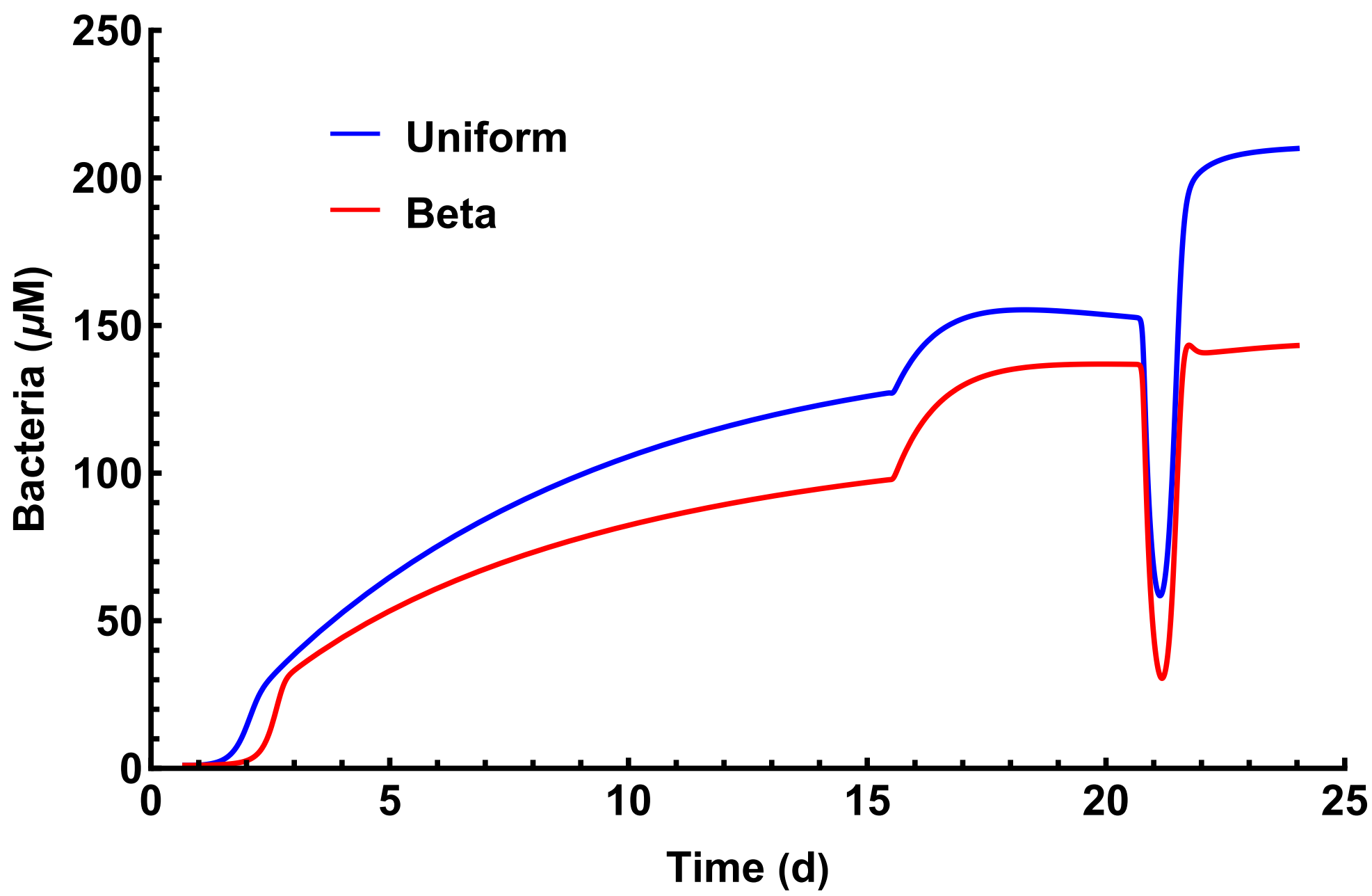

In[124]:=

```
Export["modelBacteriaConcentrationFig.svg",
  modelBacteriaConcentrationFig, LightDark → "Light"]
```

Out[124]=

```
modelBacteriaConcentrationFig.svg
```

Import bacterial cell counts

In[125]:=

```
counts = Import["..\\Data\\BacterialCellCounts.csv"];
```

In[126]:=

```
bacterialCounts =
 ListLinePlot[{counts[[2 ;;, {1, 2, 3}]] /. {x_, y_, z_} → {x, Around[10^-6 y, 10^-6 z]},
   counts[[2 ;;, {1, 4, 5}]] /. {x_, y_, z_} → {x, Around[10^-6 y, 10^-6 z]}},
  PlotMarkers → Automatic, Frame → {{True, False}, {True, False}},
  FrameLabel → {"Time (d)", "Bacteria (10^6 cells mL^-1)"},
  LabelStyle → {Bold, FontFamily → "Arial", FontSize → 16, FontColor → Black},
  PlotRange → {{0, 25}, {0, 20}}, ImageSize → 512, PlotStyle → {{Thick, Blue}, {Thick, Red}},
  FrameStyle → {{Directive[Thick, Black], None}, {Directive[Thick, Black], None}},
  PlotLegends → Placed[LineLegend[{Blue, Red}, {"MC 1", "MC 2"}], {0.1, 0.9}]]
```

Out[126]=

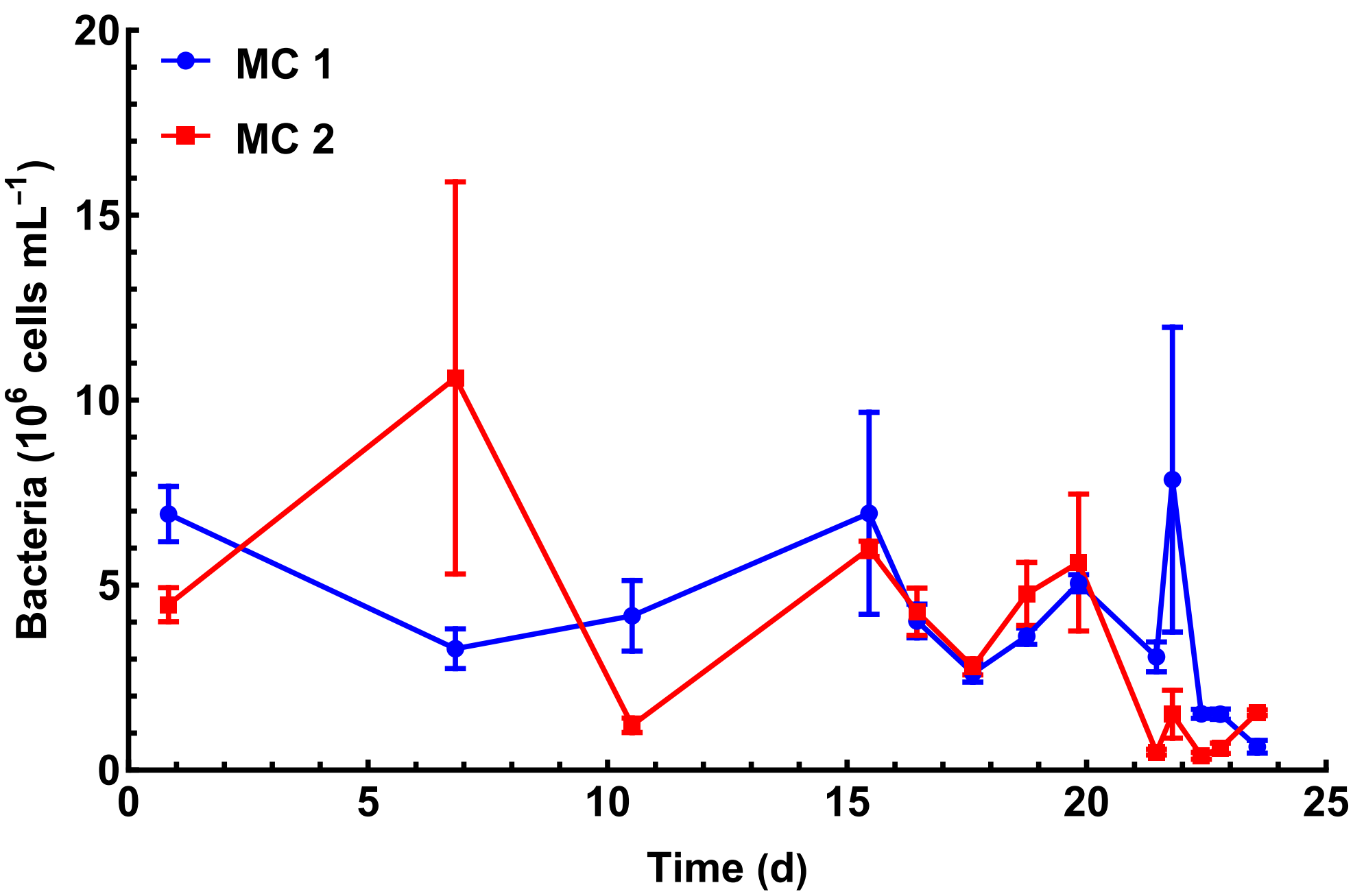

In[127]:=

```
Export["BacterialCounts.svg", bacterialCounts, LightDark → "Light"]
```

Out[127]=

```
BacterialCounts.svg
```

Observed ASV Data Plots

Import all of the ASV data for MC1 and MC2, including taxa names and ASV data for Siders Pond which is in the file Table_S1_ASVs.csv in the Data subdirectory. Drop the header.

Data and plot function

In[128]:=

```
asvData = Drop[Import["..\\Data\\TableS1_ASVs.csv"], 1];
```

In[129]:=
```
Dimensions[asvData]
```
Out[129]=
```
{1420, 34}
```

Get the ASV taxon names

In[130]:=
```
taxaNames = asvData[[ ;; , {29, 30, 31, 32, 33, 34}]];
```

Add Siders ASV data as time “Siders” to each dataset and specify when samples were collected in days, which are slightly different for each MC.

In[131]:=
```
timesMC1 =
  {"Siders", 0.8, 10.5, 15.5, 16.5, 17.6, 18.8, 19.8, 21.5, 21.8, 22.4, 22.8, 23.5};
timesMC2 =
  {"Siders", 0.8, 6.8, 9.5, 10.5, 15.5, 16.5, 17.6, 18.8, 19.8, 21.5, 21.8, 22.4, 23.5};
timesMC12 = {"Siders", 0.8, 6.8, 9.5, 10.5,
   15.5, 16.5, 17.6, 18.8, 19.8, 21.5, 21.8, 22.4, 22.8, 23.5};
```

In[134]:=
```
asvMC1 = Table[0, Dimensions[asvData][[1]], Length[timesMC12] + 1];
(* initialize these to 0 *)
asvMC2 = Table[0, Dimensions[asvData][[1]], Length[timesMC12] + 1];
```

Copy in the asvData, but add in Siders’ ASVs and keep columns 0 for where samples did not sequence.

In[136]:=
```
asvMC1[[ ;; , Flatten[{1, 2, 3, Range[6, 16]}]]] = asvData[[ ;; , Range[1, 14]]];
asvMC2[[ ;; , Flatten[{Range[1, 14], 16}]]] = asvData[[ ;; , Flatten[{1, 2, Range[15, 27]}]]];
```

Define function to name and ASV based on columns in taxaNames above

In[138]:=
```
nameMake[n1_String, n2_String] := n1 <> " " <> n2
```

Define function for generating bar charts with a percentage cutoff. This version of plotASVobs sets ASVs with relative abundances less than minPer to zero instead of removing them. That way, ASV color indexes stay the same. maxLedgend specifies how many of the top ASV taxon names to display, and colorIndex is based on the Mathematica’s Indexed Color Palette Collections

In[139]:=

```
plotASVobs[asvData_, timePts_List, minPer_, maxLedgend_, colorIndex_, dBars_List, fig_] :=
  Module[{asvDataRel, asvDataRelmin, tf, colors},
   (*Relative abundance of an ASV in each sample time, gen in percentage*)
   asvDataRel = Prepend[
      Table[100 asvData⟦ ;; , i⟧ / If[Total[asvData⟦ ;; , i⟧] ≠ 0, Total[asvData⟦ ;; , i⟧], 1],
       {i, 2, Dimensions[asvData]⟦2⟧}], asvData⟦ ;; , 1⟧]ᵀ;
   (*Any ASV less than minPer is set to zero. Note,
   Chop only works on reals, not integers or rational numbers*)
   asvDataRelmin = Chop[N[asvDataRel], minPer];
   (*Get a vector of colors based on colorIndex and add it as the first column *)
   colors = Table[ColorData[colorIndex][i], {i, Length[asvData]}];
   asvDataRelmin = Join[Partition[colors, 1], asvDataRelmin, 2];
   (* sort asvDataRelmin so the largest are first *)
   asvDataRelmin = ReverseSortBy[asvDataRelmin, Max[#⟦3 ;;⟧] &];
   tf[p_] := TableForm[Partition[Flatten[p], UpTo[10]], TableSpacing → {1, 1}];
   TableForm[{BarChart[asvDataRelmin⟦ ;; , 3 ;;⟧ᵀ, ChartLayout → "Stacked",
      ChartLabels → {timePts, Table["", {Length[asvDataRelmin]}]},
      PlotRange → {{0.5, Dimensions[asvDataRelmin]⟦2⟧ - 1.5}, {0, 100}},
      LabelStyle → {Bold, FontFamily → "Arial", FontSize → 16, FontColor → Black},
      ChartStyle → asvDataRelmin⟦ ;; , 1⟧, FrameLabel →
       {"Time (d)", "ASV Rel. Abundance (%)"}, Frame → {{True, False}, {True, False}},
      FrameStyle → {{Directive[Thick, Black], None}, {Directive[Thick, Black], None}},
      ImagePadding → {{All, All}, {All, 30}}, ImageSize → 550,
      Epilog → {Text[Style[fig, Bold, FontFamily → "Arial", FontSize → 16], {1, 105}],
        Thick, Text[Style["D = 0.1 d^-1", Bold, 14], {(dBars⟦1⟧ + dBars⟦2⟧) / 2, 105}],
        Line[{{dBars⟦1⟧ - 0.3, 101}, {dBars⟦2⟧ + 0.3, 101}}],
        Text[Style["D = 1.0 d^-1", Bold, 14], {(dBars⟦3⟧ + dBars⟦4⟧) / 2, 105}],
        Text[Style["D = 10. d^-1", Bold, 14], {(dBars⟦5⟧ + dBars⟦6⟧) / 2, 105}],
        Line[{{dBars⟦3⟧ - 0.3, 101}, {dBars⟦4⟧ + 0.3, 101}}],
        Line[{{dBars⟦5⟧ - 0.3, 101}, {dBars⟦6⟧ + 0.3, 101}}]}],
     SwatchLegend[asvDataRelmin⟦ ;; maxLedgend, 1⟧, asvDataRelmin⟦ ;; maxLedgend, 2⟧,
      LegendMarkerSize → 10, LabelStyle → {FontSize → 8}, LegendLayout → tf]}]
  ];
```

Plot with names from Family

In[140]:=

```
familyMC1 = asvMC1;
familyMC1⟦ ;; , 1⟧ = Thread[nameMake[taxaNames⟦ ;; , 5⟧, asvMC1⟦ ;; , 1⟧]];
familyMC2 = asvMC2;
familyMC2⟦ ;; , 1⟧ = Thread[nameMake[taxaNames⟦ ;; , 5⟧, asvMC2⟦ ;; , 1⟧]];
```

Barcharts

Plot ASVs who’s relative abundance > 0.1%

In[142]:=

```
Rasterize[
 mc1family = plotASVobs[familyMC1, timesMC12, 0.1, 10, 24, {2, 6, 7, 10, 11, 15}, "A"],
 LightDark → "Light"]
```

Out[142]=

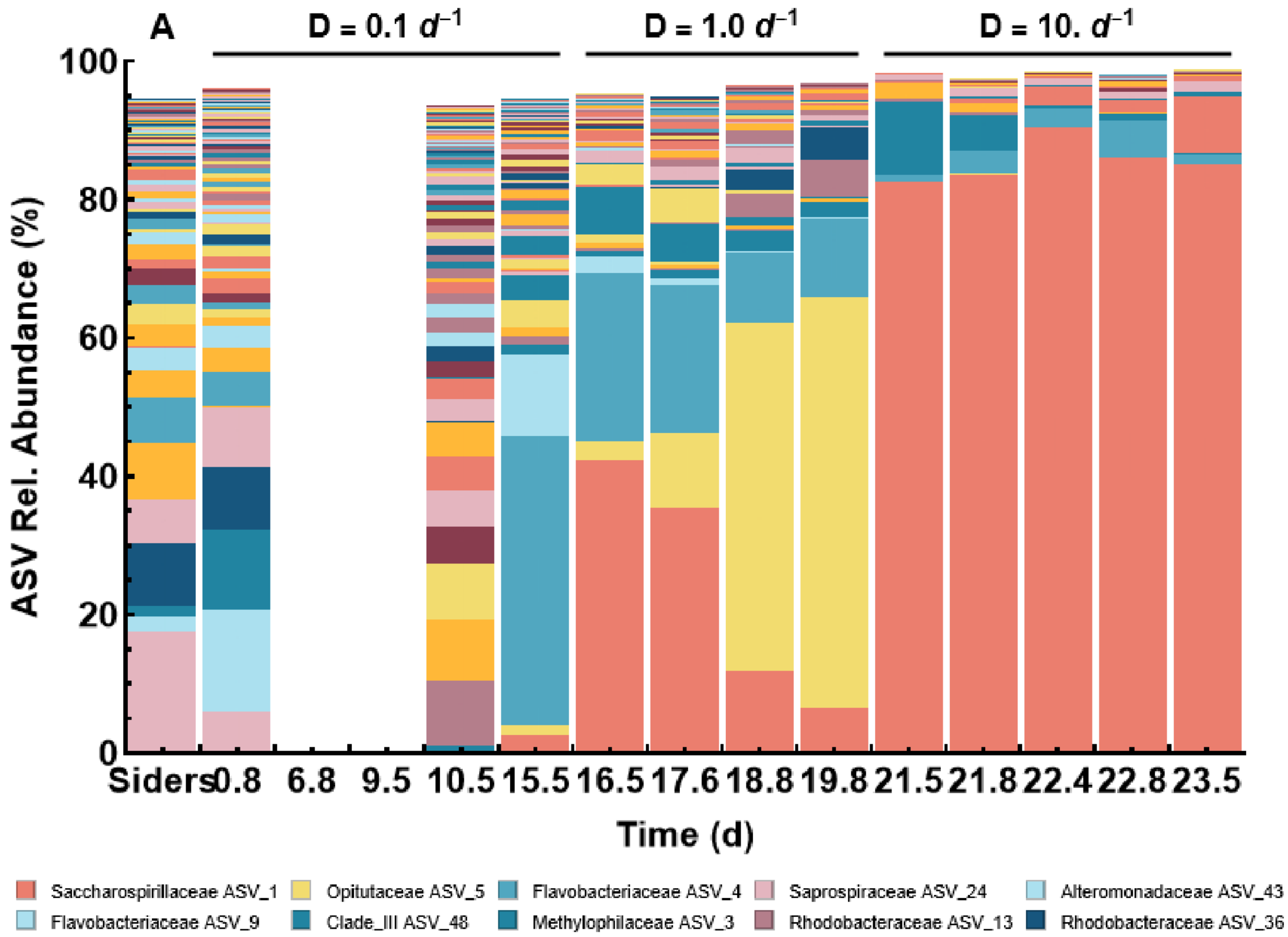

In[143]:=

```
Rasterize[
 mc2family = plotASVobs[familyMC2, timesMC12, 0.1, 10, 24, {2, 6, 7, 10, 11, 15}, "B"],
 LightDark → "Light"]
```

Out[143]=

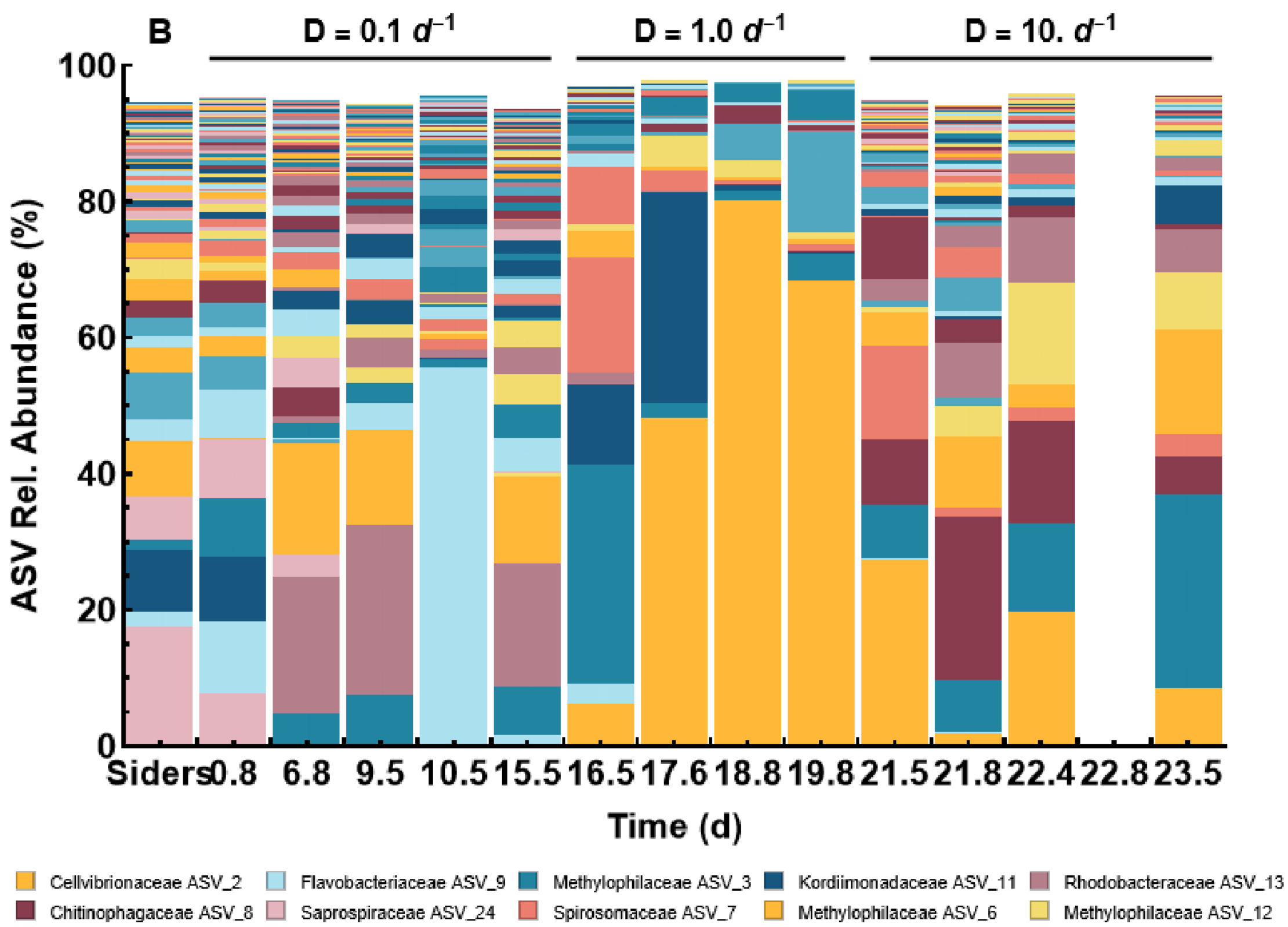

In[144]:=

```
Export["familyMC1_24.svg", mc1family, LightDark → "Light"];
Export["familyMC2_24.svg", mc2family, LightDark → "Light"];
```

Generate a legend based on the top maxLedgend based on both MCs

In[146]:=

```
legendASVobs[asvData_, minPer_, maxLedgend_, colorIndex_, fontSize_] :=
 Module[{asvDataRel, asvDataRelmin, tf, colors},
  (*Relative abundance of an ASV in each sample time, gen in percentage*)
  asvDataRel = Prepend[
     Table[100 asvData⟦;; , i⟧ / If[Total[asvData⟦;; , i⟧] ≠ 0, Total[asvData⟦;; , i⟧], 1],
      {i, 2, Dimensions[asvData]⟦2⟧}], asvData⟦;; , 1⟧]ᵀ;
  (*Any ASV less than minPer is set to zero. Note,
  Chop only works on reals, not integers or rational numbers*)
  asvDataRelmin = Chop[N[asvDataRel], minPer];
  (*Get a vector of colors based on colorIndex and add it as the first column *)
  colors = Table[ColorData[colorIndex][i], {i, Length[asvData]}];
  asvDataRelmin = Join[Partition[colors, 1], asvDataRelmin, 2];
  (* sort asvDataRelmin so the largest are first *)
  asvDataRelmin = ReverseSortBy[asvDataRelmin, Max[#⟦3 ;;⟧] &];
  tf[p_] := TableForm[Partition[Flatten[p], UpTo[10]], TableSpacing → {1, 1}];
  SwatchLegend[asvDataRelmin⟦;; maxLedgend, 1⟧, asvDataRelmin⟦;; maxLedgend, 2⟧,
   LegendMarkerSize → 10, LabelStyle → {FontSize → fontSize}, LegendLayout → tf]
 ]
```

Top ASVs for both MCs

In[147]:=

```
legendFamilyTop30 =
 legendASVobs[Join[familyMC1, familyMC2⟦;; , Range[3, 16]⟧, 2], 0.1, 30, 24, 10]
```

Out[147]=

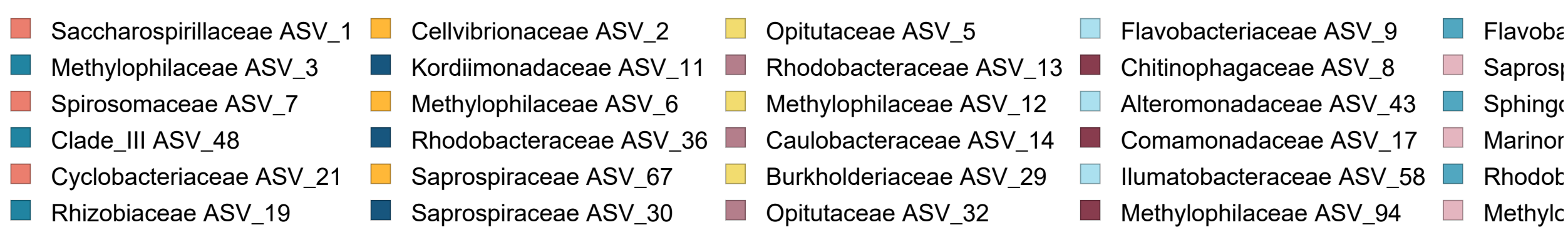

In[148]:=

```
Export["legendFamilyTop30.svg", legendFamilyTop30, LightDark → "Light"]
```

Out[148]=

```
legendFamilyTop30.svg
```

Plot with names from Order

In[149]:=

```
orderMC1 = asvMC1;
orderMC1⟦;; , 1⟧ = Thread[nameMake[taxaNames⟦;; , 4⟧, asvMC1⟦;; , 1⟧]];
orderMC2 = asvMC2;
orderMC2⟦;; , 1⟧ = Thread[nameMake[taxaNames⟦;; , 4⟧, asvMC2⟦;; , 1⟧]];
```

Plot ASVs who’s relative abundance > 0.1%

In[151]:=

```
Export["orderMC1_24b.svg", mc1Order, LightDark → "Light"];
Export["orderMC2_24b.svg", mc2Order, LightDark → "Light"];
```

Conclusions

We see a decrease in α diversity with increasing dilution rate that matches the chemostat data only if the community maximum specific growth rate is skewed like the beta distribution; that is, the natural community appears to be skewed towards gleaners (oligotrophs) over opportunists (copiotrophs).
