# Supplementary material for: Growth characteristics of natural microbial populations are skewed toward bacteria with low specific growth rates
Source: Microbiol Spectr. 2026 Aug 4;14(9):e02193-26. doi: 10.1128/spectrum.02193-26 (PMC13532248; doi:10.1128/spectrum.02193-26)
Supplement: Supplementary file 2 [file spectrum.02193-26-s0002.pdf]

Supplemental Figures

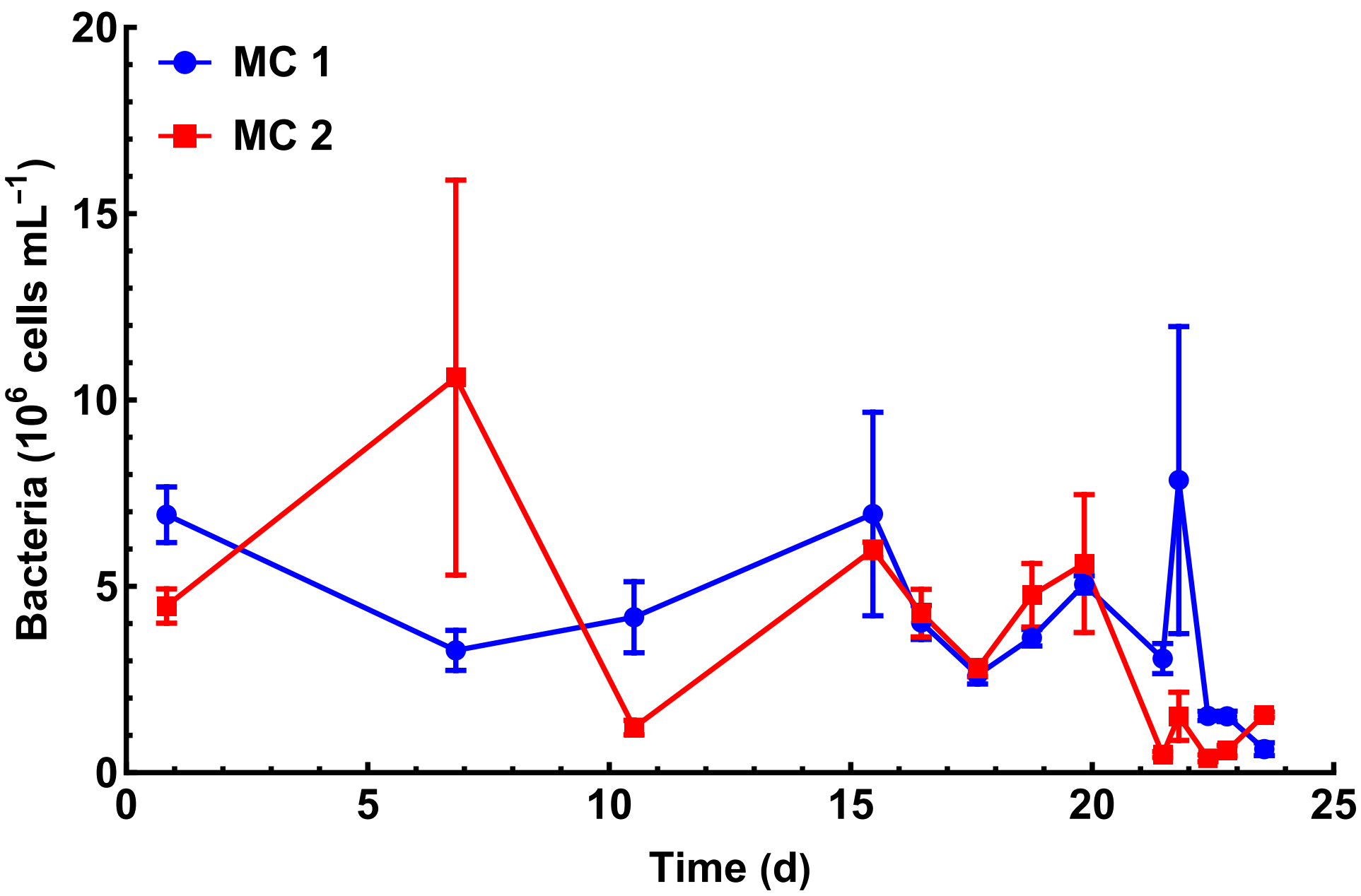

Fig. S1. Bacterial cell counts form MC 1 (blue line) and MC 2 (red line) over time. Error bars represent standard error.

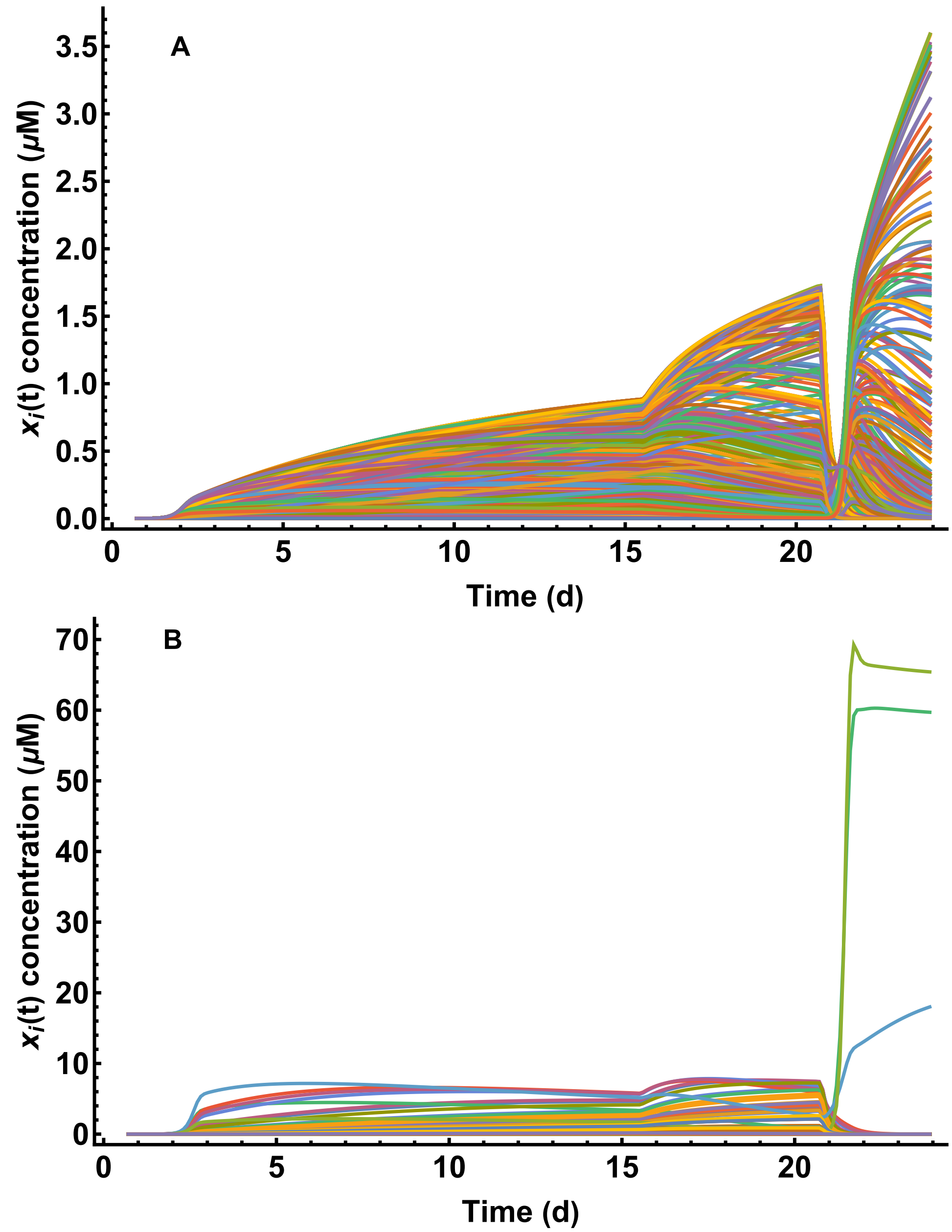

Fig. S2. Simulated ASV absolute concentrations (μM C) versus time for ε_i drawn from (A) uniform and (B) beta probability distributions (See Fig. 1).

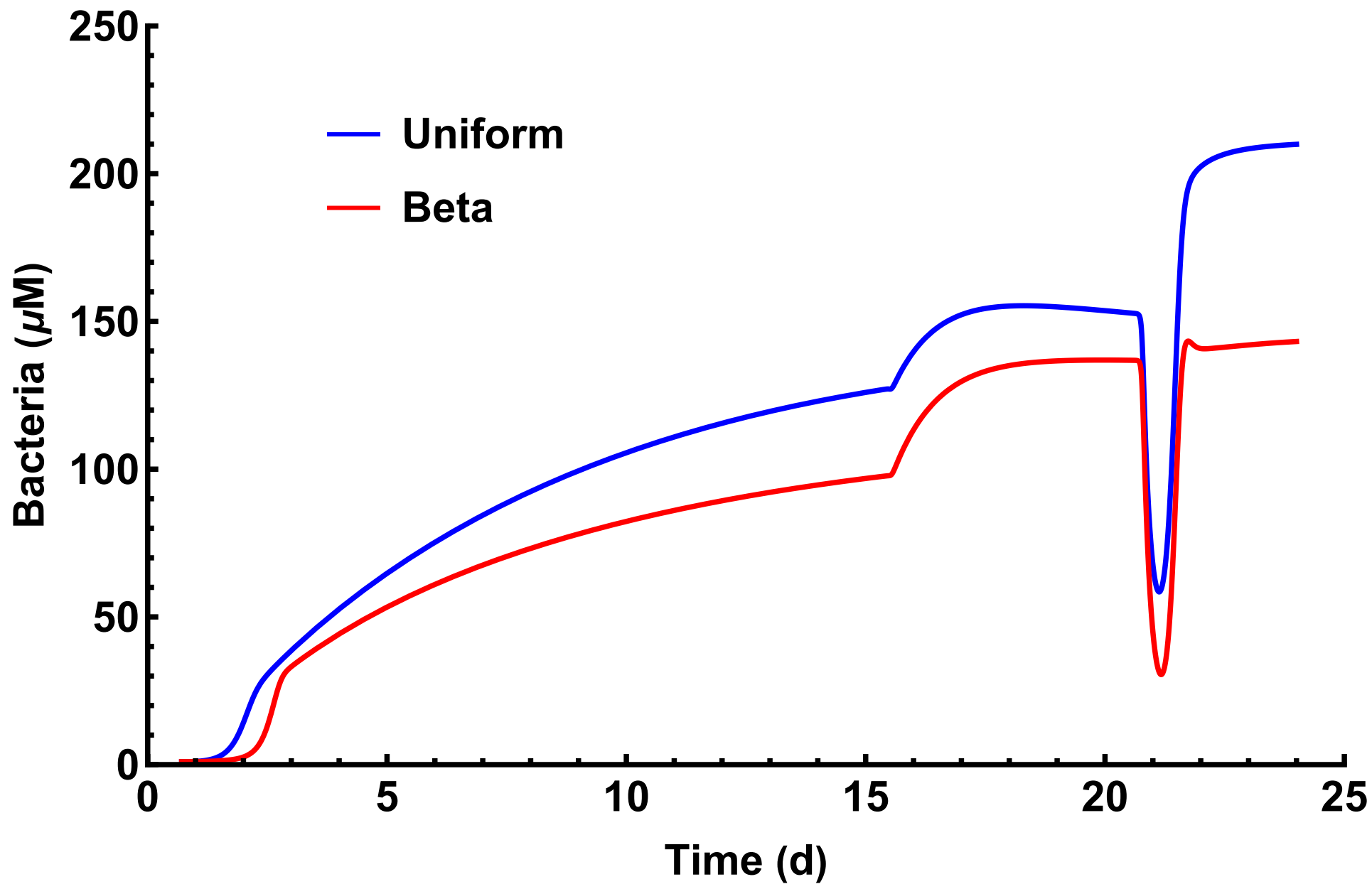

Fig. S3. Simulated bacterial concentration (μM C) versus time (d) for ε_i drawn from uniform (blue line) and beta (red line) probability distributions.

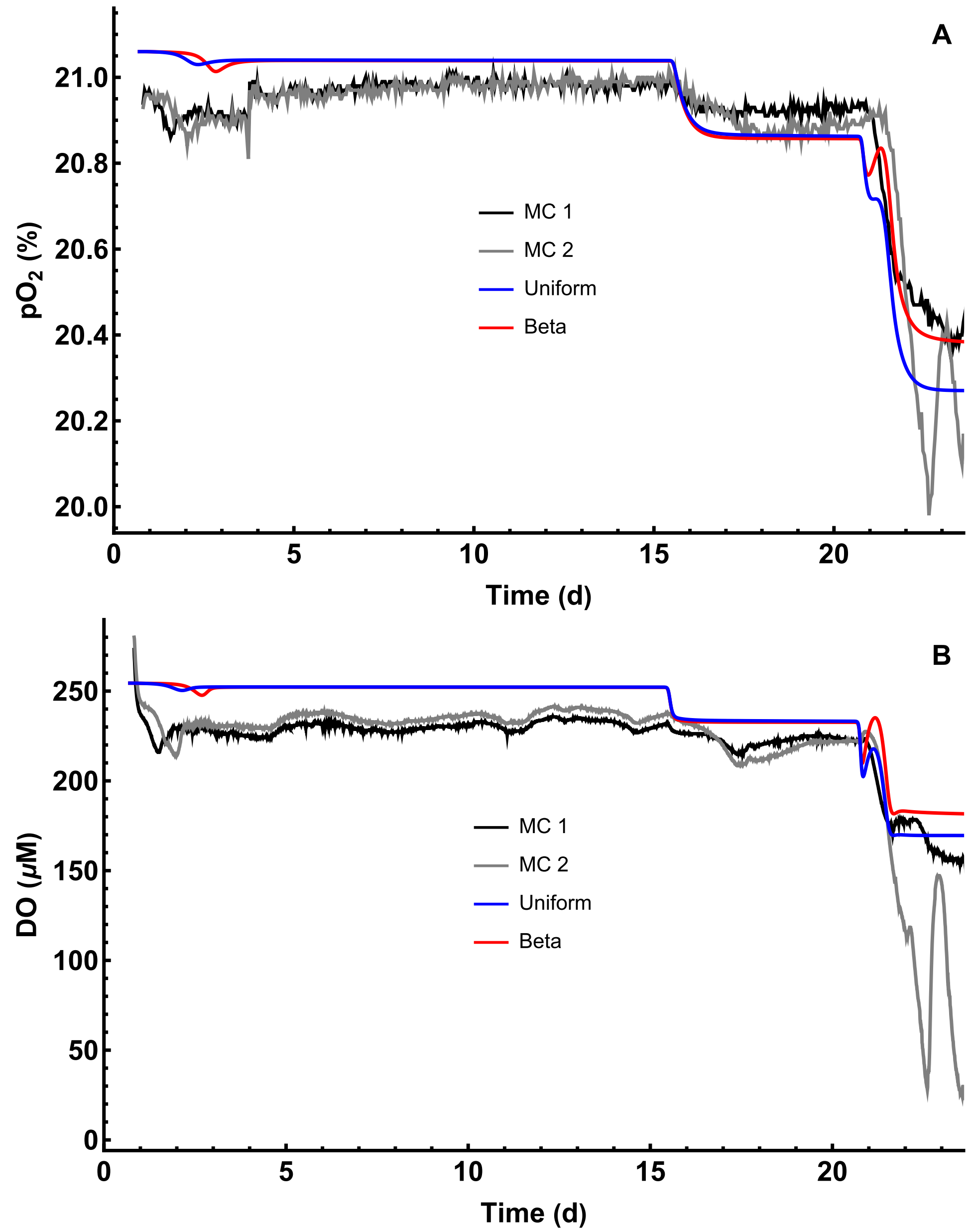

Fig. S4. Simulated (blue and red lines) and observed (black and gray lines) (A) partial pressure of oxygen in the chemostat headspace (pO_2, %) and (B) dissolved oxygen concentration in the media (DO, μM) versus time (d).
